# Prevalence of Cardiovascular Disease in Patients With Potentially Curable Malignancies: A National Registry Dataset Analysis

**DOI:** 10.1016/j.jaccao.2022.03.004

**Published:** 2022-06-21

**Authors:** Nicolò Matteo Luca Battisti, Catherine A. Welch, Michael Sweeting, Mark de Belder, John Deanfield, Clive Weston, Michael D. Peake, David Adlam, Alistair Ring

**Affiliations:** a Breast Unit, Department of Medicine, The Royal Marsden NHS Foundation Trust, London, United Kingdom; b Breast Cancer Research Division, The Institute of Cancer Research, London, United Kingdom; c Biostatistics Research Team, Department of Health Sciences, University of Leicester, Leicester, United Kingdom; d Public Health England, London, United Kingdom; e National Institute for Cardiovascular Outcomes Research, London, United Kingdom; f Barts Health NHS Trust, London, United Kingdom; g Institute of Cardiovascular Sciences, University College London, London, United Kingdom; h Myocardial Ischaemia National Audit Project; i School of Medicine, Swansea University, Swansea, United Kingdom; j Glangwili General Hospital, Carmarthen, United Kingdom; k University of Leicester, Leicester, United Kingdom; l Department of Cardiovascular Sciences, University of Leicester and Leicester National Institute of Health Research Biomedical Research Centre, Glenfield Hospital, Leicester, United Kingdom; m University Hospitals of Leicester NHS Trust, Leicester, United Kingdom

**Keywords:** breast cancer, colorectal cancer, lung cancer, lymphoma, prostate cancer, CVD, cardiovascular disease, DLBCL, diffuse large B-cell lymphoma, HES, Hospital Episode Statistics, ICD-10, International Statistical Classification of Diseases and Related Health Problems-10th Revision, NCRD, National Cancer Registration Dataset, NICOR, National Institute for Cardiovascular Outcomes Research, NSCLC, non-small-cell lung cancer

## Abstract

**Background:**

Although a common challenge for patients and clinicians, there is little population-level evidence on the prevalence of cardiovascular disease (CVD) in individuals diagnosed with potentially curable cancer.

**Objectives:**

We investigated CVD rates in patients with common potentially curable malignancies and evaluated the associations between patient and disease characteristics and CVD prevalence.

**Methods:**

The study included cancer registry patients diagnosed in England with stage I to III breast cancer, stage I to III colon or rectal cancer, stage I to III prostate cancer, stage I to IIIA non-small-cell lung cancer, stage I to IV diffuse large B-cell lymphoma, and stage I to IV Hodgkin lymphoma from 2013 to 2018. Linked hospital records and national CVD databases were used to identify CVD. The rates of CVD were investigated according to tumor type, and associations between patient and disease characteristics and CVD prevalence were determined.

**Results:**

Among the 634,240 patients included, 102,834 (16.2%) had prior CVD. Men, older patients, and those living in deprived areas had higher CVD rates. Prevalence was highest for non-small-cell lung cancer (36.1%) and lowest for breast cancer (7.7%). After adjustment for age, sex, the income domain of the Index of Multiple Deprivation, and Charlson comorbidity index, CVD remained higher in other tumor types compared to breast cancer patients.

**Conclusions:**

There is a significant overlap between cancer and CVD burden. It is essential to consider CVD when evaluating national and international treatment patterns and cancer outcomes.

Cancer is associated with significant morbidity and mortality in England.^1^ Cancer and cardiovascular disease (CVD) survival is improving.^2^^,^^3^ However, they share risk factors and pathophysiological processes^4^ and may coexist.^5^ Furthermore, cancer and its treatment may result in cardiac complications.^6^ CVD may influence cancer management and contribute to disparities^7^ in the UK,^8^, ^9^, ^10^, ^11^, ^12^ in older adults,^13^^,^^14^ and internationally.^2^^,^^15^

Pre-existing CVD in individuals with potentially curable cancer has been described in various countries.^16^, ^17^, ^18^, ^19^ However, this has not been widely reported in England. The impact of cancer-related factors and social deprivation on cancer and CVD has also not been previously assessed. Investigating the intersection of cancer and CVD is central to understanding outcomes, informing cancer policy, and service provision.

We analyzed the prevalence of pre-existing CVD in a cohort of individuals with potentially curable tumors in England, as differences in cancer management due to comorbidities may affect survival. We also assessed the associations between CVD prevalence and patient and tumor characteristics.

## Methods

As part of the Virtual Cardio-Oncology Research Initiative program,^20^ we linked Public Health England National Cancer Registration Dataset (NCRD),^21^ Hospital Episode Statistics (HES),^22^ and National Institute for Cardiovascular Outcomes Research (NICOR)^23^ data to identify CVD recorded in hospital records and registry datasets. We linked English cancer registry data (NCRD) and 6 CVD-specific audits managed by NICOR (Supplemental Table 1). Four NICOR databases were included in this study: the Myocardial Ischaemia National Audit Project,^24^ National Adult Cardiac Surgery Audit,^25^ National Adult Percutaneous Coronary Intervention,^26^ and National Heart Failure Audit.^27^ While the Myocardial Ischaemia National Audit Project and National Heart Failure Audit are audit programs including data on patients with suspected acute coronary syndromes and with heart failure, respectively, the National Adult Cardiac Surgery Audit and National Adult Percutaneous Coronary Intervention collect data on those undergoing cardiac surgery and those undergoing percutaneous coronary procedures, respectively. Patients are included in the audits if they have certain diagnoses or procedures, but they may have other CVD diagnoses that were not the reason they were included in the specific audit. The NICOR audit datasets do not report International Statistical Classification of Diseases and Related Health Problems-10th Revision (ICD-10) codes. To include a wider range of CVD compared with those included in the 4 NICOR audits, we included HES administrative data collected during hospital admissions for remuneration purposes. HES records a wide range of patient information, and diagnoses can be recorded as primary diagnoses or comorbidities. We chose a permissive approach to include CVD in any position to identify all patients with relevant comorbidities. NICOR data are derived from specialist audits restricted to recording information about specific CVD: they include codes related to cardiovascular admissions in a specialist unit. NICOR and HES include diagnoses captured in the inpatient setting. Robust quality assurance checks are in place for the NICOR and HES datasets.^28^^,^^29^ Therefore, CVD prevalence was defined according to either presence of an inpatient hospitalization CVD diagnosis code and/or a NICOR CVD audit record.

NCRD has existing linkages with the National Radiotherapy Dataset and Systemic Anti-Cancer Therapy database. The National Radiotherapy Dataset standard (SCCI0111)^30^ requires all National Health Service radiotherapy providers in England to collect standardized data. National Health Service Trusts providing systemic anticancer therapy submit data to the Systemic Anti-Cancer Therapy database. Data quality is considered sufficient for data analysis from 2013.

This study was reviewed and approved by the Virtual Cardio-Oncology Research Initiative Consortium Project Review Panel. The Virtual Cardio-Oncology Research Initiative research program has received favorable ethical opinion from the Northeast–Newcastle & North Tyneside 2 Research Ethics Committee (reference 18/NE/0123). The study was performed in accordance with the Declaration of Helsinki.

### Identification of the patient cohort

We analyzed NCRD data to identify patients from England with potentially curable cancers. Specifically, we included individuals diagnosed with malignancy (breast cancer, non-small-cell-lung cancer [NSCLC], colon cancer, rectal cancer, prostate cancer, diffuse large B-cell lymphoma [DLBCL], and Hodgkin lymphoma) and with potentially curable cancer stages. We used the ICD-10 codes^31^ to identify the first record of breast cancer (code C50), colon cancer (codes C18 and C19), rectal cancer (code C20), prostate cancer (code C61) and NSCLC (code C34 excluding small-cell morphology codes 8041, 8042, 8043, 8044, or 8045), DLBCL (code C83.3), and Hodgkin lymphoma (code C81) from January 1, 2013, to December 31, 2018.

If patients had >1 tumor diagnosed at different sites, we included the first tumor diagnosed in the analysis. If patients had synchronous diagnoses, we included the tumor with the worst prognosis on the basis of stage, grade, receptor status (for breast cancer), and Gleason group (for prostate cancer) (Supplemental Table 2). We excluded patients with synchronous tumors diagnosed in the same site with similar prognostic features and those with synchronous tumors diagnosed in different sites.

We included patients 25 to 100 years of age at cancer diagnosis, those with residency in England, and those with complete data on vital status, sex, and National Health Service number (to allow linkage). We restricted the analysis to potentially curable tumors (stage I-III breast cancer, stage I-III colon or rectal cancer, stage I-III prostate cancer, stage I-IIIA NSCLC, stage I-IV DLBCL, and stage I-IV Hodgkin lymphoma) (Supplemental Table 3). Patients with missing data on age, sex, National Health Service number, mortality status, or cancer stage were excluded. Data on age, sex, ethnicity, histology, grade, and tumor-node-metastasis stage were extracted at cancer diagnosis. Cancer-specific characteristics were retrieved.

The Index of Multiple Deprivation is the official measure of deprivation in England.^32^ An established methodological framework is followed to derive 7 distinct domains of deprivation, which are weighted and then combined to calculate the Index of Multiple Deprivation at the lower layer super output area (a government-defined geographic region). We extracted the income domain of the Index of Multiple Deprivation for analysis, which was already divided into quintiles of deprivation. Nonincome components of the Index of Multiple Deprivation were not included, as only the income domain was available for analysis.

Using linkages with the National Radiotherapy Dataset, Systemic Anti-Cancer Therapy dataset, and HES,^22^ a database of hospital admissions, surgical procedures, radiation therapy, and systemic anticancer treatments performed in England was extracted. We identified curative treatments (surgery, radiotherapy, and chemotherapy) using previously agreed-upon algorithms.^33^

### Comorbidities

We extracted comorbidities defined within the Charlson comorbidity index,^34^ identified using HES admitted patient care diagnoses recorded within 5 years before cancer diagnosis, and derived a Charlson comorbidity index excluding CVD to avoid counting them in both the CVD exposure and the index (Supplemental Table 4).

We identified CVD comorbidities using the ICD-10 code list (Supplemental Table 5) from diagnoses recorded in any diagnostic position in HES admitted patient care (inpatient) data or if the patient had a record in a NICOR database^23^ within 5 years before cancer diagnosis.^35^ ICD-10 CVD codes were obtained from a Virtual Cardio-Oncology Research Initiative study^36^ and divided into cerebrovascular, stroke (cerebrovascular subgroup), congestive cardiac failure, ischemic heart disease, acute myocardial infarction, peripheral artery disease, and valvular heart disease (Supplemental Table 6).

### Statistical analysis

Patient and tumor characteristics for each cancer were summarized. CVD prevalence (identified using HES and NICOR CVD diagnosis code list) by patient and tumor characteristics was also explored. We report the mean ± SD for continuous variable and count (percentage) for categorical variables. Because of the large sample size, even trivial differences are likely to have small *P* values. Thus, we focused on presenting point estimates and 95% CIs. The point estimates reported estimate the true proportion in the population, so we also report CIs as a measure of uncertainty in the point estimates. We fit logistic regression models to determine the unadjusted associations between patient and tumor characteristics and CVD hospitalization prior to cancer diagnosis, in the full cohort and by cancer site, and report ORs with 95% CIs. We also fit fully adjusted logistic regression analysis to understand the association between each variable and CVD after adjusting for the potential confounders age, sex, race, Index of Multiple Deprivation (income domain), Charlson comorbidity index, tumor-node-metastasis stage, laterality (for breast and lung), and treatment modality.

We analyzed observed CVD prevalence overall, by patient characteristics, and by cancer site. The observed prevalence is influenced by the age distribution, which varies by cancer site. We also calculated CVD prevalence directly standardized to the age and sex distribution of the 2016 English population obtained from the Office of National Statistics. This allows a fairer comparison of the impact of CVD across cancer sites and provides estimates of CVD prevalence in a cancer population with the same age and sex distribution as the general English population. Uncertainty in the prevalence estimates was displayed in the figures with 95% CIs obtained assuming a binomial distribution. To assess the CVD burden, we plotted absolute numbers of patients with CVD by cancer site and age. We also investigated the association between cancer site and CVD using logistic regression analysis adjusting for age, sex, the income domain of the Index of Multiple Deprivation, and Charlson comorbidity index. We produced an unadjusted logistic regression analysis to investigate the association between the income domain of the Index of Multiple Deprivation and CVD, cancer stage, surgery, chemotherapy, and radiotherapy with interactions between each covariate and the income domain of the Index of Multiple Deprivation.

All analyses were performed in Stata MP version 16 (StataCorp) and R version 4.0.2 (R Foundation for Statistical Computing).

## Results

**Figure 1:**
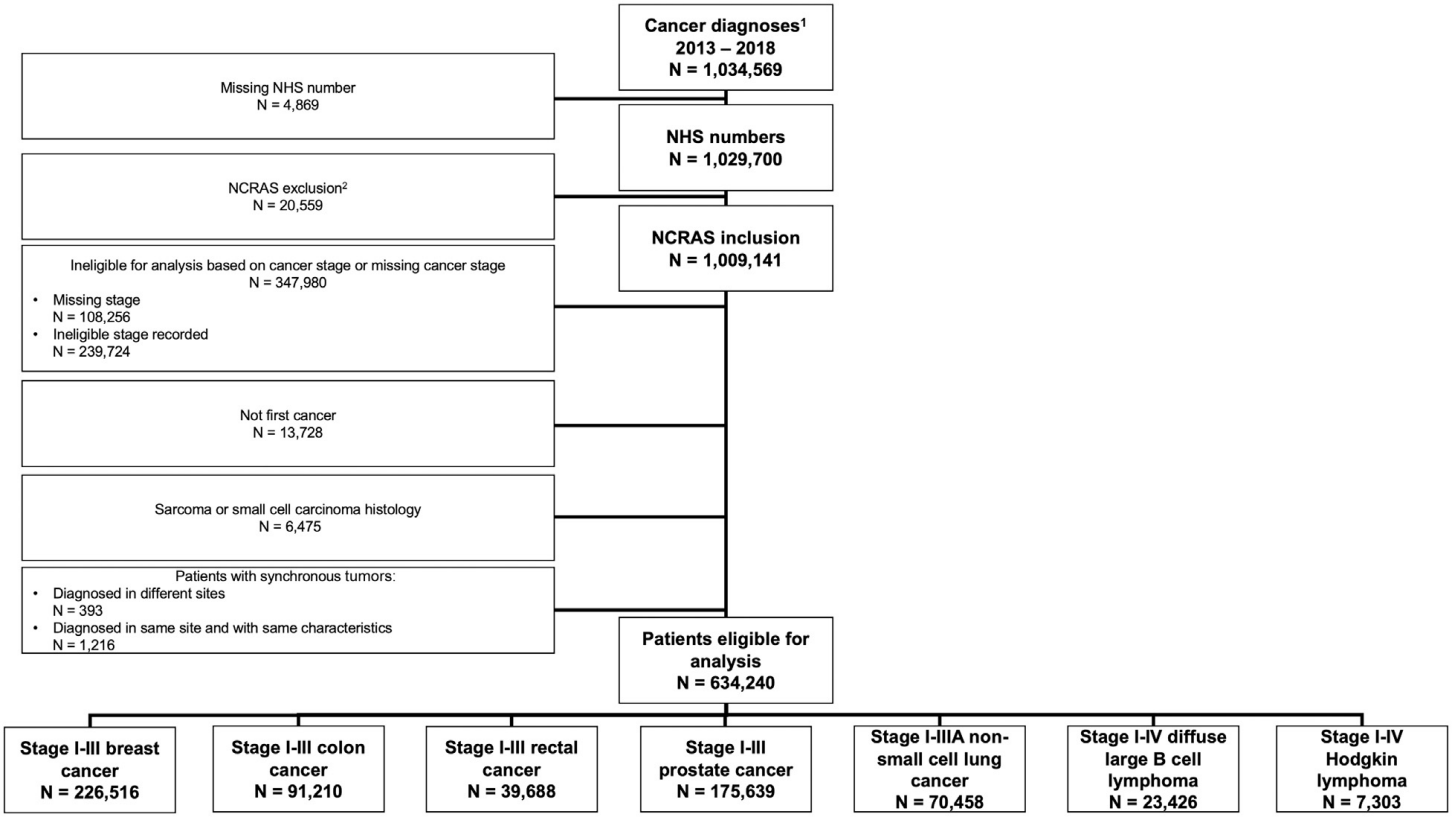
Consolidated Standards of Reporting Trials Diagram The diagram shows the selection of cancer diagnoses included in the analysis. NCRAS = National Cancer Registration and Analysis Service; NHS = National Health Service.

We extracted data from 1,034,569 cancer diagnoses in England between 2013 and 2018. After exclusions (Supplemental Table 3), 1,009,141 records remained. We excluded 347,960 tumor records on the basis of stage or missing stage, 13,728 metachronous tumor records, and 6,475 records of tumors with sarcomatous or small-cell histology (Figure 1). To analyze data at the patient level, we excluded 393 patients with 798 synchronous tumors diagnosed at ≥2 different sites and 1,216 patients with 2,454 synchronous tumors diagnosed at the same site with the same prognosis (Supplemental Table 2).

Overall, the analysis included 634,240 patients (226,516 with stage I-III breast cancer, 91,210 with stage I-III colon cancer, 39,688 with stage I-III rectal cancer, 175,639 with stage I-III prostate cancer, 70,458 with stage I-IIIA NSCLC, 23,426 with stage I-IV DLBCL, and 7,303 with stage I-IV Hodgkin lymphoma) (Figure 1).

The mean age was 67.2 ± 12.7 years, ranging from 62.5 ± 13.7) years in the breast cancer cohort to 72.9 ± 10.3) years in the NSCLC cohort. Men represented 303,021 diagnoses (47.8%), 564,687 (89.0%) had White race, 417,407 (65.8%) had the income domain of the Index of Multiple Deprivation score 1 to 3, and 295,961 (46.7%) had no Charlson comorbidity index comorbidities (excluding CVDs) recorded within 5 years before cancer diagnosis (Table 1, Supplemental Tables 7 to 13).

**Table 1:** Patient, Disease, and Tumor Characteristics in the Overall and Individual Tumor Cohorts

	Full Cohort (N = 634,240)	Breast (n = 226,516)	Colon (n = 91,210)	Rectal (n = 39,688)	Prostate (n = 175,639)	NSCLC (n = 70,458)	DLBCL (n = 23,426)	Hodgkin Lymphoma (n = 7,303)
Age at cancer diagnosis, y	67.2 ± 12.7	62.5 ± 13.9	71.2 ± 12.3	68.6 ± 12.2	69.1 ± 8.6	72.9 ± 10.3	52.5 ± 18.3	68.0 ± 13.9
Age at cancer diagnosis, y								
25-34	7,802 (1.2)	17,286 (7.6)	912 (1.0)	371 (0.9)	4 (0.0)	155 (0.2)	652 (2.8)	1,686 (23.1)
35-44	23,295 (3.7)	50,203 (22.2)	2,036 (2.2)	999 (2.5)	366 (0.2)	444 (0.6)	994 (4.2)	1,170 (16.0)
45-54	73,968 (11.7)	51,844 (22.9)	5,710 (6.3)	3,527 (8.9)	8,534 (4.9)	2,655 (3.8)	2,214 (9.5)	1,125 (15.4)
55-64	131,394 (20.7)	55,876 (24.7)	15,385 (16.9)	8,860 (22.3)	39,927 (22.7)	10,327 (14.7)	3,975 (17.0)	1,076 (14.7)
65-74	207,512 (32.7)	32,983 (14.6)	27,277 (29.9)	12,630 (31.8)	79,141 (45.1)	24,292 (34.5)	7,138 (30.5)	1,158 (15.9)
75-84	144,670 (22.8)	14,302 (6.3)	28,583 (31.3)	9,995 (25.2)	41,980 (23.9)	23,882 (33.9)	6,364 (27.2)	883 (12.1)
≥85	45,599 (7.2)	17,286 (7.6)	11,307 (12.4)	3,306 (8.3)	5,687 (3.2)	8,703 (12.4)	2,089 (8.9)	205 (2.8)
Sex								
Male	303,021 (47.8)	0 (0.0)	48,431 (53.1)	25,420 (64.0)	175,639 (100)	36,229 (51.4)	12,981 (55.4)	4,321 (59.2)
Female	331,219 (52.2)	226,516 (100)	42,779 (46.9)	14,268 (36.0)	0 (0.0)	34,229 (48.6)	10,445 (44.6)	2,982 (40.8)
Ethnicity								
White	564,687 (89.0)	198,738 (87.7)	83,317 (91.3)	36,153 (91.1)	153,282 (87.3)	66,312 (94.1)	20,921 (89.3)	5,964 (81.7)
Mixed	2,694 (0.4)	1,184 (0.5)	274 (0.3)	131 (0.3)	762 (0.4)	163 (0.2)	99 (0.4)	81 (1.1)
Asian	16,923 (2.7)	8,044 (3.6)	1,883 (2.1)	1,066 (2.7)	3,309 (1.9)	1,183 (1.7)	952 (4.1)	486 (6.7)
Black	13,579 (2.1)	4,522 (2.0)	1,339 (1.5)	418 (1.1)	6,093 (3.5)	625 (0.9)	351 (1.5)	231 (3.2)
Other	7,124 (1.1)	3,118 (1.4)	911 (1.0)	388 (1.0)	1,723 (1.0)	518 (0.7)	311 (1.3)	155 (2.1)
Missing	29,233 (4.6)	10,910 (4.8)	3,486 (3.8)	1,532 (3.9)	10,470 (6.0)	1,657 (2.4)	792 (3.4)	386 (5.3)
Income domain of the Index of Multiple Deprivation^a^								
1 (least)	140,873 (22.2)	51,814 (22.9)	20,257 (22.2)	8,776 (22.1)	43,793 (24.9)	9,891 (14.0)	5,020 (21.4)	1,322 (18.1)
2	144,911 (22.8)	52,228 (23.1)	21,337 (23.4)	9,039 (22.8)	43,060 (24.5)	12,467 (17.7)	5,317 (22.7)	1,463 (20.0)
3	131,623 (20.8)	47,406 (20.9)	18,932 (20.8)	8,361 (21.1)	36,888 (21.0)	13,612 (19.3)	4,880 (20.8)	1,544 (21.1)
4	114,231 (18.0)	40,605 (17.9)	16,392 (18.0)	7,171 (18.1)	28,899 (16.5)	15,262 (21.7)	4,371 (18.7)	1,531 (21.0)
5 (most)	102,602 (16.2)	34,463 (15.2)	14,292 (15.7)	6,341 (16.0)	22,999 (13.1)	19,226 (27.3)	3,838 (16.4)	1,443 (19.8)
Charlson comorbidity index^b^								
0	295,961 (46.7)	106,251 (46.9)	43,371 (47.6)	18,900 (47.6)	79,618 (45.3)	33,258 (47.2)	11,051 (47.2)	3,512 (48.1)
1	53,655 (8.5)	19,325 (8.5)	7,641 (8.4)	3,364 (8.5)	14,857 (8.5)	6,020 (8.5)	1,840 (7.9)	608 (8.3)
2	155,699 (24.5)	55,420 (24.5)	22,466 (24.6)	9,708 (24.5)	43,368 (24.7)	17,203 (24.4)	5,805 (24.8)	1,729 (23.7)
3	65,527 (10.3)	23,372 (10.3)	9,293 (10.2)	4,078 (10.3)	18,266 (10.4)	7,278 (10.3)	2,504 (10.7)	736 (10.1)
≥4	56,561 (8.9)	20,238 (8.9)	8,193 (9.0)	3,533 (8.9)	15,547 (8.9)	6,283 (8.9)	2,126 (9.1)	641 (8.8)
Missing^c^	6,837 (1.1)	1,910 (0.8)	246 (0.3)	105 (0.3)	3,983 (2.3)	416 (0.6)	100 (0.4)	77 (1.1)
Screen-detected								
Yes	—	99,072 (43.7)	—	—	—	—	—	—
No	—	75,931 (33.5)	—	—	—	—	—	—
Missing	—	51,513 (22.7)	—	—	—	—	—	—
TNM stage								
I	255,320 (40.3)	104,899 (46.3)	19,213 (21.1)	12,357 (31.1)	79,477 (45.3)	33,890 (48.1)	4,478 (19.1)	1,006 (13.8)
II	211,316 (33.3)	98,987 (43.7)	36,820 (40.4)	9,365 (23.6)	44,469 (25.3)	15,322 (21.7)	3,973 (17.0)	2,380 (32.6)
III	154,349 (24.3)	22,630 (10.0)	35,177 (38.6)	17,966 (45.3)	51,693 (29.4)	21,246 (30.2)	4,066 (17.4)	1,571 (21.5)
IV	13,255 (2.1)	—	—	—	—	—	10,909 (46.6)	2,346 (32.1)
Laterality								
Left		115,340 (50.9)	—	—	—	29,043 (41.2)	—	—
Right		108,849 (48.1)	—	—	—	40,480 (57.5)	—	—
Bilateral		2,219 (1.0)	—	—	—	122 (0.2)	—	—
Missing		108 (0.0)	—	—	—	813 (1.2)	—	—

Values are mean ± SD or n (% of total).

DLBCL = diffuse large B-cell lymphoma; NSCLC = non-small-cell lung cancer; TNM = tumor-node-metastasis.

a Income domain of the Index of Multiple Deprivation derived in 2015 was used for patients diagnosed with cancer in 2013, and income domain of the Index of Multiple Deprivation derived in 2019 was used for patients diagnosed with cancer after 2013.

b 5 years before diagnosis and excluding cardiovascular disease.

c Missing if not linked to Hospital Episode Statistics.

### Cardiovascular disease

Prior CVD was identified in 102,834 (16.2%) of the overall cohort (Table 2, Supplemental Table 14). Although 0.2% of CVD records were identified in NICOR only, 18,182 (17.7%) were found in both HES and NICOR datasets, with most records (84,424 [82.1%]) identified from HES only (Supplemental Figure 1). Although ischemic heart disease was the most common, many HES CVD codes were cerebrovascular, which would not feature in NICOR audits unless accompanied by other CVD diagnostic codes. Similarly, most tumors with CVD records included in an individual NICOR audit dataset were also featured in HES with a cardiovascular diagnostic code within 5 years before cancer diagnosis (Table 2, Supplemental Figure 1). Most tumors with specific CVD records were retrieved from HES.

**Table 2:** Case Ascertainment of CVD Hospitalizations Identified Using ICD-10 Code List^a^ in HES or NICOR^b^

	HES Only	HES and MINAP	HES and NACSA	HES and PCI	HES and NHFA	HES and NICOR	NICOR Only^c^	Total
Hospitalized CVD^d^	84,424	8,359	4,020	9,250	3,108	18,182	230	102,834
CVD category								
Cerebrovascular	18,584	775	473	633	479	1,782	—	20,366
Stroke	7,948	273	158	240	184	654	—	8,602
Congestive cardiac failure	15,393	2,325	1,044	1,722	3,024	6,069	—	21,462
Ischemic heart disease	48,138	8,290	3,475	9,240	1,995	16,482	—	64,620
Acute myocardial infarction	2,122	7,099	955	5,251	555	8,279	—	10,401
Peripheral artery disease	18,090	1,214	866	1,187	560	2,821	—	20,911
Valvular heart disease	12,376	1,944	2,235	1,545	1,381	5,398	—	17,775

CVD = cardiovascular disease; HES = Hospital Episode Statistics; ICD-10 = International Statistical Classification of Diseases and Related Health Problems-10th Revision; NICOR = National Initiative for Cardiovascular Outcomes Research; MINAP = Myocardial Ischaemia National Audit Project; NACSA = National Adult Cardiac Surgery Audit; PCI = percutaneous coronary intervention audit; NHFA = National Heart Failure Audit.

a ICD-10 codes for each CVD category can be found in Supplemental Table 7.

b Occurrences are reported, so rows and columns do not add up to the totals.

c CVD categories are not reported, because ICD-10 codes are not recorded in NICOR datasets.

d CVD categories are retrieved from HES ICD-10 codes and not from a NICOR dataset (ICD-10 codes are not reported in NICOR datasets).

The odds of prior CVD hospitalization increased with age, the income domain of the Index of Multiple Deprivation, and Charlson comorbidity index and were higher in men (Table 2). In the individual cancer cohorts, prevalent CVD was identified in 17,453 of 226,5162 patients in the breast cancer cohort (7.7%; 95% CI: 7.6%-7.8%), 20,161 of 91,210 in the colon cancer cohort (22.1%; 95% CI: 21.8%-22.3%), 6,699 of 39,688 in the rectal cancer cohort (16.8%; 95% CI: 16.5%-17.2%), 27,123 of 175,639 in the prostate cancer cohort (15.4%; 95% CI: 15.3%-15.6%), 25,459 of 70,458 in the NSCLC cohort (36.1%; 95% CI: 35.7%-36.4%), 5,091 of 23,426 in the DLBCL cohort (21.7%; 95% CI: 21.2%-22.2%), and 850 of 7,303 in the Hodgkin lymphoma cohort (11.6%; 95% CI: 10.8%-12.3%) (Supplemental Table 14). In the rectal cancer and NSCLC cohorts, the percentages of patients with CVD were more than 4% higher in those with stage I vs stage III disease (5.3% [95% CI: 4.4%-6.2%] and 4.3% [95% CI: 3.5%-5.1%], respectively); in Hodgkin lymphoma, CVD prevalence was 4.2% lower (95% CI: 1.6%-6.7%) (Supplemental Table 14). Prior CVD rates showed no laterality differences in the breast cancer and NSCLC cohorts.

We present the unadjusted logistic regression analysis (Supplemental Table 15) and the adjusted logistic regression analysis (Table 2) for the associations between CVD prevalence and patient- and treatment-specific characteristics in the overall and tumor-specific cohorts. We observed a significant association between increasing age, male sex, increasing income domain of the Index of Multiple Deprivation, not having surgery, radiotherapy, or chemotherapy and increasing CVD odds in the adjusted and unadjusted logistic regression analyses. In the unadjusted logistic regression analysis (Supplemental Table 15), all races had lower or similar CVD odds compared with White race. After fitting the adjusted logistic regression analysis (Table 2), Asian race had significantly higher CVD odds compared with White race in the overall cohort and in each tumor cohort, except NSCLC. In the overall cohort, the CVD odds decreased for stage II compared with stage I and increased for stages III and IV compared with stage I in both unadjusted and adjusted analyses. For rectal cancer and NSCLC, the CVD odds in stages II and III compared with stage I were lower. For breast cancer, prostate cancer, DLBCL, and Hodgkin lymphoma, we observed a significant but nonlinear association between stage and CVD odds in unadjusted logistic regression analysis, but this was no longer significant in the adjusted analysis. Charlson comorbidity index was not associated with CVD odds in the adjusted and unadjusted analysis.

An increasing income domain of the Index of Multiple Deprivation was associated with more advanced stage in the individual tumor cohorts (Supplemental Table 16). Increasing income domain of the Index of Multiple Deprivation score was associated with higher CVD rates in all tumor groups except the Hodgkin lymphoma cohort (Supplemental Tables 7 to 13). In the overall cohort, the CVD rates ranged from 13.3% for patients living in areas with income domain of the Index of Multiple Deprivation of 1 (least deprived) to 20.7% in those with an income domain of the Index of Multiple Deprivation of 5 (most deprived). CVD prevalence ranged from 6.1% to 10.1% in the breast cancer cohort, from 19.7% to 25.9% in the colon cancer cohort, from 14.9% to 20.4% in the rectal cancer cohort, from 13.5% to 18.9% in the prostate cancer cohort, from 32.6% to 38.4% in the NSCLC cohort, from 19.6% to 23.4% in the DLBCL cohort, and from 11.3% to 12.7% in the Hodgkin lymphoma cohort.

**Figure 2:**
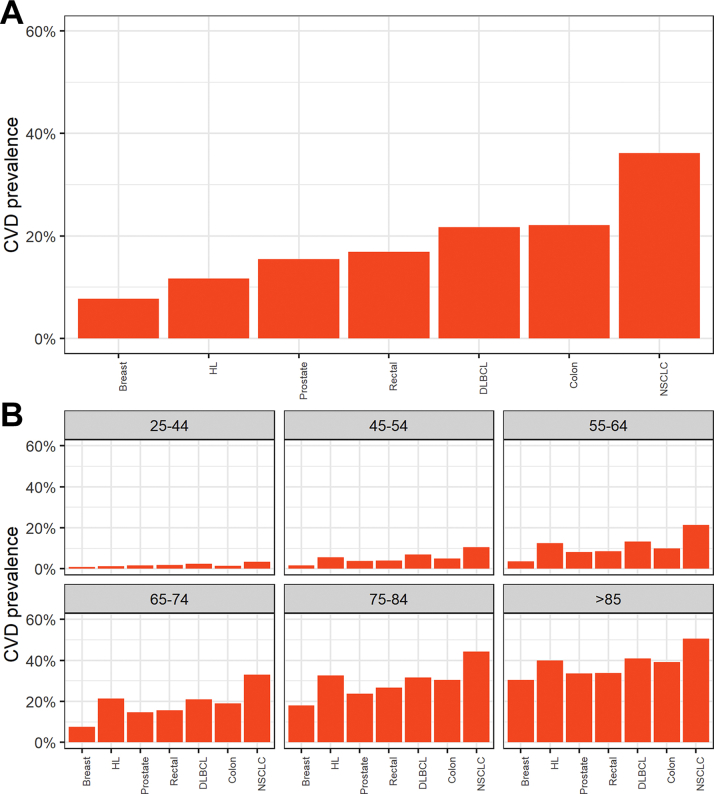
Patients With Prevalent Cardiovascular Disease According to Tumor Type and Age Patients with prevalent cardiovascular disease according to tumor type **(A)** and age **(B)**. The figure shows the proportion of patients with diagnoses of cardiovascular disease in each tumor cohort and according to age group. CVD = cardiovascular disease; DLBCL, diffuse large B-cell lymphoma; HL = Hodgkin lymphoma; NSCLC = non-small-cell lung cancer.

Supplemental Table 14 also outlines the absolute number of individuals with CVD hospitalization before cancer diagnosis across the tumor cohorts and age groups. The prostate cancer cohort had the largest burden of CVD hospitalization (n = 27,123), followed by the NSCLC cohort (n = 25,459), colon cancer cohort (n = 20,161), breast cancer cohort (n = 17,453), and rectal cancer cohort (n = 6,699). The DLBCL cohort and the Hodgkin lymphoma cohort had the lowest burden of CVD (n = 5,091 and n = 850, respectively). The highest absolute number of individuals with prior hospitalization for CVD occurred between 65 and 84 years of age in all cancer cohorts. The overall proportion of patients with CVD is shown in Figures 2A and 2B.

**Figure 3:**
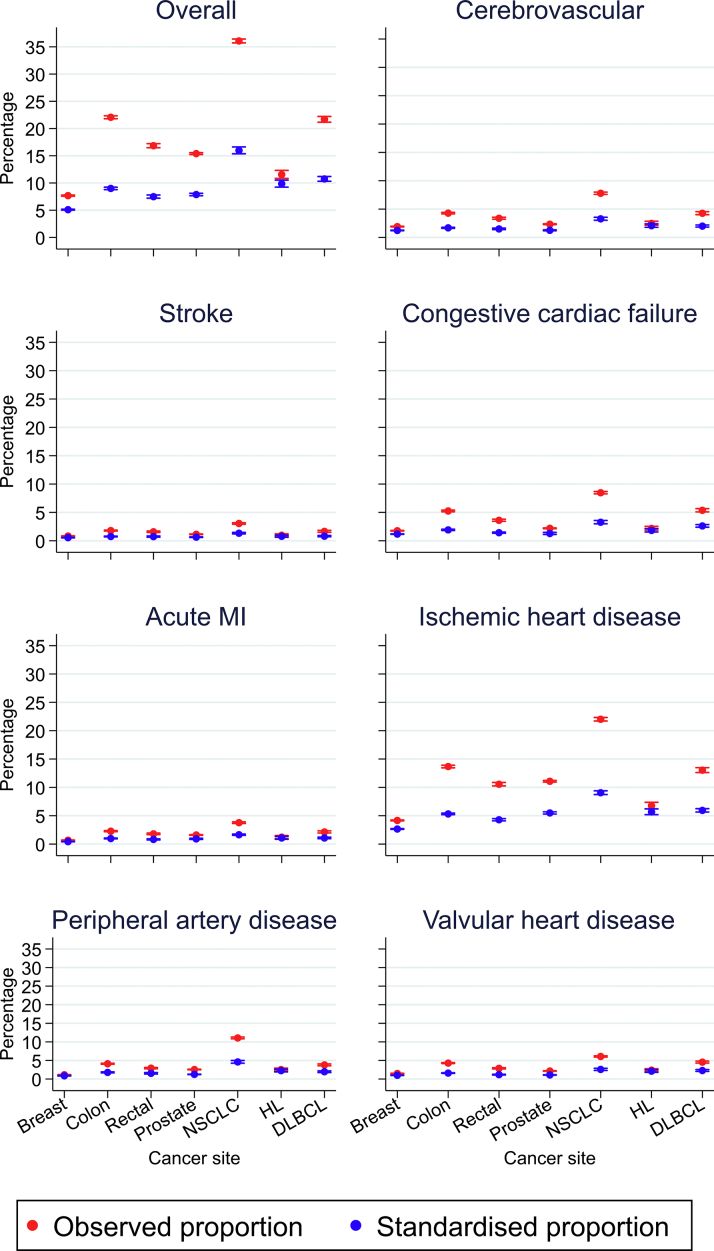
Prevalence of Cardiovascular Disease Before Cancer Diagnosis by Cancer Site The figure shows the observed and standardized prevalence of cardiovascular disease in Hospital Episode Statistics by cancer site. Prevalence is standardized by the age– and sex stratum–specific 2016 Office for National Statistics population estimates. MI = myocardial infarction; other abbreviations as in Figure 2.

The observed CVD prevalence across tumor groups is shown in Figure 3. Age- and sex-standardized CVD prevalence was much lower than the observed prevalence, as patients with cancer were older than the general population. The NSCLC cohort had a higher standardized prevalence compared with other cancer sites. The NSCLC cohort also had the highest observed prevalence of cerebrovascular disease (7.8%; 95% CI: 7.6%-8.0%), stroke (3.0%; 95% CI: 2.9%-3.2%), congestive cardiac failure (8.5%; 95% CI: 8.3%-8.6%), acute myocardial infarction (3.8%; 95% CI: 3.6%-3.9%), ischemic heart disease (22.0%; 95% CI: 21.7%-22.3%), peripheral vascular disease (11.1%; 95% CI: 10.8%-11.3%), and valvular heart disease (6.1%; 95% CI: 5.9%-6.2%). The prevalence of CVD subtypes was lowest in patients with breast cancer (cerebrovascular disease [1.9%; 95% CI: 1.9%-2.0%], stroke [0.8%; 95% CI: 0.8%-0.9%], congestive cardiac failure [1.8%; 95% CI: 1.7%-1.8%], acute myocardial infarction [0.7%; 95% CI: 0.6%-0.7%], ischemic heart disease [4.2%; 95% CI: 4.1%-4.2%], peripheral vascular disease [1.2%; 95% CI: 1.1%-1.2%], and valvular heart disease [1.5%; 95% CI: 1.5%-1.6%]).

**Figure 4:**
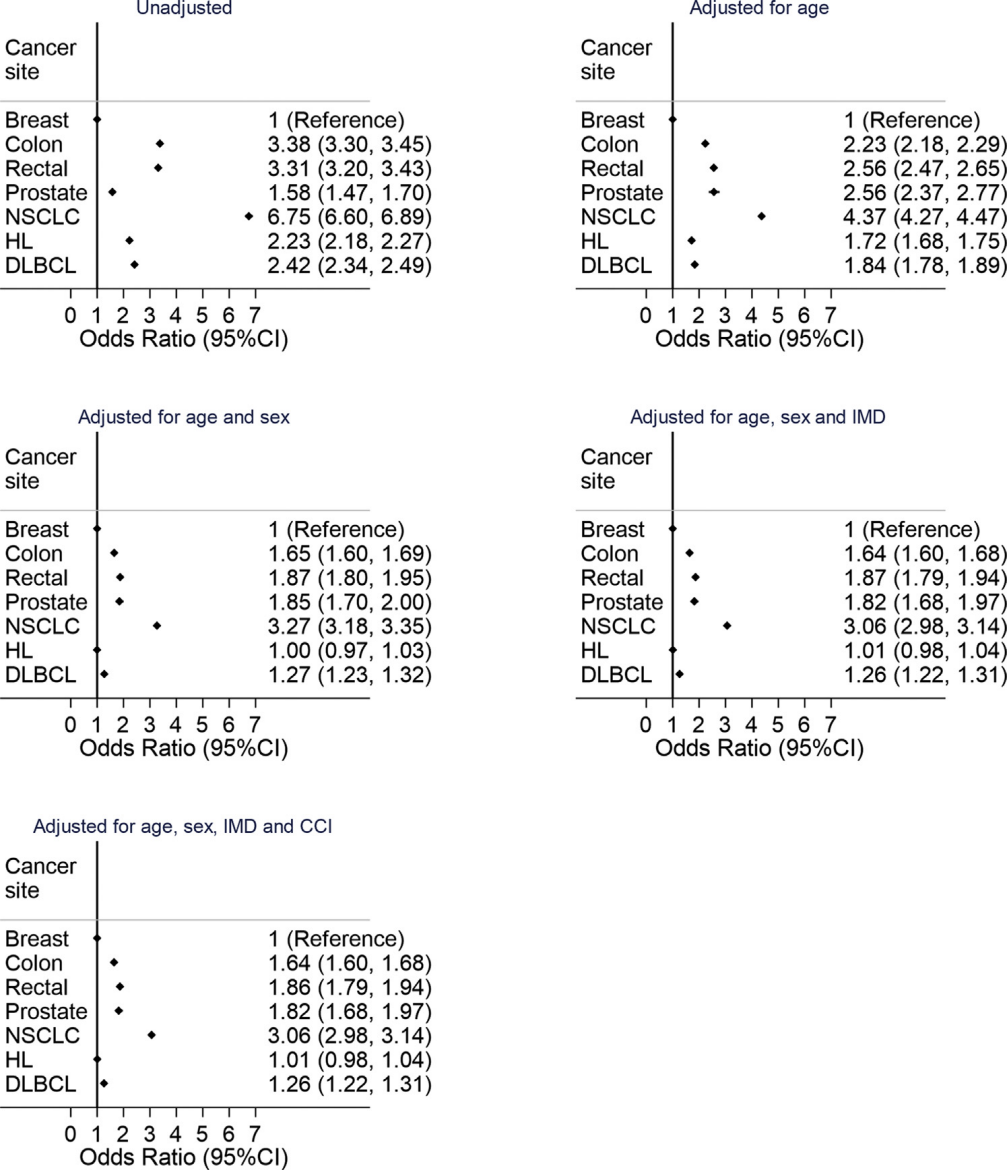
Associations Between Prevalent Cardiovascular Disease and Cancer Site The figure shows the progressively adjusted logistic regression analysis reporting odds ratios of associations between cardiovascular disease recorded in Hospital Episode Statistics and/or National Institute for Cardiovascular Outcomes Research and cancer site. The reference is the breast cancer cohort. CCI = Charlson comorbidity index; IMD = Index of Multiple Deprivation; other abbreviations as in Figure 2.

Compared with breast cancer, other cancer cohorts had significantly higher CVD prevalence, with the unadjusted OR for each cancer site compared with breast cancer >1.5 and for NSCLC an OR of 6.75 (95% CI: 6.60-6.89) (Figure 4). After adjustment for age, sex, the income domain of the Index of Multiple Deprivation, and Charlson comorbidity index, all cancer sites apart from Hodgkin lymphoma were significantly different from breast cancer but with attenuated ORs. Also, Hodgkin lymphoma was no longer significantly different compared with breast cancer after adjusting only for age and sex. CVD odds in patients with NSCLC were significantly higher than in those with breast cancer after adjustment (OR 3.06; 95% CI: 2.98-3.14).

In the overall population, compared with patients not undergoing any anticancer treatment, those receiving surgery, radiotherapy, or chemotherapy had lower odds of CVD (surgery: OR: 0.41 [95% CI: 0.41-0.42]; radiotherapy: OR: 0.50 [95% CI: 0.50-0.51]; chemotherapy: OR: 0.43 [95% CI: 0.42-0.44]) (Table 3). Patients receiving surgery, radiotherapy, or chemotherapy had lower odds of CVD compared with those not treated in most individual tumor cohorts (breast, colon, rectal, DLBCL, and Hodgkin lymphoma), but not in the prostate and NSCLC cohorts.

**Table 3:** Adjusted Odds Ratios of Cardiovascular Disease Hospitalization According to Cancer Type and Patient Characteristics^a^^,^^b^

	Full Cohort	Breast	Colon	Rectal	Prostate	NSCLC	DLBCL	Hodgkin Lymphoma
	(N = 600,057)	(n = 214,285)	(n = 87,546)	(n = 38,083)	(n = 162,175)	(n = 67,680)	(n = 22,564)	(n = 7,724)
Total with prevalent CVD	101,014	17,183	19,810	6,590	26,607	24,609	5,026	850
Age at cancer diagnosis,^c^ y								
25-54	0.18 (0.17-0.19)	0.20 (0.19-0.22)	0.19 (0.16-0.21)	0.21 (0.18-0.25)	0.26 (0.23-0.29)	0.24 (0.21-0.27)	0.20 (0.17-0.23)	0.09 (0.07-0.11)
55-64	0.50 (0.48-0.51)	0.49 (0.46-0.52)	0.49 (0.46-0.52)	0.54 (0.49-0.59)	0.55 (0.53-0.57)	0.60 (0.57-0.63)	0.56 (0.51-0.63)	0.49 (0.38-0.62)
65-74	1.00 (reference)	1.00 (reference)	1.00 (reference)	1.00 (reference)	1.00 (reference)	1.00 (reference)	1.00 (reference)	1.00 (reference)
75-84	1.90 (1.86-1.93)	1.95 (1.87-2.04)	1.71 (1.64-1.79)	1.72 (1.61-1.85)	1.60 (1.55-1.65)	1.36 (1.30-1.41)	1.68 (1.55-1.82)	1.69 (1.38-2.08)
≥85	2.78 (2.71-2.85)	2.41 (2.27-2.55)	2.31 (2.19-2.44)	2.01 (1.83-2.22)	2.42 (2.27-2.57)	1.48 (1.40-1.57)	2.27 (2.03-2.54)	2.18 (1.56-3.05)
Sex								
Male	1.00 (reference)	—	1.00 (reference)	1.00 (reference)	—	1.00 (reference)	1.00 (reference)	1.00 (reference)
Female	0.71 (0.69-0.72)	—	0.56 (0.54-0.58)	0.55 (0.52-0.59)	—	0.59 (0.57-0.61)	0.55 (0.51-0.59)	0.55 (0.46-0.65)
Race								
White	1.00 (reference)	1.00 (reference)	1.00 (reference)	1.00 (reference)	1.00 (reference)	1.00 (reference)	1.00 (reference)	1.00 (reference)
Mixed	0.78 (0.68-0.89)	1.10 (0.83-1.45)	0.90 (0.64-1.28)	1.00 (0.59-1.68)	0.84 (0.67-1.06)	0.65 (0.44-0.95)	0.82 (0.43-1.56)	0.21 (0.03-1.58)
Asian	1.21 (1.16-1.27)	1.36 (1.24-1.49)	1.18 (1.05-1.33)	1.30 (1.09-1.54)	1.47 (1.35-1.61)	0.91 (0.80-1.03)	1.52 (1.29-1.80)	1.36 (0.98-1.89)
Black	0.58 (0.54-0.61)	0.94 (0.82-1.08)	0.69 (0.59-0.81)	0.81 (0.60-1.10)	0.64 (0.59-0.70)	0.69 (0.57-0.83)	0.70 (0.49-0.99)	0.73 (0.42-1.29)
Other	0.79 (0.73-0.85)	0.80 (0.67-0.96)	0.74 (0.61-0.90)	0.65 (0.46-0.93)	0.84 (0.72-0.97)	0.94 (0.77-1.15)	1.10 (0.80-1.51)	1.14 (0.60-2.17)
Income domain of the Index of Multiple Deprivation^d^								
1 (least)	1.00 (reference)	1.00 (reference)	1.00 (reference)	1.00 (reference)	1.00 (reference)	1.00 (reference)	1.00 (reference)	1.00 (reference)
2	1.12 (1.09-1.15)	1.09 (1.04-1.15)	1.05 (1.00-1.11)	1.02 (0.94-1.12)	1.11 (1.06-1.15)	1.10 (1.04-1.17)	1.10 (0.99-1.21)	1.08 (0.84-1.41)
3	1.25 (1.22-1.28)	1.24 (1.18-1.31)	1.17 (1.12-1.24)	1.16 (1.07-1.27)	1.17 (1.13-1.22)	1.16 (1.09-1.23)	1.14 (1.03-1.26)	1.06 (0.82-1.38)
4	1.48 (1.45-1.52)	1.43 (1.35-1.51)	1.36 (1.29-1.43)	1.38 (1.26-1.51)	1.30 (1.25-1.36)	1.22 (1.16-1.30)	1.28 (1.15-1.42)	1.58 (1.22-2.04)
5 (most)	1.88 (1.83-1.92)	1.74 (1.65-1.84)	1.60 (1.52-1.69)	1.57 (1.43-1.72)	1.59 (1.52-1.66)	1.36 (1.28-1.43)	1.45 (1.30-1.62)	1.74 (1.34-2.27)
Charlson comorbidity index^e^								
0	1.00 (reference)	1.00 (reference)	1.00 (reference)	1.00 (reference)	1.00 (reference)	1.00 (reference)	1.00 (reference)	1.00 (reference)
1	0.98 (0.96-1.01)	0.99 (0.93-1.05)	0.99 (0.93-1.05)	1.01 (0.91-1.12)	0.94 (0.89-0.99)	1.04 (0.98-1.11)	1.02 (0.89-1.15)	1.14 (0.86-1.52)
2	0.98 (0.96-1.00)	0.99 (0.95-1.03)	0.93 (0.89-0.97)	1.00 (0.93-1.07)	0.97 (0.94-1.01)	1.03 (0.99-1.07)	1.02 (0.94-1.11)	1.16 (0.95-1.41)
3	0.98 (0.96-1.01)	0.99 (0.94-1.05)	1.03 (0.97-1.09)	1.00 (0.91-1.10)	0.95 (0.91-1.00)	0.98 (0.93-1.04)	0.97 (0.87-1.09)	1.04 (0.79-1.37)
≥4	1.00 (0.97-1.03)	0.96 (0.91-1.03)	1.01 (0.95-1.07)	0.96 (0.87-1.07)	1.02 (0.97-1.07)	1.01 (0.95-1.07)	1.03 (0.92-1.16)	1.27 (0.96-1.68)
TNM stage								
	1.00 (reference)	1.00 (reference)	1.00 (reference)	1.00 (reference)	1.00 (reference)	1.00 (reference)	1.00 (reference)	1.00 (reference)
II	0.95 (0.94-0.97)	1.03 (0.99-1.07)	0.95 (0.91-0.99)	0.89 (0.83-0.96)	0.97 (0.94-1.01)	0.89 (0.86-0.93)	0.92 (0.82-1.03)	1.08 (0.81-1.44)
III	1.05 (1.03-1.07)	1.04 (0.98-1.11)	1.16 (1.11-1.22)	0.86 (0.80-0.93)	1.01 (0.98-1.05)	0.80 (0.77-0.84)	1.06 (0.95-1.19)	1.21 (0.89-1.64)
IV	1.34 (1.27-1.40)	—	—	—	—	—	1.09 (0.99-1.19)	1.33 (1.00-1.78)
Laterality								
Left	—	1.00 (reference)	—	—	—	1.00 (reference)	—	—
Right	—	1.00 (0.96-1.03)	—	—	—	1.03 (0.99-1.06)	—	—
Bilateral	—	0.95 (0.83-1.09)	—	—	—	1.02 (0.69-1.51)	—	—
Treatment modality (“no” reference for each treatment type)								
Surgery	0.68 (0.66-0.69)	0.43 (0.41-0.45)	0.77 (0.73-0.82)	0.62 (0.59-0.66)	0.52 (0.49-0.54)	0.54 (0.51-0.56)	—	—
Radiotherapy	0.69 (0.68-0.70)	0.72 (0.69-0.75)	0.93 (0.84-1.04)	1.09 (1.03-1.17)	0.87 (0.85-0.90)	0.84 (0.81-0.88)	0.80 (0.74-0.86)	0.71 (0.57-0.90)
Chemotherapy	0.74 (0.73-0.76)	0.63 (0.60-0.66)	0.48 (0.46-0.51)	0.54 (0.50-0.59)	1.03 (0.96-1.10)	0.55 (0.53-0.58)	0.54 (0.50-0.59)	0.59 (0.48-0.72)

Values are OR (95% CI).

CVD = cardiovascular disease; other abbreviations as in Table 1.

a Numbers refer to tumor diagnoses (not to patients).

b Each model is adjusted for all variables listed in the table (excluding screen-detected, because a high proportion were missing). Total numbers are smaller because we excluded any patients with missing observations in the variable included in the model.

c We grouped age ranges 25 to 34, 35 to 44, and 45 to 54 because of small numbers of observations in the younger age groups for some cancer sites.

d Income domain of the Index of Multiple Deprivation derived in 2015 was used for patients diagnosed with cancer in 2013, and income domain of the Index of Multiple Deprivation derived in 2019 was used for patients diagnosed with cancer after 2013.

e 5 years before diagnosis and excluding cardiovascular disease.

## Discussion

Our study was a large-scale, population-based analysis describing CVD prevalence in individuals with potentially curable cancers. Understanding the intersection between cancer and CVD is key to informing anticancer treatment decisions, interpreting outcomes, and planning health care provision.^37^ We used linked national registry datasets of patients diagnosed with potentially curable malignancies over 6 years in England and found an overlap between cancer and CVD in 16.2% of individuals.

An analysis of English National Cancer Diagnosis Audit data linked to primary care records showed that more than three-quarters of patients with cancer had ≥1 comorbidity,^38^ with comparable standardized CVD prevalence across tumor types. Our study revealed a much higher standardized prevalence in patients with NSCLC, reflecting the high observed prevalence in this cohort and suggesting that age and sex can only partially explain the high CVD burden in this group. This difference is likely to be driven not only by the older age of individuals with NSCLC but also by risk factors shared by CVD and lung malignancies.^4^ Lifestyle factors may explain the difference in CVD prevalence among the various tumor groups, including the higher CVD rate in the NSCLC cohort. The difference between the Hodgkin lymphoma and the breast cancer cohort was no longer significant after adjusting for age and sex, which are key drivers of the CVD prevalence in this cohort. Nonetheless, these findings are relevant to better inform the provision of cardio-oncology services and allocate resources to improve outcomes for patients with cancer and a higher CVD prevalence.

Comorbidities are more common among lung cancer survivors and less frequent among breast and prostate cancer survivors.^39^ One study documented that 43.6% of patients diagnosed with potentially curable NSCLC in England from 2012 to 2016 had CVD,^36^ which affected resection and mortality rates.^21^ In the general population, older age is associated with a higher prevalence of CVD,^40^ and CVD contributes to an increasing burden of morbidity and disability in community-dwelling older individuals. Prospective trials and cancer registry analyses have documented higher risk for heart failure in patients with potentially curable malignancies and CVD and cardiovascular risk factors receiving cytotoxic or targeted therapies.^41^, ^42^, ^43^, ^44^, ^45^ Similar concerns exist for patients potentially suitable for locoregional treatments.^36^^,^^46^ Pre-existing CVD may represent a contraindication for pursuing specific anticancer treatment options or require adjustments, possibly hindering the chances of cure in individuals with potentially curable cancer. In future analyses, we plan to examine the geographic variation of CVD rates and its impact on anticancer treatments.

CVD is also an increasingly prevalent exclusion criterion for studies investigating novel anticancer treatments.^47^ This has substantial implications on limiting not only the access of patients with cancer to experimental treatments but also trial results applicability,^48^ trial design, drug development, and drug labeling.^49^

Our study confirms that men, older individuals, and those living in socioeconomically deprived areas had a higher CVD burden. These factors have important impact on the prevalence of CVD in patients with potentially curable malignancies (Figure 4). Male sex is a risk factor for higher coronary artery disease rates and mortality.^50^ Patients undergoing surgery, radiotherapy, or chemotherapy have lower odds of CVD compared with those not treated in the overall cohort and in most individual tumor cohorts. The burden of comorbidities increases with age^39^^,^^51^ and may influence overall and non-cancer-related mortality^52^, ^53^, ^54^ but also affect anticancer treatment tolerance.^55^ For patients with breast cancer, CVD may also influence tumor-specific mortality.^56^

We demonstrated an increasing prevalence of CVD associated with worse deprivation in all tumor cohorts except Hodgkin lymphoma. In this analysis, a higher score of the income domain of the Index of Multiple Deprivation, which corresponds to lower income and higher levels of deprivation, was also associated with more advanced tumor stage. Socioeconomic inequalities have a significant impact on cancer presentation, diagnosis, and treatment.^57^ Despite efforts aiming to reduce them in England, their impact on cancer survival has not substantially changed.^58^ An accurate review of care pathways for patients with cancer and comorbidities may mitigate their detrimental effect on outcomes.^59^

Our analysis suggests that CVD can be ascertained in HES, although the sensitivity and specificity of diagnostic codes from this source still need to be defined. A significant number of CVD codes were retrieved from HES, while fewer were included also in the various NICOR datasets. Despite having both NICOR and HES data focus on hospital-based diagnoses captured in the inpatient setting, NICOR includes data on procedures and HES data are derived from admission codes. As a result, these datasets include different populations. As HES was the primary source of CVD records, HES is a sensitive source of data to ascertain the CVD burden in this population. Although NICOR databases may be more specific and have better diagnostic accuracy to determine specific CVD categories and its severity, HES is a valuable source of data to elucidate the coexistence of cancer and CVD.

Our findings have relevant clinical implications. We found that pre-existing CVD is common in individuals with potentially curable cancers. Although the decreased CVD odds in patients undergoing specific anticancer treatments may be confounded by multiple factors and causality cannot be determined, CVD may influence cancer treatment decision making. Importantly, our analysis showed that CVD is not evenly distributed among cohorts of individuals with different cancers. Specifically, we have identified categories in which CVD is particularly common: older adults, those with NSCLC, and those living in deprived areas. These factors have important implications on the provision of cardio-oncology services across England, to ensure service distribution matches need. We plan to investigate the geographic CVD distribution in this population and how this relates to the availability of specialized cardio-oncology services across England.

### Study limitations

First, we did not incorporate confounders such as smoking, diet, physical activity, obesity, alcohol, and concurrent medications because they were not recorded in cancer registry datasets, although social deprivation might represent a proxy for these lifestyle confounders. Cardiovascular risk factors are captured only by the NICOR datasets; therefore, these are available only for a subset of the individuals included in this study.

Second, our analysis was focused on hospitalizations, and we did not investigate events recorded only in primary care. This increases diagnostic accuracy but does not consider the primary care CVD burden and potential gaps between primary and inpatient care. This may have led to underestimations of CVD prevalence,^60^ although HES outpatient has limited diagnosis data, and integrating NICOR data did not substantially alter our results. We did not analyze data on CVD severity, as these data are not captured in HES. We excluded patients with missing data on several variables, which resulted in a large amount of missing data, but we performed a complete case analysis, which requires a plausible missing-at-random assumption.^61^ However, the data we used in our analysis were from 2013, when recording of variables such as cancer stage in NCRD improved to minimize this potential limitation.^62^ Moreover, case ascertainment of valve disease may be poor because of inconsistent coding approaches. Additionally, the population included was not racially diverse, and these findings may not be applicable to different geographic areas. We did not investigated the impact, although this will be the primary endpoint of a subsequent study. Finally, we excluded CVD diagnosed after cancer diagnosis to avoid including conditions caused by anticancer treatments.^36^

## Conclusions

We found significant overlap between CVD and potentially curable cancer diagnoses, along with substantial differences on the basis of age, sex, socioeconomic deprivation, and tumor types (Central Illustration). A key feature of our analysis is the use of both cancer registry and CVD audit datasets to elucidate the burden of CVD in cancer cohorts alongside key variables such as comorbidities and Index of Multiple Deprivation. However, further research is needed to investigate the variation in CVD prevalence in patients with cancer. Overall, these results have important implications at 2 levels. At the patient level, for individuals diagnosed with these potentially curable malignancies, the presence of CVD may have a significant impact not only on mortality and treatment benefits and treatment tolerability but also on trial eligibility. On a population level, these findings are important to interpreting overall survival differences, treatment strategies, and outcomes existing within and among countries and to informing health care policy strategies. As part of the Virtual Cardio-Oncology Research Initiative, we plan to evaluate the impact of CVD on the management of these potentially curable malignancies.

Perspectives **COMPETENCY IN MEDICAL KNOWLEDGE:** CVD was present in 16.2% of patients with potentially curable malignancies. Male sex, age, and income deprivation were associated with increased CVD prevalence. CVD prevalence was highest for patients with NSCLC and lowest for those with breast cancer.

**TRANSLATIONAL OUTLOOK:** The overlap between cancer and CVD burden is substantial and may explain cancer treatment patterns and outcomes. Future work is focused on understanding the impact of prevalent CVD on cancer management and the relationships between geography and access to cardio-oncology resources. Understanding the intersection between cancer and CVD is key to informing anticancer treatment decisions, interpreting outcomes, and planning health care provision.

**Central Illustration:**
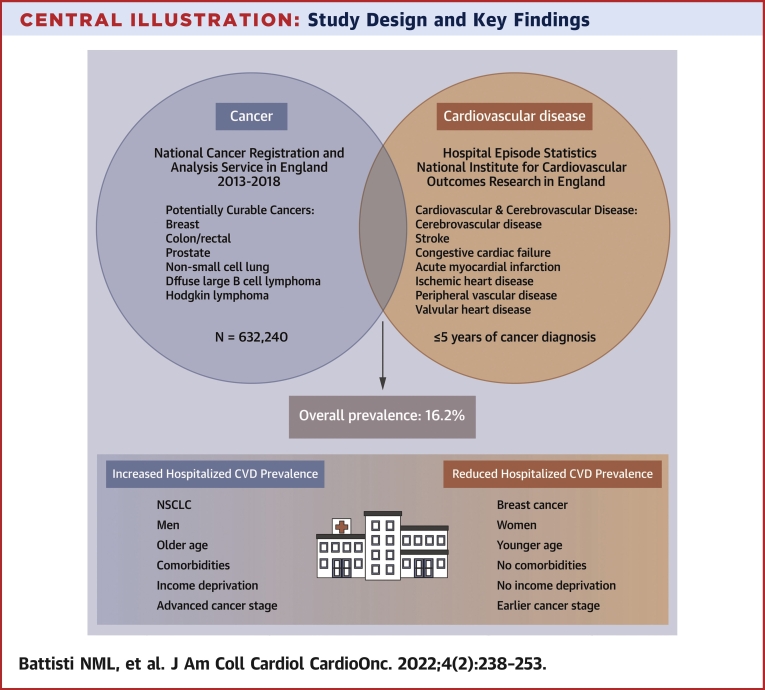
Study Design and Key Findings The figure shows the design of the analysis and the key findings. CVD = Cardiovascular Disease; NSCLC = Non-small-cell lung cancer.

## Funding Support and Author Disclosures

This study was funded by a joint research grant from the British Heart Foundation (SP/16/5/32415) and Cancer Research UK (C53325/A21134). The funders did not have any involvement in producing the report. Dr Battisti has received advisory board fees from Pfizer, Abbott and Sanofi; has received travel grants from Exact Sciences, Eli Lilly and Pfizer; and has received speaker fees from Pfizer and Abbvie. Dr Ring has received advisory board and speaker fees from Roche, Novartis, Pfizer, Merck Sharpe & Dohme, AstraZeneca, Seagen, Daiichi Sankyo, and Eli Lilly. Dr Adlam has received research funding and in-kind support from AstraZeneca for unrelated research; has received educational funding from Abbott Vascular to support a clinical research fellow; and has conducted consultancy for General Electric to support general research funds. Mr Sweeting is a full-time employee of AstraZeneca. All other authors have reported that they have no relationships relevant to the contents of this paper to disclose.
