# Supplementary material for: Prevalence of Cardiovascular Disease in Patients With Potentially Curable Malignancies: A National Registry Dataset Analysis
Source: JACC CardioOncol. 2022 Jun 21;4(2):238–53. doi: 10.1016/j.jaccao.2022.03.004 (PMC9270631; doi:10.1016/j.jaccao.2022.03.004)
Supplement: Supplemental Data [file mmc1.docx]

**SUPPLEMENTAL MATERIAL**

**Acknowledgments**

The VICORI collaborative including David Adlam, Mick Peake, John Deanfield, Lucy Elliss-Brookes, Mark de Belder, Briana Coles, Sarah Darby, Chris Gale, Mike Hawkins, Paul Lambert, Alexander Lyon, Clare Oliver-Williams, Jem Rashbass, Raoul Reulen, Alistair Ring, Mark Rutherford, Michael Sweeting, Lucy Teece, Adam Timmis, Sally Vernon, Catherine Welch and our lead lay representative, Paul Charlton.

We acknowledge the support of the National Institute for Cardiovascular Outcomes Research, and the National Cancer Registration and Analysis Service, Public Health England and their staff, particularly James Chal, Akosua Donkor, Nadeem Fazal, Anil Gunesh, Andrew Harrison, Lizz Paley, Brian Shand, Sally Vernon, and the NICOR audit leads (Abbas Khushnood, Andrew Goodwin, Peter Ludman, Theresa McDonagh, Francis Murgatroyd, Clive Weston). Particular thanks to David Forman as external member for chairing the VICORI project review panel and to Paul Charlton as Patient and Public Involvement Lead for reviewing the manuscript.

We are grateful to all UK clinical, nursing, coding and audit colleagues for their diligence and time in collecting the healthcare data used in these analyses.

Nicolò Matteo Luca Battisti and Alistair Ring would like to acknowledge the support of the Cridlan Ross Smith Charitable Trust and the NIHR Biomedical Research Centre at The Royal Marsden NHS Foundation Trust and the Institute of Cancer Research, London.

This project involves data that have been provided by or derived from patients and collected by the NHS as part of their care and support. The data are collated, maintained and quality assured by the National Cancer Registration and Analysis Service, which is part of Public Health England (PHE) and data have also been provided by the Healthcare Quality Improvement Partnership from the National Cardiac Audit Programme, part of the National Clinical Audit and Patient Outcomes Programme, which they commission. Access to the data was facilitated by the PHE Office for Data Release.

**Supplementary Table 1: Description of databases included in analysis.***

| **Database** | **Description** |
| --- | --- |
| **National Cancer Registration Dataset^1^** | Population-based cancer registry for England. It collects, quality assures and analyses data on all people living in England who are diagnosed with malignant and pre-malignant neoplasms. The dataset provides near real-time, cost-effective, comprehensive data collection and quality assurance over the entire cancer care pathway. |
| **Systemic Anti-cancer therapy^2^** | Population-based resource of anti-cancer therapy activity reported routinely by National Health Service (NHS) trusts in England. Data are collected on the treatments of patients, delivered in secondary and tertiary settings, with the intention of increasing survival, delaying further cancer progression. Does not include supportive therapies or disease-modifying intent. |
| **Radiotherapy Dataset^3^** | All NHS Acute Trust which provide radiotherapy services in England collect and submit standardised data monthly against a nationally defined data set. The purpose is to collect consistent and comparable data across all English providers of radiotherapy or private facilities where delivery is funded by the NHS, to produce a timely and definitive analytical resource of radiotherapy services across England. |
| **Hospital Episode statistics Admitted Patient Care^4^** | Provides detailed clinical, demographic and organizational information for all patients admitted to hospital. The dataset contains data on International Classification of Diseases (ICD)-10 diagnoses, procedures, dates of admission, operations and discharge, admission method (e.g. emergency or planned), care provider and many geographical variables mapped from a patient’s postcode. Data collection reimbursed by NHS which means the data are not collected for research purposes. |
| **National Institute for Cardiovascular Outcomes Research databases^5^** | Manages data collection for 6 specific conditions/procedures. The audits included in this study are shown in italics below. Clinical information about cardiovascular patients is collected by hospitals across the UK. Audits and registries were set up with clinical input and backing from specialist cardiovascular societies to ensure that the audits and analyses are clinically relevant. |
| ***Myocardial Ischaemia National Audit Project^6^*** | Collects information on patients admitted to hospital with suspected acute coronary syndromes. Data are collected in cardiology units with an emphasis on type 1 AMI and analysed to illustrate the ‘patient journey’ from a call to the emergency services or their self-presentation at an Emergency Department, through diagnosis and treatment at hospital, to the prescription of preventive medications on discharge. |
| ***National Adult Cardiac Surgery Audit^7^*** | Collects data on all major heart operations carried out in NHS hospitals throughout the UK. Includes all procedures performed that involve the heart or structures attached to the heart. For the purposes of the Audit these operations involve surgically opening the chest wall and usually the pericardium (the sac around the heart). Procedures on the heart performed with catheters (tubes inserted via arteries or veins to access the heart) are not included. |
| ***National Adult Percutaneous Coronary Intervention^8^*** | Collects data on patients who undergo a procedure to improve blood flow if symptoms due to obstructions in the heart arteries, that supply the heart muscle with blood, cannot be controlled by medical treatment. Focused on all percutaneous coronary intervention techniques (also referred to as ‘angioplasty’). |
| ***National Heart Failure Audit^9^*** | Collects data on patients admitted to hospital with acute heart failure either due to congenital heart muscle abnormalities (‘cardiomyopathies’), inflammation of the heart (‘myocarditis’) or damage associated with problems arising from coronary artery or valve disease. |

*The table explains what data is collected by each dataset to help understand differences in CVD recording.

**Supplementary Table 2– Algorithm for the selection of synchronous tumours with worse prognosis diagnosed in the same site.**

| **Breast cancer** | **Colon cancer** | **Rectal cancer** | **Prostate cancer** | **Non-small cell lung cancer** | **Diffuse large B-cell lymphoma** | **Hodgkin lymphoma** |
| --- | --- | --- | --- | --- | --- | --- |
| - Stage: III > II > I - HER2: positive > negative - ER: negative > positive - Grade: Undifferentiated / anaplastic > Poorly differentiated > Moderately differentiated > well differentiated - PR: negative > positive | - Stage: III > II > I - Grade: Undifferentiated / anaplastic > Poorly differentiated > Moderately differentiated > well differentiated | - Stage: III > II > I - Grade: Undifferentiated / anaplastic > Poorly differentiated > Moderately differentiated > well differentiated | - Stage: III > II > I - Gleason: group 5 > 4 > 3 > 1 > 1 - Grade: Undifferentiated / anaplastic > Poorly differentiated > Moderately differentiated > Well differentiated | - Stage: III > II > I - Grade: Undifferentiated / anaplastic > Poorly differentiated > Moderately differentiated > well differentiated | - Stage: III > II > I - Grade: Undifferentiated / anaplastic > Poorly differentiated > Moderately differentiated > well differentiated | - Stage: III > II > I - Grade: Undifferentiated / anaplastic > Poorly differentiated > Moderately differentiated > well differentiated |

*Abbreviations: HER2: human epidermal growth factor receptor 2; ER: oestrogen receptor; PR: progesterone receptor.*

**Supplementary Table 3 – Eligibility criteria for the analysis.**

| **Inclusion criteria** | **Exclusion criteria** |
| --- | --- |
| Cancer diagnosis in the period 2013-2018  Cancer type and stage:   - Stage I-III breast cancer (ICD-10 code C50) - Stage I-III colon/rectal cancer (ICD-10 codes C18, C19, C20) - Stage I-III prostate cancer (ICD-10 code C61) - Stage I-IIIA non small-cell lung cancer (ICD-10 C33-C34 with ICD-O-2 morphology not in list 8041, 8042, 8043, 8044, 8045) - Stage I-IV diffuse large B-cell lymphoma (ICD-10 C83.3) - Stage I-IV Hodgkin lymphoma (ICD-10 C81)   Lived in England  Finalised cancer registration | Age <18 years at diagnosis  Missing NHS number  All behaviour codes except malignant  Age >100 years at diagnosis  Missing mortality status  Death/censoring date before diagnosis date  Carcinoid morphology  Stages 3B or 4 at diagnosis  Missing disease stage at diagnosis  Diagnosis from death certificate only  Men diagnosed with breast cancer  Females diagnosed with prostate cancer  Duplicates |

*Abbreviations: ICD-10: International Statistical Classification of Diseases and Related Health Problems-10.*

**Supplementary Table 4 – Calculation of the Charlson Comorbidity Index with and without inclusion of cardiovascular diseases.**

| **Charlson Group** | **Description** | **Charlson Score** | **Notes** |
| --- | --- | --- | --- |
| 1 | Acute myocardial infarction | 1 | Excluded from CVD-free comorbidity score |
| 2 | Congestive heart failure | 1 |  |
| 3 | Peripheral vascular disease | 1 |  |
| 4 | Cerebral vascular accident | 1 |  |
| 5 | Dementia | 1 | - |
| 6 | Pulmonary disease | 1 | - |
| 7 | Connective tissue disorder | 1 | - |
| 8 | Peptic ulcer | 1 | - |
| 9 | Diabetes | 1 | Only highest score is counted |
| 10 | Diabetes complications | 2 |  |
| 11 | Paraplegia | 2 | - |
| 12 | Renal disease | 2 | - |
| 13 | Cancer | 2 | Derived from cancer registry data rather than HES data |
| 14 | Metastatic cancer | N/A |  |
| 15 | Liver disease | 1 | Only highest score is counted |
| 16 | Severe liver disease | 3 |  |
| 17 | HIV | 6 | - |

*Abbreviations: HIV: Human Immunodeficiency Virus; N/A: not applicable; HES: Hospital Episode Statistics.*

**Supplementary Table 5 – Definition of cardiovascular disease according to International Statistical Classification of Diseases and Related Health Problems (ICD)-10 codes.**

| **ICD-10 code** | **Description** |
| --- | --- |
| **I05** | **Rheumatic mitral valve diseases** |
| **I05.0** | Mitral stenosis |
| **I05.1** | Rheumatic mitral insufficiency |
| **I05.2** | Mitral stenosis with insufficiency |
| **I05.8** | Other mitral valve diseases |
| **I05.9** | Mitral valve disease, unspecified |
| **I06** | **Rheumatic aortic valve diseases** |
| **I06.0** | Rheumatic aortic stenosis |
| **I06.1** | Rheumatic aortic insufficiency |
| **I06.2** | Rheumatic aortic stenosis with insufficiency |
| **I06.8** | Other rheumatic aortic valve diseases |
| **I06.9** | Rheumatic aortic valve disease, unspecified |
| **I08** | **Multiple valve diseases** |
| **I08.0** | Disorders of both mitral and aortic valves |
| **I08.1** | Disorders of both mitral and tricuspid valves |
| **I08.2** | Disorders of both aortic and tricuspid valves |
| **I08.3** | Combined disorders of mitral, aortic and tricuspid valves |
| **I08.8** | Other multiple valve diseases |
| **I08.9** | Multiple valve disease, unspecified |
| **I11** | **Hypertensive heart disease.** |
| **I11.0** | Hypertensive heart disease with (congestive) heart failure |
| **I13** | **Hypertensive heart and chronic kidney disease.** |
| **I13.0** | Hypertensive heart and renal disease with (congestive) heart failure |
| **I13.2** | Hypertensive heart and renal disease with both (congestive) heart failure and renal failure |
| **I20** | **Angina pectoris** |
| **I20.0** | Unstable angina |
| **I20.8** | Other forms of angina pectoris |
| **I20.9** | Angina pectoris, unspecified |
| **I21** | **Acute myocardial infarction** |
| **I21.0** | Acute transmural myocardial infarction of anterior wall |
| **I21.1** | Acute transmural myocardial infarction of inferior wall |
| **I21.2** | Acute transmural myocardial infarction of other sites |
| **I21.3** | Acute transmural myocardial infarction of unspecified site |
| **I21.4** | Acute subendocardial myocardial infarction |
| **I21.9** | Acute myocardial infarction, unspecified |
| **I22** | **Subsequent myocardial infarction** |
| **I22.0** | Subsequent myocardial infarction of anterior wall |
| **I22.1** | Subsequent myocardial infarction of inferior wall |
| **I22.8** | Other forms of acute ischaemic heart disease |
| **I22.9** | Acute ischaemic heart disease, unspecified |
| **I23** | **Certain current complications following ST elevation (STEMI) and non-ST elevation (NSTEMI) myocardial infarction (within the 28 day period)** |
| **I23.0** | Hemopericardium as current complication following acute myocardial infarction |
| **I23.1** | Atrial septal defect as current complication following acute myocardial infarction |
| **I23.2** | Ventricular septal defect as current complication following acute MI |
| **I23.3** | Rupture of cardiac wall without hemopericardium as current complication following acute myocardial infarction |
| **I23.4** | Rupture of chordae tendineae as current complication following acute myocardial infarction |
| **I23.5** | Rupture of papillary muscle as current complication following acute myocardial infarction |
| **I23.6** | Thrombosis of atrium, auricular appendage and ventricle as current complications following acute MI |
| **I23.8** | Other current complications following acute myocardial infarction |
| **I24** | **Other acute ischaemic heart diseases** |
| **I24.8** | Other forms of acute ischaemic heart disease |
| **I24.9** | Acute ischaemic heart disease, unspecified |
| **I25** | **Chronic ischaemic heart disease** |
| **I25.0** | Atherosclerotic cardiovascular disease, so described |
| **I25.1** | Atherosclerotic heart disease |
| **I25.5** | Ischaemic cardiomyopathy |
| **I25.8** | Other forms of chronic ischaemic heart disease |
| **I25.9** | Chronic ischaemic heart disease, unspecified |
| **I27** | **Other pulmonary heart diseases** |
| **I27.9** | Pulmonary heart disease, unspecified |
| **I34** | **Nonrheumatic mitral valve disorders** |
| **I34.0** | Mitral (valve) insufficiency |
| **I34.1** | Mitral (valve) prolapse |
| **I34.2** | Nonrheumatic mitral (valve) stenosis |
| **I34.8** | Other nonrheumatic mitral valve disorders |
| **I34.9** | Nonrheumatic mitral valve disorder, unspecified |
| **I35** | **Nonrheumatic aortic valve disorders** |
| **I35.0** | Aortic (valve) stenosis |
| **I35.1** | Aortic (valve) insufficiency |
| **I35.2** | Aortic (valve) stenosis with insufficiency |
| **I35.8** | Other aortic valve disorders |
| **I35.9** | Aortic valve disorder, unspecified |
| **I42** | **Cardiomyopathy** |
| **I42.0** | Dilated cardiomyopathy |
| **I42.5** | Other restrictive cardiomyopathy |
| **I42.6** | Alcoholic cardiomyopathy |
| **I42.7** | Cardiomyopathy due to drugs and other external agents |
| **I42.9** | Cardiomyopathy, unspecified |
| **I43** | **Cardiomyopathy in diseases classified elsewhere** |
| **I43.1** | Cardiomyopathy in metabolic diseases |
| **I43.8** | Cardiomyopathy in other diseases classified elsewhere |
| **I50** | **Heart failure** |
| **I50.0** | Congestive heart failure |
| **I50.1** | Left ventricular failure |
| **I50.9** | Heart failure, unspecified |
| **I60** | **Subarachnoid haemorrhage** |
| **I60.0** | Subarachnoid haemorrhage from carotid siphon and bifurcation |
| **I60.1** | Subarachnoid haemorrhage from middle cerebral artery |
| **I60.2** | Subarachnoid haemorrhage from anterior communicating artery |
| **I60.3** | Subarachnoid haemorrhage from posterior communicating artery |
| **I60.4** | Subarachnoid haemorrhage from basilar artery |
| **I60.5** | Subarachnoid haemorrhage from vertebral artery |
| **I60.6** | Subarachnoid haemorrhage from other intracranial arteries |
| **I60.7** | Subarachnoid haemorrhage from intracranial artery, unspecified |
| **I60.8** | Other subarachnoid haemorrhage |
| **I61** | **Intracerebral haemorrhage** |
| **I61.0** | Intracerebral haemorrhage in hemisphere, subcortical |
| **I61.1** | Intracerebral haemorrhage in hemisphere, cortical |
| **I61.2** | Intracerebral haemorrhage in hemisphere, unspecified |
| **I61.4** | Intracerebral haemorrhage in cerebellum |
| **I61.6** | Intracerebral haemorrhage, multiple localized |
| **I61.9** | Intracerebral haemorrhage, unspecified |
| **I62** | **Other nontraumatic intracranial haemorrhage** |
| **I62.1** | Nontraumatic extradural haemorrhage |
| **I62.9** | Intracranial haemorrhage (nontraumatic), unspecified |
| **I63** | **Cerebral infarction** |
| **I63.0** | Cerebral infarction due to thrombosis of precerebral arteries |
| **I63.1** | Cerebral infarction due to embolism of precerebral arteries |
| **I63.2** | Cerebral infarction due to unspecified occlusion or stenosis of precerebral arteries |
| **I63.3** | Cerebral infarction due to thrombosis of cerebral arteries |
| **I63.4** | Cerebral infarction due to embolism of cerebral arteries |
| **I63.5** | Cerebral infarction due to unspecified occlusion or stenosis of cerebral arteries |
| **I63.6** | Cerebral infarction due to cerebral venous thrombosis, nonpyogenic |
| **I63.8** | Other cerebral infarction |
| **I63.9** | Cerebral infarction, unspecified |
| **I64** | **Stroke, not specified as haemorrhage or infarction** |
| **I65** | **Occlusion and stenosis of precerebral arteries, not resulting in cerebral infarction.** |
| **I65.0** | Occlusion and stenosis of vertebral artery |
| **I65.1** | Occlusion and stenosis of basilar artery |
| **I65.2** | Occlusion and stenosis of carotid artery |
| **I65.3** | Occlusion and stenosis of multiple and bilateral precerebral arteries |
| **I65.8** | Occlusion and stenosis of other precerebral artery |
| **I65.9** | Occlusion and stenosis of unspecified precerebral artery |
| **I66** | **Occlusion and stenosis of cerebral arteries, not resulting in cerebral infarction.** |
| **I66.0** | Occlusion and stenosis of middle cerebral artery |
| **I66.1** | Occlusion and stenosis of anterior cerebral artery |
| **I66.2** | Occlusion and stenosis of posterior cerebral artery |
| **I66.3** | Occlusion and stenosis of cerebellar arteries |
| **I66.4** | Occlusion and stenosis of multiple and bilateral cerebral arteries |
| **I66.8** | Occlusion and stenosis of other cerebral artery |
| **I66.9** | Occlusion and stenosis of unspecified cerebral artery |
| **I67** | **Other cerebrovascular diseases** |
| **I67.0** | Dissection of cerebral arteries, nonruptured |
| **I67.1** | Cerebral aneurysm, nonruptured |
| **I67.2** | Cerebral atherosclerosis |
| **I67.8** | Other specified cerebrovascular diseases |
| **I67.9** | Cerebrovascular disease, unspecified |
| **I69** | **Sequelae of cerebrovascular disease** |
| **I69.0** | Sequelae of subarachnoid haemorrhage |
| **I69.3** | Sequelae of cerebral infarction |
| **I69.4** | Sequelae of stroke, not specified as haemorrhage or infarction |
| **I69.8** | Sequelae of other and unspecified cerebrovascular diseases |
| **I70** | **Atherosclerosis** |
| **I70.0** | Atherosclerosis of aorta |
| **I70.1** | Atherosclerosis of renal artery |
| **I70.2** | Atherosclerosis of arteries of the extremities |
| **I70.8** | Atherosclerosis of other arteries |
| **I71** | **Aortic aneurysm and dissection** |
| **I71.0** | Dissection of aorta [any part] |
| **I71.1** | Thoracic aortic aneurysm, ruptured |
| **I71.2** | Thoracic aortic aneurysm, without mention of rupture |
| **I71.3** | Abdominal aortic aneurysm, ruptured |
| **I71.4** | Abdominal aortic aneurysm, without mention of rupture |
| **I71.5** | Thoracoabdominal aortic aneurysm, ruptured |
| **I71.6** | Thoracoabdominal aortic aneurysm, without mention of rupture |
| **I71.8** | Aortic aneurysm of unspecified site, ruptured |
| **I71.9** | Aortic aneurysm of unspecified site, without mention of rupture |
| **I72** | **Other aneurysm** |
| **I72.0** | Aneurysm of carotid artery |
| **I72.1** | Aneurysm of artery of upper extremity |
| **I72.2** | Aneurysm of renal artery |
| **I72.3** | Aneurysm of iliac artery |
| **I72.5** | Aneurysm of artery of other precerebral arteries |
| **I72.6** | Aneurysm and dissection of vertebral artery |
| **I72.8** | Aneurysm of other specified arteries |
| **I72.9** | Aneurysm of unspecified site |
| **I73** | **Other peripheral vascular diseases** |
| **I73.8** | Other specified peripheral vascular diseases |
| **I73.9** | Peripheral vascular disease, unspecified |
| **I74** | **Arterial embolism and thrombosis** |
| **I74.0** | Embolism and thrombosis of abdominal aorta |
| **I74.1** | Embolism and thrombosis of other and unspecified parts of aorta |
| **I74.2** | Embolism and thrombosis of arteries of the upper extremities |
| **I74.3** | Embolism and thrombosis of arteries of the lower extremities |
| **I74.4** | Embolism and thrombosis of arteries of extremities, unspecified |
| **I74.5** | Embolism and thrombosis of iliac artery |
| **I74.8** | Embolism and thrombosis of other arteries |
| **I74.9** | Embolism and thrombosis of unspecified artery |
| **I77** | **Other disorders of arteries and arterioles** |
| **I77.3** | Arterial fibromuscular dysplasia |
| **I77.4** | Coeliac artery compression syndrome |
| **I79** | **Disorders of arteries, arterioles and capillaries in diseases classified elsewhere** |
| **I79.0** | Aneurysm of aorta in diseases classified elsewhere |
| **I79.2** | Peripheral angiopathy in diseases classified elsewhere |

*Abbreviations: ICD-10: International Statistical Classification of Diseases and Related Health Problems-10.*

**Supplementary Table 6 – Classifications of cardiovascular disease admission codes identified in Hospital Episode Statistics) prior to cancer diagnosis**

| **Source** | **Phenotype** | **ICD-10 codes** | **Total (N=102,604) n (%)** |
| --- | --- | --- | --- |
| **Welch et al. Br J Cancer.2020;123(3):471-9.** | **Cerebrovascular** | I60 - I69 (excluding I60.9, I61.3, I61.5, I61.8, I61.8, I61.9, I62.0, I67.3, I67.4, I67.6, I67.7, I68.0, I68.2, I69.1 and I69.2) | 20,366 (19.9) |
|  | **Stroke (Cerebrovascular subgroup)** | I61 - I64 (excluding I60.9, I61.3, I61.5, I61.8 and I62.0), I69.0, I69.3 and I69.4 | 8,602 (8.4) |
|  | **Congestive cardiac failure** | I11.0, I13.0, I13.2, I42.0, I42.5, I42.6, I42.7, I42.9, I43.1, I43.8, I50.0, I50.1 and I50.9 | 21,462 (20.9) |
|  | **Ischaemic heart disease** | I20.0 - I25 (excluding I20.1 and I24.1) | 64,620 (63.0) |
|  | **Acute myocardial infarction (Ischaemic heart disease subgroup)** | I21 - I23 | 10,401 (10.1) |
|  | **Peripheral artery disease** | I70 - I74 (excluding I70.9, I72.4, I73.0 and I73.1),  I77.3, I77.4, I79.0, I79.2 and I84.6 | 20,911 (20.4) |
|  | **Valvular heart disease** | I105, I106, I108, I27.9, I34, I35, I31.1, I135.2, I35.5 and I35.9 | 17,770 (17.3) |

**Supplementary Table 7 – Patient, disease and tumour characteristics in the breast cancer cohort (N=226,516).**

| **Variable** | **Category** | | | **Overall** | | **Year** | | | | | | | | | | | |
| --- | --- | --- | --- | --- | --- | --- | --- | --- | --- | --- | --- | --- | --- | --- | --- | --- | --- |
|  |  |  |  |  |  | **2013** | | **2014** | | **2015** | | **2016** | | **2017** | | **2018** | |
|  |  |  |  | **N** | **%** | **N** | **%** | **N** | **%** | **N** | **%** | **N** | **%** | **N** | **%** | **N** | **%** |
|  |  |  |  | **226,516** |  | **34,226** |  | **37,440** |  | **38,440** |  | **38,480** |  | **38,493** |  | **39,437** |  |
| **Age at cancer diagnosis (years)** | **25-34** | | | **4,022** | **1.8** | 570 | 1.7 | 656 | 1.8 | 669 | 1.7 | 682 | 1.8 | 710 | 1.8 | 735 | 1.9 |
|  | **35-44** | | | **17,286** | **7.6** | 2,782 | 8.1 | 2,854 | 7.6 | 2,945 | 7.7 | 2,984 | 7.8 | 2,917 | 7.6 | 2,804 | 7.1 |
|  | **45-54** | | | **50,203** | **22.2** | 7,889 | 23.0 | 8,404 | 22.4 | 8,571 | 22.3 | 8,589 | 22.3 | 8,479 | 22.0 | 8,271 | 21.0 |
|  | **55-64** | | | **51,844** | **22.9** | 8,053 | 23.5 | 8,388 | 22.4 | 8,588 | 22.3 | 8,850 | 23.0 | 8,900 | 23.1 | 9,065 | 23.0 |
|  | **65-74** | | | **55,876** | **24.7** | 8,270 | 24.2 | 9,117 | 24.4 | 9,487 | 24.7 | 9,524 | 24.8 | 9,510 | 24.7 | 9,968 | 25.3 |
|  | **75-84** | | | **32,983** | **14.6** | 4,782 | 14.0 | 5,646 | 15.1 | 5,694 | 14.8 | 5,381 | 14.0 | 5,467 | 14.2 | 6,013 | 15.2 |
|  | **≥85** | | | **14,302** | **6.3** | 1,880 | 5.5 | 2,375 | 6.3 | 2,486 | 6.5 | 2,470 | 6.4 | 2,510 | 6.5 | 2,581 | 6.5 |
| **Race** | **White** | | | **198,738** | **87.7** | 30,839 | 90.1 | 33,157 | 88.6 | 33,156 | 86.3 | 33,662 | 87.5 | 33,831 | 87.9 | 34,093 | 86.4 |
|  | **Mixed** | | | **1,184** | **0.5** | 179 | 0.5 | 169 | 0.5 | 189 | 0.5 | 209 | 0.5 | 229 | 0.6 | 209 | 0.5 |
|  | **Asian** | | | **8,044** | **3.6** | 1,152 | 3.4 | 1,177 | 3.1 | 1,315 | 3.4 | 1,420 | 3.7 | 1,464 | 3.8 | 1,516 | 3.8 |
|  | **Black** | | | **4,522** | **2.0** | 646 | 1.9 | 757 | 2.0 | 692 | 1.8 | 738 | 1.9 | 837 | 2.2 | 852 | 2.2 |
|  | **Other** | | | **3,118** | **1.4** | 364 | 1.1 | 423 | 1.1 | 518 | 1.3 | 543 | 1.4 | 604 | 1.6 | 666 | 1.7 |
|  | **Missing** | | | **10,910** | **4.8** | 1,046 | 3.1 | 1,757 | 4.7 | 2,570 | 6.7 | 1,908 | 5.0 | 1,528 | 4.0 | 2,101 | 5.3 |
| **Income domain of the Index of Multiple Deprivation** | **1 - Least deprived** | | | **51,814** | **22.9** | 7,867 | 23.0 | 8,639 | 23.1 | 8,815 | 22.9 | 8,834 | 23.0 | 8,612 | 22.4 | 9,047 | 22.9 |
|  | **2** | | | **52,228** | **23.1** | 7,873 | 23.0 | 8,655 | 23.1 | 8,727 | 22.7 | 8,903 | 23.1 | 8,927 | 23.2 | 9,143 | 23.2 |
|  | **3** | | | **47,406** | **20.9** | 7,111 | 20.8 | 7,779 | 20.8 | 8,097 | 21.1 | 8,091 | 21.0 | 8,024 | 20.8 | 8,304 | 21.1 |
|  | **4** | | | **40,605** | **17.9** | 6,147 | 18.0 | 6,644 | 17.7 | 6,960 | 18.1 | 6,886 | 17.9 | 7,023 | 18.2 | 6,945 | 17.6 |
|  | **5 - Most deprived** | | | **34,463** | **15.2** | 5,228 | 15.3 | 5,723 | 15.3 | 5,841 | 15.2 | 5,766 | 15.0 | 5,907 | 15.3 | 5,998 | 15.2 |
| **Charlson comorbidity index excluding CVD^1^** | | **0** | | **106,251** | **46.9** | 16,181 | 47.3 | 17,598 | 47.0 | 18,066 | 47.0 | 18,188 | 47.3 | 17,925 | 46.6 | 18,293 | 46.4 |
|  |  | **1** | | **19,325** | **8.5** | 2,920 | 8.5 | 3,249 | 8.7 | 3,154 | 8.2 | 3,301 | 8.6 | 3,279 | 8.5 | 3,422 | 8.7 |
|  |  | **2** | | **55,420** | **24.5** | 8,344 | 24.4 | 9,167 | 24.5 | 9,531 | 24.8 | 9,352 | 24.3 | 9,453 | 24.6 | 9,573 | 24.3 |
|  |  | **3** | | **23,372** | **10.3** | 3,509 | 10.3 | 3,851 | 10.3 | 3,927 | 10.2 | 3,956 | 10.3 | 4,038 | 10.5 | 4,091 | 10.4 |
|  |  | **≥4** | | **20,238** | **8.9** | 3,078 | 9.0 | 3,345 | 8.9 | 3,505 | 9.1 | 3,425 | 8.9 | 3,391 | 8.8 | 3,494 | 8.9 |
|  |  | **Missing^2^** | | **1,910** | **0.8** | 194 | 0.6 | 230 | 0.6 | 257 | 0.7 | 258 | 0.7 | 407 | 1.1 | 564 | 1.4 |
| **Screen-detected** | **Yes** | | | **75,931** | **33.5** | 11,494 | 33.6 | 12,476 | 33.3 | 12,108 | 31.5 | 12,571 | 32.7 | 13,132 | 34.1 | 14,150 | 35.9 |
|  | **No** | | | **99,072** | **43.7** | 13,570 | 39.6 | 15,614 | 41.7 | 16,210 | 42.2 | 16,163 | 42.0 | 18,009 | 46.8 | 19,506 | 49.5 |
|  | **Missing** | | | **51,513** | **22.7** | 9,162 | 26.8 | 9,350 | 25.0 | 10,122 | 26.3 | 9,746 | 25.3 | 7,352 | 19.1 | 5,781 | 14.7 |
| **TNM stage** | **I** | | | **104,899** | **46.3** | 15,911 | 46.5 | 17,584 | 47.0 | 17,713 | 46.1 | 17,774 | 46.2 | 17,627 | 45.8 | 18,290 | 46.4 |
|  | **II** | | | **98,987** | **43.7** | 14,692 | 42.9 | 16,075 | 42.9 | 16,849 | 43.8 | 16,948 | 44.0 | 17,059 | 44.3 | 17,364 | 44.0 |
|  | **III** | | | **22,630** | **10.0** | 3,623 | 10.6 | 3,781 | 10.1 | 3,878 | 10.1 | 3,758 | 9.8 | 3,807 | 9.9 | 3,783 | 9.6 |
| **Laterality** | **Left** | | | **115,340** | **50.9** | 17,314 | 50.6 | 19,060 | 50.9 | 19,832 | 51.6 | 19,477 | 50.6 | 19,676 | 51.1 | 19,981 | 50.7 |
|  | **Right** | | | **108,849** | **48.1** | 16,573 | 48.4 | 17,999 | 48.1 | 18,190 | 47.3 | 18,605 | 48.3 | 18,421 | 47.9 | 19,061 | 48.3 |
|  | **Bilateral** | | | **2,219** | **1.0** | 309 | 0.9 | 341 | 0.9 | 413 | 1.1 | 382 | 1.0 | 383 | 1.0 | 391 | 1.0 |
|  | **Missing** | | | **108** | **0.0** | 30 | 0.1 | 40 | 0.1 | 5 | 0.0 | 16 | 0.0 | 13 | 0.0 | 4 | 0.0 |
| **ER status** | **Positive** | | | **147,482** | **65.1** | 24,328 | 71.1 | 25,519 | 68.2 | 26,117 | 67.9 | 24,201 | 62.9 | 23,608 | 61.3 | 23,709 | 60.1 |
|  | **Negative** | | | **26,438** | **11.7** | 4,159 | 12.2 | 4,468 | 11.9 | 4,516 | 11.7 | 4,411 | 11.5 | 4,535 | 11.8 | 4,349 | 11.0 |
|  | **Missing** | | | **52,596** | **23.2** | 5,739 | 16.8 | 7,453 | 19.9 | 7,807 | 20.3 | 9,868 | 25.6 | 10,350 | 26.9 | 11,379 | 28.9 |
| **PR status** | **Positive** | | | **70,727** | **31.2** | 11,984 | 35.0 | 12,284 | 32.8 | 11,856 | 30.8 | 11,404 | 29.6 | 11,290 | 29.3 | 11,909 | 30.2 |
|  | **Negative** | | | **33,913** | **15.0** | 5,344 | 15.6 | 5,647 | 15.1 | 5,787 | 15.1 | 5,698 | 14.8 | 5,804 | 15.1 | 5,633 | 14.3 |
|  | **Missing** | | | **121,876** | **53.8** | 16,898 | 49.4 | 19,509 | 52.1 | 20,797 | 54.1 | 21,378 | 55.6 | 21,399 | 55.6 | 21,895 | 55.5 |
| **HER2 status** | **Positive** | | | **24,070** | **10.6** | 3,973 | 11.6 | 4,181 | 11.2 | 4,009 | 10.4 | 4,095 | 10.6 | 3,967 | 10.3 | 3,845 | 9.7 |
|  | **Negative** | | | **152,262** | **67.2** | 23,963 | 70.0 | 25,606 | 68.4 | 25,706 | 66.9 | 26,153 | 68.0 | 25,439 | 66.1 | 25,395 | 64.4 |
|  | **Borderline** | | | **14,412** | **6.4** | 687 | 2.0 | 1,139 | 3.0 | 1,881 | 4.9 | 2,706 | 7.0 | 3,553 | 9.2 | 4,446 | 11.3 |
|  | **Missing** | | | **35,772** | **15.8** | 5,603 | 16.4 | 6,514 | 17.4 | 6,844 | 17.8 | 5,526 | 14.4 | 5,534 | 14.4 | 5,751 | 14.6 |
| **Nottingham prognostic index** | **≤2.4** | | | **20,913** | **9.2** | 3,413 | 10.0 | 3,516 | 9.4 | 3,475 | 9.0 | 3,583 | 9.3 | 3,373 | 8.8 | 3,553 | 9.0 |
|  | **>2.4 but ≤3.4** | | | **47,868** | **21.1** | 7,350 | 21.5 | 7,768 | 20.7 | 7,867 | 20.5 | 8,206 | 21.3 | 8,197 | 21.3 | 8,480 | 21.5 |
|  | **>3.4 but ≤5.4** | | | **82,357** | **36.4** | 13,196 | 38.6 | 13,696 | 36.6 | 13,988 | 36.4 | 14,041 | 36.5 | 13,615 | 35.4 | 13,821 | 35.0 |
|  | **>5.4** | | | **21,497** | **9.5** | 3,699 | 10.8 | 3,619 | 9.7 | 3,692 | 9.6 | 3,716 | 9.7 | 3,385 | 8.8 | 3,386 | 8.6 |
|  | **Missing** | | | **53,881** | **23.8** | 6,568 | 19.2 | 8,841 | 23.6 | 9,418 | 24.5 | 8,934 | 23.2 | 9,923 | 25.8 | 10,197 | 25.9 |
| **Grade of differentiation** | **Well differentiated** | | | **4,150** | **1.8** | 696 | 2.0 | 904 | 2.4 | 759 | 2.0 | 627 | 1.6 | 592 | 1.5 | 572 | 1.5 |
|  | **Moderately differentiated** | | | **34,695** | **15.3** | 5,584 | 16.3 | 5,849 | 15.6 | 5,824 | 15.2 | 5,789 | 15.0 | 5,685 | 14.8 | 5,964 | 15.1 |
|  | **Poorly differentiated** | | | **119,650** | **52.8** | 17,215 | 50.3 | 19,395 | 51.8 | 20,275 | 52.7 | 20,491 | 53.3 | 20,878 | 54.2 | 21,396 | 54.3 |
|  | **Undifferentiated / anaplastic** | | | **67,146** | **29.6** | 10,593 | 31.0 | 11,142 | 29.8 | 11,444 | 29.8 | 11,441 | 29.7 | 11,184 | 29.1 | 11,342 | 28.8 |
|  | **Not appropriate or cannot be assessed** | | | **71** | **0.0** | 13 | 0.0 | 15 | 0.0 | 12 | 0.0 | 10 | 0.0 | 10 | 0.0 | 11 | 0.0 |
|  | **Missing** | | | **804** | **0.4** | 125 | 0.4 | 135 | 0.4 | 126 | 0.3 | 122 | 0.3 | 144 | 0.4 | 152 | 0.4 |
| **Histology** | | **Ductal** | | **177,519** | **78.4** | 26,919 | 78.7 | 29,464 | 78.7 | 29,919 | 77.8 | 30,155 | 78.4 | 30,145 | 78.3 | 30,917 | 78.4 |
|  |  | **Lobular** | | **28,022** | **12.4** | 4,087 | 11.9 | 4,537 | 12.1 | 4,863 | 12.7 | 4,652 | 12.1 | 4,899 | 12.7 | 4,984 | 12.6 |
|  |  | **Mixed** | | **4,899** | **2.2** | 766 | 2.2 | 802 | 2.1 | 814 | 2.1 | 884 | 2.3 | 795 | 2.1 | 838 | 2.1 |
|  |  | **Other** | | **16,076** | **7.1** | 2,454 | 7.2 | 2,637 | 7.0 | 2,844 | 7.4 | 2,789 | 7.2 | 2,654 | 6.9 | 2,698 | 6.8 |
| **Treatment modality^3^** | | | **Surgery** | **201,522** | **89.0** | 31,308 | 91.5 | 33,580 | 89.7 | 34,187 | 88.9 | 34,283 | 89.1 | 33,752 | 87.7 | 34,412 | 87.3 |
|  |  |  | **Radiotherapy** | **153,859** | **67.9** | 24,427 | 71.4 | 26,220 | 70.0 | 25,300 | 65.8 | 25,684 | 66.7 | 25,854 | 67.2 | 26,374 | 66.9 |
|  |  |  | **Chemotherapy** | **78,877** | **34.8** | 11,845 | 34.6 | 13,097 | 35.0 | 13,583 | 35.3 | 13,728 | 35.7 | 13,444 | 34.9 | 13,180 | 33.4 |

^1^Hospital Episode Statistics assessed 5 years before cancer diagnosis.

^2^Missing if not linked to Hospital Episode Statistics.

^3^All treatments identified using National Cancer Registration and Analysis Service treatment standard operating procedure (between 1 month before and 12 months after cancer diagnosis).

*Abbreviations: ER: oestrogen receptor; PR: progesterone receptor; HER2: human epidermal growth factor receptor 2.*

**Supplementary Table 8 – Patient, disease and tumour characteristics in the colon cancer cohort (N=91,210).**

| **Variable** | | | **Category** | **Overall** | | **Year** | | | | | | | | | | | |
| --- | --- | --- | --- | --- | --- | --- | --- | --- | --- | --- | --- | --- | --- | --- | --- | --- | --- |
|  |  |  |  |  |  | **2013** | | **2014** | | **2015** | | **2016** | | **2017** | | **2018** | |
|  |  |  |  | **N** | **%** | **N** | **%** | **N** | **%** | **N** | **%** | **N** | **%** | **N** | **%** | **N** | **%** |
|  |  |  |  | **91,210** |  | **13,884** |  | **14,850** |  | **15,672** |  | **15,653** |  | **15,432** |  | **15,719** |  |
| **Age at cancer diagnosis (years)** | | | **25-34** | **912** | **1.0** | 157 | 1.1 | 167 | 1.1 | 140 | 0.9 | 199 | 1.3 | 142 | 0.9 | 107 | 0.7 |
|  |  |  | **35-44** | **2,036** | **2.2** | 290 | 2.1 | 329 | 2.2 | 323 | 2.1 | 359 | 2.3 | 354 | 2.3 | 381 | 2.4 |
|  |  |  | **45-54** | **5,710** | **6.3** | 862 | 6.2 | 930 | 6.3 | 978 | 6.2 | 1,001 | 6.4 | 931 | 6.0 | 1,008 | 6.4 |
|  |  |  | **55-64** | **15,385** | **16.9** | 2,366 | 17.0 | 2,386 | 16.1 | 2,606 | 16.6 | 2,620 | 16.7 | 2,635 | 17.1 | 2,772 | 17.6 |
|  |  |  | **65-74** | **27,277** | **29.9** | 4,222 | 30.4 | 4,422 | 29.8 | 4,616 | 29.5 | 4,697 | 30.0 | 4,706 | 30.5 | 4,614 | 29.4 |
|  |  |  | **75-84** | **28,583** | **31.3** | 4,401 | 31.7 | 4,717 | 31.8 | 5,017 | 32.0 | 4,841 | 30.9 | 4,720 | 30.6 | 4,887 | 31.1 |
|  |  |  | **≥85** | **11,307** | **12.4** | 1,586 | 11.4 | 1,899 | 12.8 | 1,992 | 12.7 | 1,936 | 12.4 | 1,944 | 12.6 | 1,950 | 12.4 |
| **Sex** | | | **Male** | **48,431** | **53.1** | 7,451 | 53.7 | 7,835 | 52.8 | 8,291 | 52.9 | 8,395 | 53.6 | 8,168 | 52.9 | 8,291 | 52.7 |
|  |  |  | **Female** | **42,779** | **46.9** | 6,433 | 46.3 | 7,015 | 47.2 | 7,381 | 47.1 | 7,258 | 46.4 | 7,264 | 47.1 | 7,428 | 47.3 |
| **Race** | | **White** | | **83,317** | **91.3** | 13,002 | 93.6 | 13,695 | 92.2 | 14,188 | 90.5 | 14,233 | 90.9 | 14,073 | 91.2 | 14,126 | 89.9 |
|  |  | **Mixed** | | **274** | **0.3** | 37 | 0.3 | 45 | 0.3 | 45 | 0.3 | 45 | 0.3 | 51 | 0.3 | 51 | 0.3 |
|  |  | **Asian** | | **1,883** | **2.1** | 252 | 1.8 | 301 | 2.0 | 313 | 2.0 | 309 | 2.0 | 354 | 2.3 | 354 | 2.3 |
|  |  | **Black** | | **1,339** | **1.5** | 184 | 1.3 | 199 | 1.3 | 226 | 1.4 | 239 | 1.5 | 240 | 1.6 | 251 | 1.6 |
|  |  | **Other** | | **911** | **1.0** | 99 | 0.7 | 136 | 0.9 | 173 | 1.1 | 161 | 1.0 | 158 | 1.0 | 184 | 1.2 |
|  |  | **Missing** | | **3,486** | **3.8** | 310 | 2.2 | 474 | 3.2 | 727 | 4.6 | 666 | 4.3 | 556 | 3.6 | 753 | 4.8 |
| **Income domain of the Index of Multiple Deprivation** | | **1 - Least deprived** | | **20,257** | **22.2** | 3,021 | 21.8 | 3,293 | 22.2 | 3,495 | 22.3 | 3,478 | 22.2 | 3,471 | 22.5 | 3,499 | 22.3 |
|  |  | **2** | | **21,337** | **23.4** | 3,221 | 23.2 | 3,420 | 23.0 | 3,716 | 23.7 | 3,765 | 24.1 | 3,555 | 23.0 | 3,660 | 23.3 |
|  |  | **3** | | **18,932** | **20.8** | 2,867 | 20.6 | 3,133 | 21.1 | 3,267 | 20.8 | 3,220 | 20.6 | 3,214 | 20.8 | 3,231 | 20.6 |
|  |  | **4** | | **16,392** | **18.0** | 2,577 | 18.6 | 2,686 | 18.1 | 2,767 | 17.7 | 2,759 | 17.6 | 2,791 | 18.1 | 2,812 | 17.9 |
|  |  | **5 - Most deprived** | | **14,292** | **15.7** | 2,198 | 15.8 | 2,318 | 15.6 | 2,427 | 15.5 | 2,431 | 15.5 | 2,401 | 15.6 | 2,517 | 16.0 |
| **Charlson comorbidity index excluding CVD^1^** | | **0** | | **43,371** | **47.6** | 6,603 | 47.6 | 7,094 | 47.8 | 7,466 | 47.6 | 7,481 | 47.8 | 7,333 | 47.5 | 7,394 | 47.0 |
|  |  | **1** | | **7,641** | **8.4** | 1,131 | 8.1 | 1,263 | 8.5 | 1,321 | 8.4 | 1,315 | 8.4 | 1,278 | 8.3 | 1,333 | 8.5 |
|  |  | **2** | | **22,466** | **24.6** | 3,422 | 24.6 | 3,632 | 24.5 | 3,852 | 24.6 | 3,836 | 24.5 | 3,828 | 24.8 | 3,896 | 24.8 |
|  |  | **3** | | **9,293** | **10.2** | 1,405 | 10.1 | 1,519 | 10.2 | 1,591 | 10.2 | 1,616 | 10.3 | 1,529 | 9.9 | 1,633 | 10.4 |
|  |  | **≥4** | | **8,193** | **9.0** | 1,291 | 9.3 | 1,304 | 8.8 | 1,413 | 9.0 | 1,357 | 8.7 | 1,399 | 9.1 | 1,429 | 9.1 |
|  |  | **Missing^2^** | | **246** | **0.3** | 32 | 0.2 | 38 | 0.3 | 29 | 0.2 | 48 | 0.3 | 65 | 0.4 | 34 | 0.2 |
| **TNM stage** | | | **I** | **19,213** | **21.1** | 2,861 | 20.6 | 3,011 | 20.3 | 3,281 | 20.9 | 3,373 | 21.5 | 3,278 | 21.2 | 3,409 | 21.7 |
|  |  |  | **II** | **36,820** | **40.4** | 5,835 | 42.0 | 6,156 | 41.5 | 6,329 | 40.4 | 6,233 | 39.8 | 6,088 | 39.5 | 6,179 | 39.3 |
|  |  |  | **III** | **35,177** | **38.6** | 5,188 | 37.4 | 5,683 | 38.3 | 6,062 | 38.7 | 6,047 | 38.6 | 6,066 | 39.3 | 6,131 | 39.0 |
| **Dukes stage** | | | **A** | **11,255** | **12.3** | 2,131 | 15.3 | 2,098 | 14.1 | 2,283 | 14.6 | 2,258 | 14.4 | 1,997 | 12.9 | 488 | 3.1 |
|  |  |  | **B** | **28,605** | **31.4** | 5,393 | 38.8 | 5,585 | 37.6 | 5,696 | 36.3 | 5,559 | 35.5 | 5,088 | 33.0 | 1,284 | 8.2 |
|  |  |  | **C** | **25,150** | **27.6** | 4,507 | 32.5 | 4,838 | 32.6 | 5,021 | 32.0 | 5,107 | 32.6 | 4,600 | 29.8 | 1,077 | 6.9 |
|  |  |  | **Missing** | **26,200** | **28.7** | 1,853 | 13.3 | 2,329 | 15.7 | 2,672 | 17.0 | 2,729 | 17.4 | 3,747 | 24.3 | 12,870 | 81.9 |
| **Grade of differentiation** | **Well differentiated** | | | **9,191** | **10.1** | 1,248 | 9.0 | 1,416 | 9.5 | 1,580 | 10.1 | 1,503 | 9.6 | 1,633 | 10.6 | 1,811 | 11.5 |
|  | **Moderately differentiated** | | | **4,239** | **4.6** | 861 | 6.2 | 780 | 5.3 | 814 | 5.2 | 751 | 4.8 | 571 | 3.7 | 462 | 2.9 |
|  | **Poorly differentiated** | | | **64,414** | **70.6** | 9,689 | 69.8 | 10,469 | 70.5 | 11,051 | 70.5 | 11,101 | 70.9 | 10,948 | 70.9 | 11,156 | 71.0 |
|  | **Undifferentiated / anaplastic** | | | **13,030** | **14.3** | 2,037 | 14.7 | 2,125 | 14.3 | 2,160 | 13.8 | 2,232 | 14.3 | 2,234 | 14.5 | 2,242 | 14.3 |
|  | **Not appropriate or cannot be assessed** | | | **85** | **0.1** | 15 | 0.1 | 20 | 0.1 | 13 | 0.1 | 14 | 0.1 | 10 | 0.1 | 13 | 0.1 |
|  | **Missing** | | | **251** | **0.3** | 34 | 0.2 | 40 | 0.3 | 54 | 0.3 | 52 | 0.3 | 36 | 0.2 | 35 | 0.2 |
| **Histology** | **Adenocarcinoma** | | | **85,224** | **93.4** | 13,059 | 94.1 | 13,904 | 93.6 | 14,640 | 93.4 | 14,556 | 93.0 | 14,465 | 93.7 | 14,600 | 92.9 |
|  | **Other** | | | **5,986** | **6.6** | 825 | 5.9 | 946 | 6.4 | 1,032 | 6.6 | 1,097 | 7.0 | 967 | 6.3 | 1,119 | 7.1 |
| **Treatment modality^3^** | **Surgery** | | | **84,211** | **92.3** | 12,911 | 93.0 | 13,748 | 92.6 | 14,485 | 92.4 | 14,474 | 92.5 | 14,256 | 92.4 | 14,337 | 91.2 |
|  | **Chemotherapy** | | | **27,259** | **29.9** | 4,014 | 28.9 | 4,461 | 30.0 | 4,701 | 30.0 | 4,654 | 29.7 | 4,687 | 30.4 | 4,742 | 30.2 |

^1^Hospital Episode Statistics assessed 5 years before cancer diagnosis.

^2^Missing if not linked to Hospital Episode Statistics.

^3^All treatments identified using National Cancer Registration and Analysis Service treatment standard operating procedure (between 1 month before and 12 months after cancer diagnosis).

*Abbreviations: CVD: cardiovascular disease.*

**Supplementary Table 9 – Patient, disease and tumour characteristics in the rectal cancer cohort (N=39,688).**

| **Variable** | | | **Category** | **Overall** | | **Year** | | | | | | | | | | | |
| --- | --- | --- | --- | --- | --- | --- | --- | --- | --- | --- | --- | --- | --- | --- | --- | --- | --- |
|  |  |  |  |  |  | **2013** | | **2014** | | **2015** | | **2016** | | **2017** | | **2018** | |
|  |  |  |  | **N** | **%** | **N** | **%** | **N** | **%** | **N** | **%** | **N** | **%** | **N** | **%** | **N** | **%** |
|  |  |  |  | **39,688** | **100.0** | **6,130** | **100.0** | **6,408** | **100.0** | **6,672** | **100.0** | **6,816** | **100.0** | **6,749** | **100.0** | **6,913** |  |
| **Age at cancer diagnosis (years)** | | | **25-34** | **371** | **0.9** | 56 | 0.9 | 57 | 0.9 | 57 | 0.9 | 65 | 1.0 | 61 | 0.9 | 75 | 1.1 |
|  |  |  | **35-44** | **999** | **2.5** | 127 | 2.1 | 125 | 2.0 | 158 | 2.4 | 194 | 2.8 | 194 | 2.9 | 201 | 2.9 |
|  |  |  | **45-54** | **3,527** | **8.9** | 585 | 9.5 | 576 | 9.0 | 580 | 8.7 | 561 | 8.2 | 579 | 8.6 | 646 | 9.3 |
|  |  |  | **55-64** | **8,860** | **22.3** | 1,329 | 21.7 | 1,435 | 22.4 | 1,484 | 22.2 | 1,485 | 21.8 | 1,486 | 22.0 | 1,641 | 23.7 |
|  |  |  | **65-74** | **12,630** | **31.8** | 1,965 | 32.1 | 1,956 | 30.5 | 2,136 | 32.0 | 2,236 | 32.8 | 2,209 | 32.7 | 2,128 | 30.8 |
|  |  |  | **75-84** | **9,995** | **25.2** | 1,583 | 25.8 | 1,708 | 26.7 | 1,721 | 25.8 | 1,695 | 24.9 | 1,646 | 24.4 | 1,642 | 23.8 |
|  |  |  | **≥85** | **3,306** | **8.3** | 485 | 7.9 | 551 | 8.6 | 536 | 8.0 | 580 | 8.5 | 574 | 8.5 | 580 | 8.4 |
| **Sex** | | | **Male** | **25,420** | **64.0** | 3,949 | 64.4 | 4,049 | 63.2 | 4,228 | 63.4 | 4,394 | 64.5 | 4,357 | 64.6 | 4,443 | 64.3 |
|  |  |  | **Female** | **14,268** | **36.0** | 2,181 | 35.6 | 2,359 | 36.8 | 2,444 | 36.6 | 2,422 | 35.5 | 2,392 | 35.4 | 2,470 | 35.7 |
| **Race** | | | **White** | **36,153** | **91.1** | 5,706 | 93.1 | 5,902 | 92.1 | 6,050 | 90.7 | 6,197 | 90.9 | 6,118 | 90.7 | 6,180 | 89.4 |
|  |  |  | **Mixed** | **131** | **0.3** | 17 | 0.3 | 17 | 0.3 | 20 | 0.3 | 20 | 0.3 | 32 | 0.5 | 25 | 0.4 |
|  |  |  | **Asian** | **1,066** | **2.7** | 137 | 2.2 | 152 | 2.4 | 198 | 3.0 | 195 | 2.9 | 187 | 2.8 | 197 | 2.8 |
|  |  |  | **Black** | **418** | **1.1** | 74 | 1.2 | 62 | 1.0 | 55 | 0.8 | 76 | 1.1 | 83 | 1.2 | 68 | 1.0 |
|  |  |  | **Other** | **388** | **1.0** | 43 | 0.7 | 70 | 1.1 | 64 | 1.0 | 57 | 0.8 | 83 | 1.2 | 71 | 1.0 |
|  |  |  | **Missing** | **1,532** | **3.9** | 153 | 2.5 | 205 | 3.2 | 285 | 4.3 | 271 | 4.0 | 246 | 3.6 | 372 | 5.4 |
| **Income domain of the Index of Multiple Deprivation** | | **1 - Least deprived** | | **8,776** | **22.1** | 1,347 | 22.0 | 1,371 | 21.4 | 1,471 | 22.0 | 1,560 | 22.9 | 1,510 | 22.4 | 1,517 | 21.9 |
|  |  | **2** | | **9,039** | **22.8** | 1,389 | 22.7 | 1,501 | 23.4 | 1,524 | 22.8 | 1,517 | 22.3 | 1,540 | 22.8 | 1,568 | 22.7 |
|  |  | **3** | | **8,361** | **21.1** | 1,248 | 20.4 | 1,340 | 20.9 | 1,422 | 21.3 | 1,437 | 21.1 | 1,416 | 21.0 | 1,498 | 21.7 |
|  |  | **4** | | **7,171** | **18.1** | 1,112 | 18.1 | 1,152 | 18.0 | 1,163 | 17.4 | 1,272 | 18.7 | 1,236 | 18.3 | 1,236 | 17.9 |
|  |  | **5 - Most deprived** | | **6,341** | **16.0** | 1,034 | 16.9 | 1,044 | 16.3 | 1,092 | 16.4 | 1,030 | 15.1 | 1,047 | 15.5 | 1,094 | 15.8 |
| **Charlson comorbidity index excluding CVD^1^** | | | **0** | **18,900** | **47.6** | 2,909 | 47.5 | 3,049 | 47.6 | 3,183 | 47.7 | 3,249 | 47.7 | 3,234 | 47.9 | 3,276 | 47.4 |
|  |  |  | **1** | **3,364** | **8.5** | 523 | 8.5 | 530 | 8.3 | 535 | 8.0 | 571 | 8.4 | 564 | 8.4 | 641 | 9.3 |
|  |  |  | **2** | **9,708** | **24.5** | 1,493 | 24.4 | 1,601 | 25.0 | 1,665 | 25.0 | 1,658 | 24.3 | 1,588 | 23.5 | 1,703 | 24.6 |
|  |  |  | **3** | **4,078** | **10.3** | 649 | 10.6 | 641 | 10.0 | 681 | 10.2 | 728 | 10.7 | 708 | 10.5 | 671 | 9.7 |
|  |  |  | **≥4** | **3,533** | **8.9** | 545 | 8.9 | 571 | 8.9 | 589 | 8.8 | 593 | 8.7 | 634 | 9.4 | 601 | 8.7 |
|  |  |  | **Missing^2^** | **105** | **0.3** | 11 | 0.2 | 16 | 0.2 | 19 | 0.3 | 17 | 0.2 | 21 | 0.3 | 21 | 0.3 |
| **TNM stage** | | | **I** | **12,357** | **31.1** | 1,862 | 30.4 | 1,943 | 30.3 | 2,087 | 31.3 | 2,149 | 31.5 | 2,115 | 31.3 | 2,201 | 31.8 |
|  |  |  | **II** | **9,365** | **23.6** | 1,528 | 24.9 | 1,542 | 24.1 | 1,561 | 23.4 | 1,595 | 23.4 | 1,581 | 23.4 | 1,558 | 22.5 |
|  |  |  | **III** | **17,966** | **45.3** | 2,740 | 44.7 | 2,923 | 45.6 | 3,024 | 45.3 | 3,072 | 45.1 | 3,053 | 45.2 | 3,154 | 45.6 |
| **Dukes’ stage** | | | **A** | **7,109** | **17.9** | 1,389 | 22.7 | 1,384 | 21.6 | 1,411 | 21.1 | 1,507 | 22.1 | 1,146 | 17.0 | 272 | 3.9 |
|  |  |  | **B** | **6,745** | **17.0** | 1,325 | 21.6 | 1,293 | 20.2 | 1,360 | 20.4 | 1,345 | 19.7 | 1,156 | 17.1 | 266 | 3.8 |
|  |  |  | **C** | **8,772** | **22.1** | 1,785 | 29.1 | 1,736 | 27.1 | 1,776 | 26.6 | 1,833 | 26.9 | 1,358 | 20.1 | 284 | 4.1 |
|  |  |  | **Missing** | **17,062** | **43.0** | 1,631 | 26.6 | 1,995 | 31.1 | 2,125 | 31.8 | 2,131 | 31.3 | 3,089 | 45.8 | 6,091 | 88.1 |
| **Grade of differentiation** | **Well differentiated** | | | **5,119** | **12.9** | 820 | 13.4 | 918 | 14.3 | 801 | 12.0 | 756 | 11.1 | 914 | 13.5 | 910 | 13.2 |
|  | **Moderately differentiated** | | | **1,803** | **4.5** | 297 | 4.8 | 293 | 4.6 | 295 | 4.4 | 330 | 4.8 | 301 | 4.5 | 287 | 4.2 |
|  | **Poorly differentiated** | | | **29,196** | **73.6** | 4,463 | 72.8 | 4,612 | 72.0 | 4,983 | 74.7 | 5,092 | 74.7 | 4,935 | 73.1 | 5,111 | 73.9 |
|  | **Undifferentiated / anaplastic** | | | **3,358** | **8.5** | 516 | 8.4 | 537 | 8.4 | 545 | 8.2 | 604 | 8.9 | 573 | 8.5 | 583 | 8.4 |
|  | **Not appropriate or cannot be assessed** | | | **25** | **0.1** | 8 | 0.1 | 3 | 0.0 | 4 | 0.1 | 2 | 0.0 | 5 | 0.1 | 3 | 0.0 |
|  | **Missing** | | | **187** | **0.5** | 26 | 0.4 | 45 | 0.7 | 44 | 0.7 | 32 | 0.5 | 21 | 0.3 | 19 | 0.3 |
| **Histology** | **Adenocarcinoma** | | | **37,771** | **95.2** | 5,867 | 95.7 | 6,130 | 95.7 | 6,353 | 95.2 | 6,505 | 95.4 | 6,386 | 94.6 | 6,530 | 94.5 |
|  | **Other** | | | **1,917** | **4.8** | 263 | 4.3 | 278 | 4.3 | 319 | 4.8 | 311 | 4.6 | 363 | 5.4 | 383 | 5.5 |
| **Treatment modality^3^** | | | **Surgery** | **27,258** | **68.7** | 4,420 | 72.1 | 4,545 | 70.9 | 4,589 | 68.8 | 4,655 | 68.3 | 4,523 | 67.0 | 4,526 | 65.5 |
|  |  |  | **Radiotherapy** | **17,165** | **43.2** | 2,844 | 46.4 | 2,892 | 45.1 | 2,861 | 42.9 | 2,878 | 42.2 | 2,808 | 41.6 | 2,882 | 41.7 |
|  |  |  | **Chemotherapy** | **15,709** | **39.6** | 2,317 | 37.8 | 2,556 | 39.9 | 2,597 | 38.9 | 2,606 | 38.2 | 2,735 | 40.5 | 2,898 | 41.9 |

^1^Hospital Episode Statistics assessed 5 years before cancer diagnosis.

^2^Missing if not linked to Hospital Episode Statistics.

^3^All treatments identified using National Cancer Registration and Analysis Service treatment standard operating procedure (between 1 month before and 12 months after cancer diagnosis).

*Abbreviations: CVD: cardiovascular disease.*

**Supplementary Table 10 – Patient, disease and tumour characteristics in the prostate cancer cohort (N=175,639).**

| **Variable** | **Category** | | | | | **Overall** | | **Years** | | | | | | | | | | | |
| --- | --- | --- | --- | --- | --- | --- | --- | --- | --- | --- | --- | --- | --- | --- | --- | --- | --- | --- | --- |
|  |  |  |  |  |  |  |  | **2013** | | **2014** | | **2015** | | **2016** | | **2017** | | **2018** | |
|  |  |  |  |  |  | **N** | **%** | **N** | **%** | **N** | **%** | **N** | **%** | **N** | **%** | **N** | **%** | **N** | **%** |
|  |  |  |  |  |  | **175,639** |  | **26,502** |  | **27,554** |  | **28,576** |  | **28,443** |  | **28,617** |  | **35,947** |  |
| **Age at cancer diagnosis (years)** | **25-34** | | | | | **4** | **0.0** | 0 | 0.0 | 1 | 0.0 | 2 | 0.0 | 1 | 0.0 | 0 | 0.0 | 0 | 0.0 |
|  | **35-44** | | | | | **366** | **0.2** | 49 | 0.2 | 53 | 0.2 | 66 | 0.2 | 63 | 0.2 | 62 | 0.2 | 73 | 0.2 |
|  | **45-54** | | | | | **8,534** | **4.9** | 1,135 | 4.3 | 1,299 | 4.7 | 1,349 | 4.7 | 1,442 | 5.1 | 1,491 | 5.2 | 1,818 | 5.1 |
|  | **55-64** | | | | | **39,927** | **22.7** | 5,968 | 22.5 | 6,208 | 22.5 | 6,405 | 22.4 | 6,236 | 21.9 | 6,600 | 23.1 | 8,510 | 23.7 |
|  | **65-74** | | | | | **79,141** | **45.1** | 11,829 | 44.6 | 12,066 | 43.8 | 13,007 | 45.5 | 12,918 | 45.4 | 12,970 | 45.3 | 16,351 | 45.5 |
|  | **75-84** | | | | | **41,980** | **23.9** | 6,572 | 24.8 | 6,877 | 25.0 | 6,816 | 23.9 | 6,905 | 24.3 | 6,610 | 23.1 | 8,200 | 22.8 |
|  | **≥85** | | | | | **5,687** | **3.2** | 949 | 3.6 | 1,050 | 3.8 | 931 | 3.3 | 878 | 3.1 | 884 | 3.1 | 995 | 2.8 |
| **Race** | | | | **White** | | **153,282** | **87.3** | 24,042 | 90.7 | 24,406 | 88.6 | 24,413 | 85.4 | 24,553 | 86.3 | 25,080 | 87.6 | 30,788 | 85.6 |
|  |  |  |  | **Mixed** | | **762** | **0.4** | 114 | 0.4 | 102 | 0.4 | 113 | 0.4 | 134 | 0.5 | 141 | 0.5 | 158 | 0.4 |
|  |  |  |  | **Asian** | | **3,309** | **1.9** | 436 | 1.6 | 520 | 1.9 | 542 | 1.9 | 550 | 1.9 | 572 | 2.0 | 689 | 1.9 |
|  |  |  |  | **Black** | | **6,093** | **3.5** | 845 | 3.2 | 889 | 3.2 | 973 | 3.4 | 949 | 3.3 | 1,147 | 4.0 | 1,290 | 3.6 |
|  |  |  |  | **Other** | | **1,723** | **1.0** | 201 | 0.8 | 234 | 0.8 | 280 | 1.0 | 297 | 1.0 | 293 | 1.0 | 418 | 1.2 |
|  |  |  |  | **Missing** | | **10,470** | **6.0** | 864 | 3.3 | 1,403 | 5.1 | 2,255 | 7.9 | 1,960 | 6.9 | 1,384 | 4.8 | 2,604 | 7.2 |
| **Income domain of the Index of Multiple Deprivation** | | | | **1 - Least deprived** | | **43,793** | **24.9** | 6,600 | 24.9 | 6,736 | 24.4 | 7,024 | 24.6 | 7,052 | 24.8 | 7,162 | 25.0 | 9,219 | 25.6 |
|  |  |  |  | **2** | | **43,060** | **24.5** | 6,499 | 24.5 | 6,705 | 24.3 | 7,016 | 24.6 | 6,937 | 24.4 | 7,014 | 24.5 | 8,889 | 24.7 |
|  |  |  |  | **3** | | **36,888** | **21.0** | 5,533 | 20.9 | 5,899 | 21.4 | 6,028 | 21.1 | 5,969 | 21.0 | 5,987 | 20.9 | 7,472 | 20.8 |
|  |  |  |  | **4** | | **28,899** | **16.5** | 4,284 | 16.2 | 4,548 | 16.5 | 4,680 | 16.4 | 4,683 | 16.5 | 4,797 | 16.8 | 5,907 | 16.4 |
|  |  |  |  | **5 - Most deprived** | | **22,999** | **13.1** | 3,586 | 13.5 | 3,666 | 13.3 | 3,828 | 13.4 | 3,802 | 13.4 | 3,657 | 12.8 | 4,460 | 12.4 |
| **Charlson comorbidity index excluding CVD^1^** | | | | **0** | | **79,618** | **45.3** | 12,433 | 46.9 | 12,695 | 46.1 | 13,048 | 45.7 | 13,013 | 45.8 | 12,762 | 44.6 | 15,667 | 43.6 |
|  |  |  |  | **1** | | **14,857** | **8.5** | 2,277 | 8.6 | 2,285 | 8.3 | 2,510 | 8.8 | 2,432 | 8.6 | 2,409 | 8.4 | 2,944 | 8.2 |
|  |  |  |  | **2** | | **43,368** | **24.7** | 6,443 | 24.3 | 6,817 | 24.7 | 7,095 | 24.8 | 7,113 | 25.0 | 7,049 | 24.6 | 8,851 | 24.6 |
|  |  |  |  | **3** | | **18,266** | **10.4** | 2,723 | 10.3 | 2,943 | 10.7 | 2,893 | 10.1 | 2,902 | 10.2 | 2,952 | 10.3 | 3,853 | 10.7 |
|  |  |  |  | **≥4** | | **15,547** | **8.9** | 2,294 | 8.7 | 2,446 | 8.9 | 2,580 | 9.0 | 2,451 | 8.6 | 2,624 | 9.2 | 3,152 | 8.8 |
|  |  |  |  | **Missing^2^** | | **3,983** | **2.3** | 332 | 1.3 | 368 | 1.3 | 450 | 1.6 | 532 | 1.9 | 821 | 2.9 | 1,480 | 4.1 |
| **TNM stage** | **I** | | | | | **79,477** | **45.3** | 11,868 | 44.8 | 11,989 | 43.5 | 12,530 | 43.8 | 12,223 | 43.0 | 13,364 | 46.7 | 17,503 | 48.7 |
|  | **II** | | | | | **44,469** | **25.3** | 7,758 | 29.3 | 7,985 | 29.0 | 8,078 | 28.3 | 7,662 | 26.9 | 6,284 | 22.0 | 6,702 | 18.6 |
|  | **III** | | | | | **51,693** | **29.4** | 6,876 | 25.9 | 7,580 | 27.5 | 7,968 | 27.9 | 8,558 | 30.1 | 8,969 | 31.3 | 11,742 | 32.7 |
| **Gleason score** | **Group 1 (3+3)** | | | | | **42,774** | **24.4** | 8,254 | 31.1 | 7,935 | 28.8 | 6,666 | 23.3 | 6,292 | 22.1 | 6,220 | 21.7 | 7,407 | 20.6 |
|  | **Group 2 (3+4)** | | | | | **57,810** | **32.9** | 7,925 | 29.9 | 8,614 | 31.3 | 9,684 | 33.9 | 9,755 | 34.3 | 9,586 | 33.5 | 12,246 | 34.1 |
|  | **Group 3 (4+3)** | | | | | **29,644** | **16.9** | 3,983 | 15.0 | 4,207 | 15.3 | 4,928 | 17.2 | 4,862 | 17.1 | 5,129 | 17.9 | 6,535 | 18.2 |
|  | **Group 4 (4+4, 3+5, 5+3)** | | | | | **14,966** | **8.5** | 2,107 | 8.0 | 2,161 | 7.8 | 2,413 | 8.4 | 2,550 | 9.0 | 2,625 | 9.2 | 3,110 | 8.7 |
|  | **Group 5 (4+5, 5+4, 5+5)** | | | | | **18,623** | **10.6** | 2,513 | 9.5 | 2,622 | 9.5 | 2,953 | 10.3 | 3,215 | 11.3 | 3,196 | 11.2 | 4,124 | 11.5 |
|  | **Missing** | | | | | **11,822** | **6.7** | 1,720 | 6.5 | 2,015 | 7.3 | 1,932 | 6.8 | 1,769 | 6.2 | 1,861 | 6.5 | 2,525 | 7.0 |
| **Grade of differentiation** | | | **Well differentiated** | | | **116,616** | **66.4** | 1,646 | 6.2 | 5,073 | 18.4 | 23,686 | 82.9 | 24,459 | 86.0 | 26,736 | 93.4 | 35,016 | 97.4 |
|  |  |  | **Moderately differentiated** | | | **1,516** | **0.9** | 22 | 0.1 | 49 | 0.2 | 504 | 1.8 | 551 | 1.9 | 254 | 0.9 | 136 | 0.4 |
|  |  |  | **Poorly differentiated** | | | **20,965** | **11.9** | 8,282 | 31.3 | 7,222 | 26.2 | 2,421 | 8.5 | 1,916 | 6.7 | 814 | 2.8 | 310 | 0.9 |
|  |  |  | **Undifferentiated / anaplastic** | | | **31,063** | **17.7** | 14,041 | 53.0 | 12,932 | 46.9 | 1,738 | 6.1 | 1,381 | 4.9 | 691 | 2.4 | 280 | 0.8 |
|  |  |  | **Not appropriate or cannot be assessed** | | | **5,134** | **2.9** | 2,506 | 9.5 | 2,270 | 8.2 | 168 | 0.6 | 78 | 0.3 | 61 | 0.2 | 51 | 0.1 |
|  |  |  | **Missing** | | | **345** | **0.2** | 5 | 0.0 | 8 | 0.0 | 59 | 0.2 | 58 | 0.2 | 61 | 0.2 | 154 | 0.4 |
| **Histology** | | **Adenocarcinoma** | | | | **170,221** | **96.9** | 25,953 | 97.9 | 26,873 | 97.5 | 27,813 | 97.3 | 27,480 | 96.6 | 27,515 | 96.1 | 34,587 | 96.2 |
|  |  | **Other** | | | | **5,418** | **3.1** | 549 | 2.1 | 681 | 2.5 | 763 | 2.7 | 963 | 3.4 | 1,102 | 3.9 | 1,360 | 3.8 |
| **Treatment modality^3^** | | | | | **Surgery** | **37,676** | **21.5** | 4,979 | 18.8 | 5,456 | 19.8 | 6,224 | 21.8 | 6,214 | 21.8 | 6,516 | 22.8 | 8,287 | 23.1 |
|  |  |  |  |  | **Radiotherapy** | **62,070** | **35.3** | 9,784 | 36.9 | 9,879 | 35.9 | 9,373 | 32.8 | 9,833 | 34.6 | 9,884 | 34.5 | 13,317 | 37.0 |
|  |  |  |  |  | **Chemotherapy** | **7,039** | **4.0** | 222 | 0.8 | 885 | 3.2 | 1,442 | 5.0 | 1,423 | 5.0 | 1,412 | 4.9 | 1,655 | 4.6 |

^1^Hospital Episode Statistics assessed 5 years before cancer diagnosis.

^2^Missing if not linked to Hospital Episode Statistics.

^3^All treatments identified using National Cancer Registration and Analysis Service treatment standard operating procedure (between 1 month before and 12 months after cancer diagnosis).

*Abbreviations: CVD: cardiovascular disease.*

**Supplementary Table 11 – Patient, disease and tumour characteristics in the non-small-cell lung cancer cohort (N=70,458).**

| **Variable** | | | **Category** | | | **Overall** | | **Years** | | | | | | | | | | | |
| --- | --- | --- | --- | --- | --- | --- | --- | --- | --- | --- | --- | --- | --- | --- | --- | --- | --- | --- | --- |
|  |  |  |  |  |  |  |  | **2013** | | **2014** | | **2015** | | **2016** | | **2017** | | **2018** | |
|  |  |  |  |  |  | **N** | **%** | **N** | **%** | **N** | **%** | **N** | **%** | **N** | **%** | **N** | **%** | **N** | **%** |
|  |  |  |  |  |  | **70,458** |  | **10,745** |  | **11,780** |  | **11,931** |  | **12,128** |  | **12,444** |  | **11,430** |  |
| **Age at cancer diagnosis (years)** | | | **25-34** | | | **155** | **0.2** | 21 | 0.2 | 23 | 0.2 | 29 | 0.2 | 27 | 0.2 | 28 | 0.2 | 27 | 0.2 |
|  |  |  | **35-44** | | | **444** | **0.6** | 65 | 0.6 | 67 | 0.6 | 83 | 0.7 | 77 | 0.6 | 71 | 0.6 | 81 | 0.7 |
|  |  |  | **45-54** | | | **2,655** | **3.8** | 406 | 3.8 | 483 | 4.1 | 466 | 3.9 | 437 | 3.6 | 445 | 3.6 | 418 | 3.7 |
|  |  |  | **55-64** | | | **10,327** | **14.7** | 1,678 | 15.6 | 1,749 | 14.8 | 1,866 | 15.6 | 1,706 | 14.1 | 1,777 | 14.3 | 1,551 | 13.6 |
|  |  |  | **65-74** | | | **24,292** | **34.5** | 3,745 | 34.9 | 4,045 | 34.3 | 4,042 | 33.9 | 4,230 | 34.9 | 4,267 | 34.3 | 3,963 | 34.7 |
|  |  |  | **75-84** | | | **23,882** | **33.9** | 3,607 | 33.6 | 3,954 | 33.6 | 3,975 | 33.3 | 4,159 | 34.3 | 4,252 | 34.2 | 3,935 | 34.4 |
|  |  |  | **≥85** | | | **8,703** | **12.4** | 1,223 | 11.4 | 1,459 | 12.4 | 1,470 | 12.3 | 1,492 | 12.3 | 1,604 | 12.9 | 1,455 | 12.7 |
| **Sex** | | | **Male** | | | **36,229** | **51.4** | 5,702 | 53.1 | 6,074 | 51.6 | 6,217 | 52.1 | 6,303 | 52.0 | 6,279 | 50.5 | 5,654 | 49.5 |
|  |  |  | **Female** | | | **34,229** | **48.6** | 5,043 | 46.9 | 5,706 | 48.4 | 5,714 | 47.9 | 5,825 | 48.0 | 6,165 | 49.5 | 5,776 | 50.5 |
| **Race** | | | **White** | | | **66,312** | **94.1** | 10,207 | 95.0 | 11,118 | 94.4 | 11,159 | 93.5 | 11,355 | 93.6 | 11,753 | 94.4 | 10,720 | 93.8 |
|  |  |  | **Mixed** | | | **163** | **0.2** | 25 | 0.2 | 25 | 0.2 | 21 | 0.2 | 30 | 0.2 | 30 | 0.2 | 32 | 0.3 |
|  |  |  | **Asian** | | | **1,183** | **1.7** | 178 | 1.7 | 187 | 1.6 | 186 | 1.6 | 199 | 1.6 | 233 | 1.9 | 200 | 1.7 |
|  |  |  | **Black** | | | **625** | **0.9** | 77 | 0.7 | 100 | 0.8 | 104 | 0.9 | 108 | 0.9 | 115 | 0.9 | 121 | 1.1 |
|  |  |  | **Other** | | | **518** | **0.7** | 67 | 0.6 | 85 | 0.7 | 75 | 0.6 | 80 | 0.7 | 111 | 0.9 | 100 | 0.9 |
|  |  |  | **Missing** | | | **1,657** | **2.4** | 191 | 1.8 | 265 | 2.2 | 386 | 3.2 | 356 | 2.9 | 202 | 1.6 | 257 | 2.2 |
| **Income domain of the Index of Multiple Deprivation** | | | **1 - Least deprived** | | | **9,891** | **14.0** | 1,458 | 13.6 | 1,662 | 14.1 | 1,653 | 13.9 | 1,649 | 13.6 | 1,801 | 14.5 | 1,668 | 14.6 |
|  |  |  | **2** | | | **12,467** | **17.7** | 1,837 | 17.1 | 2,112 | 17.9 | 2,118 | 17.8 | 2,132 | 17.6 | 2,257 | 18.1 | 2,011 | 17.6 |
|  |  |  | **3** | | | **13,612** | **19.3** | 2,092 | 19.5 | 2,196 | 18.6 | 2,360 | 19.8 | 2,387 | 19.7 | 2,332 | 18.7 | 2,245 | 19.6 |
|  |  |  | **4** | | | **15,262** | **21.7** | 2,378 | 22.1 | 2,610 | 22.2 | 2,515 | 21.1 | 2,620 | 21.6 | 2,689 | 21.6 | 2,450 | 21.4 |
|  |  |  | **5 - Most deprived** | | | **19,226** | **27.3** | 2,980 | 27.7 | 3,200 | 27.2 | 3,285 | 27.5 | 3,340 | 27.5 | 3,365 | 27.0 | 3,056 | 26.7 |
| **Charlson comorbidity index excluding CVD^1^** | | | | | **0** | **33,258** | **47.2** | 5,034 | 46.8 | 5,573 | 47.3 | 5,643 | 47.3 | 5,790 | 47.7 | 5,790 | 46.5 | 5,428 | 47.5 |
|  |  |  |  |  | **1** | **6,020** | **8.5** | 970 | 9.0 | 1,058 | 9.0 | 1,054 | 8.8 | 999 | 8.2 | 1,010 | 8.1 | 929 | 8.1 |
|  |  |  |  |  | **2** | **17,203** | **24.4** | 2,589 | 24.1 | 2,856 | 24.2 | 2,926 | 24.5 | 2,953 | 24.3 | 3,076 | 24.7 | 2,803 | 24.5 |
|  |  |  |  |  | **3** | **7,278** | **10.3** | 1,111 | 10.3 | 1,237 | 10.5 | 1,187 | 9.9 | 1,256 | 10.4 | 1,315 | 10.6 | 1,172 | 10.3 |
|  |  |  |  |  | **≥4** | **6,283** | **8.9** | 1,004 | 9.3 | 1,031 | 8.8 | 1,084 | 9.1 | 1,074 | 8.9 | 1,087 | 8.7 | 1,003 | 8.8 |
|  |  |  |  |  | **Missing^2^** | **416** | **0.6** | 37 | 0.3 | 25 | 0.2 | 37 | 0.3 | 56 | 0.5 | 166 | 1.3 | 95 | 0.8 |
| **TNM stage** | | | **I** | | | **33,890** | **48.1** | 4,704 | 43.8 | 5,418 | 46.0 | 5,649 | 47.3 | 5,857 | 48.3 | 6,252 | 50.2 | 6,010 | 52.6 |
|  |  |  | **II** | | | **15,322** | **21.7** | 2,491 | 23.2 | 2,614 | 22.2 | 2,620 | 22.0 | 2,575 | 21.2 | 2,557 | 20.5 | 2,465 | 21.6 |
|  |  |  | **IIIA** | | | **21,246** | **30.2** | 3,550 | 33.0 | 3,748 | 31.8 | 3,662 | 30.7 | 3,696 | 30.5 | 3,635 | 29.2 | 2,955 | 25.9 |
| **Laterality** | | | **Left** | | | **29,043** | **41.2** | 4,353 | 40.5 | 4,876 | 41.4 | 4,905 | 41.1 | 5,049 | 41.6 | 5,048 | 40.6 | 4,812 | 42.1 |
|  |  |  | **Right** | | | **40,480** | **57.5** | 6,094 | 56.7 | 6,665 | 56.6 | 6,897 | 57.8 | 6,974 | 57.5 | 7,316 | 58.8 | 6,534 | 57.2 |
|  |  |  | **Bilateral** | | | **122** | **0.2** | 12 | 0.1 | 30 | 0.3 | 16 | 0.1 | 21 | 0.2 | 21 | 0.2 | 22 | 0.2 |
|  |  |  | **Missing** | | | **813** | **1.2** | 286 | 2.7 | 209 | 1.8 | 113 | 0.9 | 84 | 0.7 | 59 | 0.5 | 62 | 0.5 |
| **Grade of differentiation** | | **Well differentiated** | | | | **45,189** | **64.1** | 6,286 | 58.5 | 7,196 | 61.1 | 7,490 | 62.8 | 7,874 | 64.9 | 8,440 | 67.8 | 7,903 | 69.1 |
|  |  | **Moderately differentiated** | | | | **2,878** | **4.1** | 459 | 4.3 | 499 | 4.2 | 490 | 4.1 | 515 | 4.2 | 484 | 3.9 | 431 | 3.8 |
|  |  | **Poorly differentiated** | | | | **10,263** | **14.6** | 1,847 | 17.2 | 1,905 | 16.2 | 1,836 | 15.4 | 1,670 | 13.8 | 1,573 | 12.6 | 1,432 | 12.5 |
|  |  | **Undifferentiated / anaplastic** | | | | **11,560** | **16.4** | 2,056 | 19.1 | 2,079 | 17.6 | 2,006 | 16.8 | 1,980 | 16.3 | 1,856 | 14.9 | 1,583 | 13.8 |
|  |  | **Not appropriate or cannot be assessed** | | | | **270** | **0.4** | 60 | 0.6 | 42 | 0.4 | 46 | 0.4 | 41 | 0.3 | 38 | 0.3 | 43 | 0.4 |
|  |  | **Missing** | | | | **298** | **0.4** | 37 | 0.3 | 59 | 0.5 | 63 | 0.5 | 48 | 0.4 | 53 | 0.4 | 38 | 0.3 |
| **Histology** | **Adenocarcinoma** | | | | | **21,863** | **31.0** | 3,488 | 32.5 | 3,884 | 33.0 | 3,911 | 32.8 | 3,526 | 29.1 | 3,627 | 29.1 | 3,427 | 30.0 |
|  | **Squamous cell carcinoma** | | | | | **19,616** | **27.8** | 3,233 | 30.1 | 3,332 | 28.3 | 3,334 | 27.9 | 3,453 | 28.5 | 3,392 | 27.3 | 2,872 | 25.1 |
|  | **Other** | | | | | **28,979** | **41.1** | 4,024 | 37.4 | 4,564 | 38.7 | 4,686 | 39.3 | 5,149 | 42.5 | 5,425 | 43.6 | 5,131 | 44.9 |
| **Treatment modality^3^** | | | | **Surgery** | | **27,819** | **39.5** | 4,334 | 40.3 | 4,679 | 39.7 | 4,818 | 40.4 | 4,585 | 37.8 | 4,784 | 38.4 | 4,619 | 40.4 |
|  |  |  |  | **Radiotherapy** | | **20,196** | **28.7** | 3,087 | 28.7 | 3,435 | 29.2 | 3,519 | 29.5 | 3,519 | 29.0 | 3,448 | 27.7 | 3,188 | 27.9 |
|  |  |  |  | **Chemotherapy** | | **13,691** | **19.4** | 2,294 | 21.3 | 2,462 | 20.9 | 2,490 | 20.9 | 2,224 | 18.3 | 2,327 | 18.7 | 1,894 | 16.6 |

^1^Hospital Episode Statistics assessed 5 years before cancer diagnosis.

^2^Missing if not linked to Hospital Episode Statistics.

^3^All treatments identified using National Cancer Registration and Analysis Service treatment standard operating procedure (between 1 month before and 12 months after cancer diagnosis).

*Abbreviations: CVD: cardiovascular disease.*

**Supplementary Table 12 – Patient, disease and tumour characteristics in the diffuse large B cell lymphoma cohort (N=23,426).^1^**

| **Variable** | **Category** | | **Overall** | | **Years** | | | | | | | | | | | |
| --- | --- | --- | --- | --- | --- | --- | --- | --- | --- | --- | --- | --- | --- | --- | --- | --- |
|  |  |  |  |  | **2013** | | **2014** | | **2015** | | **2016** | | **2017** | | **2018** | |
|  |  |  | **N** | **%** | **N** | **%** | **N** | **%** | **N** | **%** | **N** | **%** | **N** | **%** | **N** | **%** |
|  |  |  | **23,426** |  | **3,366** |  | **3,723** |  | **4,112** |  | **4,248** |  | **4,246** |  | **3,731** |  |
| **Age at cancer diagnosis (years)** | **25-34** | | **652** | **2.8** | 72 | 2.1 | 105 | 2.8 | 118 | 2.9 | 115 | 2.7 | 119 | 2.8 | 123 | 3.3 |
|  | **35-44** | | **994** | **4.2** | 131 | 3.9 | 184 | 4.9 | 180 | 4.4 | 170 | 4.0 | 165 | 3.9 | 164 | 4.4 |
|  | **45-54** | | **2,214** | **9.5** | 333 | 9.9 | 360 | 9.7 | 361 | 8.8 | 425 | 10.0 | 381 | 9.0 | 354 | 9.5 |
|  | **55-64** | | **3,975** | **17.0** | 590 | 17.5 | 643 | 17.3 | 705 | 17.1 | 702 | 16.5 | 697 | 16.4 | 638 | 17.1 |
|  | **65-74** | | **7,138** | **30.5** | 1,039 | 30.9 | 1,113 | 29.9 | 1,232 | 30.0 | 1,281 | 30.2 | 1,352 | 31.8 | 1,121 | 30.0 |
|  | **75-84** | | **6,364** | **27.2** | 909 | 27.0 | 972 | 26.1 | 1,174 | 28.6 | 1,136 | 26.7 | 1,172 | 27.6 | 1,001 | 26.8 |
|  | **≥85** | | **2,089** | **8.9** | 292 | 8.7 | 346 | 9.3 | 342 | 8.3 | 419 | 9.9 | 360 | 8.5 | 330 | 8.8 |
| **Sex** | **Male** | | **12,981** | **55.4** | 1,833 | 54.5 | 2,060 | 55.3 | 2,286 | 55.6 | 2,383 | 56.1 | 2,316 | 54.5 | 2,103 | 56.4 |
|  | **Female** | | **10,445** | **44.6** | 1,533 | 45.5 | 1,663 | 44.7 | 1,826 | 44.4 | 1,865 | 43.9 | 1,930 | 45.5 | 1,628 | 43.6 |
| **Race** | **White** | | **20,921** | **89.3** | 3,085 | 91.7 | 3,340 | 89.7 | 3,632 | 88.3 | 3,772 | 88.8 | 3,779 | 89.0 | 3,313 | 88.8 |
|  | **Mixed** | | **99** | **0.4** | 14 | 0.4 | 9 | 0.2 | 19 | 0.5 | 17 | 0.4 | 26 | 0.6 | 14 | 0.4 |
|  | **Asian** | | **952** | **4.1** | 114 | 3.4 | 144 | 3.9 | 163 | 4.0 | 177 | 4.2 | 191 | 4.5 | 163 | 4.4 |
|  | **Black** | | **351** | **1.5** | 42 | 1.2 | 68 | 1.8 | 69 | 1.7 | 61 | 1.4 | 60 | 1.4 | 51 | 1.4 |
|  | **Other** | | **311** | **1.3** | 33 | 1.0 | 43 | 1.2 | 55 | 1.3 | 65 | 1.5 | 64 | 1.5 | 51 | 1.4 |
|  | **Missing** | | **792** | **3.4** | 78 | 2.3 | 119 | 3.2 | 174 | 4.2 | 156 | 3.7 | 126 | 3.0 | 139 | 3.7 |
| **Income domain of the Index of Multiple Deprivation** | **1 - Least deprived** | | **5,020** | **21.4** | 739 | 22.0 | 772 | 20.7 | 939 | 22.8 | 911 | 21.4 | 882 | 20.8 | 777 | 20.8 |
|  | **2** | | **5,317** | **22.7** | 784 | 23.3 | 867 | 23.3 | 889 | 21.6 | 938 | 22.1 | 1,010 | 23.8 | 829 | 22.2 |
|  | **3** | | **4,880** | **20.8** | 710 | 21.1 | 792 | 21.3 | 869 | 21.1 | 868 | 20.4 | 856 | 20.2 | 785 | 21.0 |
|  | **4** | | **4,371** | **18.7** | 649 | 19.3 | 684 | 18.4 | 754 | 18.3 | 787 | 18.5 | 794 | 18.7 | 703 | 18.8 |
|  | **5 - Most deprived** | | **3,838** | **16.4** | 484 | 14.4 | 608 | 16.3 | 661 | 16.1 | 744 | 17.5 | 704 | 16.6 | 637 | 17.1 |
| **Charlson comorbidity index excluding CVD^2^** | **0** | | **11,051** | **47.2** | 1,570 | 46.6 | 1,727 | 46.4 | 1,953 | 47.5 | 2,017 | 47.5 | 1,996 | 47.0 | 1,788 | 47.9 |
|  | **1** | | **1,840** | **7.9** | 295 | 8.8 | 296 | 8.0 | 326 | 7.9 | 294 | 6.9 | 306 | 7.2 | 323 | 8.7 |
|  | **2** | | **5,805** | **24.8** | 831 | 24.7 | 950 | 25.5 | 1,021 | 24.8 | 1,053 | 24.8 | 1,064 | 25.1 | 886 | 23.7 |
|  | **3** | | **2,504** | **10.7** | 366 | 10.9 | 400 | 10.7 | 429 | 10.4 | 472 | 11.1 | 458 | 10.8 | 379 | 10.2 |
|  | **≥4** | | **2,126** | **9.1** | 292 | 8.7 | 336 | 9.0 | 368 | 8.9 | 400 | 9.4 | 391 | 9.2 | 339 | 9.1 |
|  | **Missing^3^** | | **100** | **0.4** | 12 | 0.4 | 14 | 0.4 | 15 | 0.4 | 12 | 0.3 | 31 | 0.7 | 16 | 0.4 |
| **TNM stage** | **I** | | **4,478** | **19.1** | 790 | 23.5 | 842 | 22.6 | 790 | 19.2 | 708 | 16.7 | 717 | 16.9 | 631 | 16.9 |
|  | **II** | | **3,973** | **17.0** | 601 | 17.9 | 720 | 19.3 | 772 | 18.8 | 722 | 17.0 | 619 | 14.6 | 539 | 14.4 |
|  | **III** | | **4,066** | **17.4** | 619 | 18.4 | 683 | 18.3 | 737 | 17.9 | 734 | 17.3 | 710 | 16.7 | 583 | 15.6 |
|  | **IV** | | **10,909** | **46.6** | 1,356 | 40.3 | 1,478 | 39.7 | 1,813 | 44.1 | 2,084 | 49.1 | 2,200 | 51.8 | 1,978 | 53.0 |
| **Grade of differentiation** | **Well differentiated** | | **14,589** | **62.3** | 2,353 | 69.9 | 2,236 | 60.1 | 2,375 | 57.8 | 2,625 | 61.8 | 2,758 | 65.0 | 2,242 | 60.1 |
|  | **Moderately differentiated** | | **13** | **0.1** | 4 | 0.1 | 3 | 0.1 | 4 | 0.1 | 0 | 0.0 | 2 | 0.0 | 0 | 0.0 |
|  | **Poorly differentiated** | | **28** | **0.1** | 11 | 0.3 | 7 | 0.2 | 2 | 0.0 | 2 | 0.0 | 4 | 0.1 | 2 | 0.1 |
|  | **Undifferentiated / anaplastic** | | **1,260** | **5.4** | 411 | 12.2 | 277 | 7.4 | 287 | 7.0 | 187 | 4.4 | 60 | 1.4 | 38 | 1.0 |
|  | **Not appropriate or cannot be assessed** | | **53** | **0.2** | 13 | 0.4 | 10 | 0.3 | 9 | 0.2 | 4 | 0.1 | 7 | 0.2 | 10 | 0.3 |
|  | **Missing** | | **7,483** | **31.9** | 574 | 17.1 | 1,190 | 32.0 | 1,435 | 34.9 | 1,430 | 33.7 | 1,415 | 33.3 | 1,439 | 38.6 |
| **Treatment modality^4^** | | **Radiotherapy** | **6,564** | **28.0** | 891 | 26.5 | 1,087 | 29.2 | 1,098 | 26.7 | 1,177 | 27.7 | 1,199 | 28.2 | 1,112 | 29.8 |
|  |  | **Chemotherapy** | **19,861** | **84.8** | 2,804 | 83.3 | 3,169 | 85.1 | 3,478 | 84.6 | 3,594 | 84.6 | 3,611 | 85.0 | 3,205 | 85.9 |

^1^No specific histology categories are included in National Cancer Registration and Analysis Service for DLBCL cases.

^2^Hospital Episode Statistics assessed 5 years before cancer diagnosis.

^3^Missing if not linked to Hospital Episode Statistics.

^4^All treatments identified using National Cancer Registration and Analysis Service treatment standard operating procedure (between 1 month before and 12 months after cancer diagnosis).

*Abbreviations: CVD: cardiovascular disease.*

**Supplementary Table 13 – Patient, disease and tumour characteristics in the Hodgkin lymphoma cohort (N=7,303).**

| **Variable** | | | **Category** | | **Overall** | | **Years** | | | | | | | | | | | |
| --- | --- | --- | --- | --- | --- | --- | --- | --- | --- | --- | --- | --- | --- | --- | --- | --- | --- | --- |
|  |  |  |  |  |  |  | **2013** | | **2014** | | **2015** | | **2016** | | **2017** | | **2018** | |
|  |  |  |  |  | **N** | **%** | **N** | **%** | **N** | **%** | **N** | **%** | **N** | **%** | **N** | **%** | **N** | **%** |
|  |  |  |  |  | **7,303** |  | **1,016** |  | **1,186** |  | **1,306** |  | **1,236** |  | **1,331** |  | **1,228** |  |
| **Age at cancer diagnosis (years)** | | | **25-34** | | **1,686** | **23.1** | 225 | 22.1 | 260 | 21.9 | 299 | 22.9 | 302 | 24.4 | 329 | 24.7 | 271 | 22.1 |
|  |  |  | **35-44** | | **1,170** | **16.0** | 177 | 17.4 | 190 | 16.0 | 204 | 15.6 | 196 | 15.9 | 226 | 17.0 | 177 | 14.4 |
|  |  |  | **45-54** | | **1,125** | **15.4** | 164 | 16.1 | 172 | 14.5 | 212 | 16.2 | 184 | 14.9 | 193 | 14.5 | 200 | 16.3 |
|  |  |  | **55-64** | | **1,076** | **14.7** | 153 | 15.1 | 187 | 15.8 | 181 | 13.9 | 179 | 14.5 | 187 | 14.0 | 189 | 15.4 |
|  |  |  | **65-74** | | **1,158** | **15.9** | 139 | 13.7 | 201 | 16.9 | 212 | 16.2 | 196 | 15.9 | 211 | 15.9 | 199 | 16.2 |
|  |  |  | **75-84** | | **883** | **12.1** | 127 | 12.5 | 141 | 11.9 | 161 | 12.3 | 150 | 12.1 | 153 | 11.5 | 151 | 12.3 |
|  |  |  | **≥85** | | **205** | **2.8** | 31 | 3.1 | 35 | 3.0 | 37 | 2.8 | 29 | 2.3 | 32 | 2.4 | 41 | 3.3 |
| **Sex** | | | **Male** | | **4,321** | **59.2** | 580 | 57.1 | 687 | 57.9 | 795 | 60.9 | 717 | 58.0 | 810 | 60.9 | 732 | 59.6 |
|  |  |  | **Female** | | **2,982** | **40.8** | 436 | 42.9 | 499 | 42.1 | 511 | 39.1 | 519 | 42.0 | 521 | 39.1 | 496 | 40.4 |
| **Race** | | | **White** | | **5,964** | **81.7** | 847 | 83.4 | 994 | 83.8 | 1,047 | 80.2 | 996 | 80.6 | 1,086 | 81.6 | 994 | 80.9 |
|  |  |  | **Mixed** | | **81** | **1.1** | 12 | 1.2 | 7 | 0.6 | 17 | 1.3 | 17 | 1.4 | 19 | 1.4 | 9 | 0.7 |
|  |  |  | **Asian** | | **486** | **6.7** | 65 | 6.4 | 61 | 5.1 | 75 | 5.7 | 86 | 7.0 | 111 | 8.3 | 88 | 7.2 |
|  |  |  | **Black** | | **231** | **3.2** | 34 | 3.3 | 33 | 2.8 | 42 | 3.2 | 37 | 3.0 | 43 | 3.2 | 42 | 3.4 |
|  |  |  | **Other** | | **155** | **2.1** | 18 | 1.8 | 35 | 3.0 | 30 | 2.3 | 21 | 1.7 | 28 | 2.1 | 23 | 1.9 |
|  |  |  | **Missing** | | **386** | **5.3** | 40 | 3.9 | 56 | 4.7 | 95 | 7.3 | 79 | 6.4 | 44 | 3.3 | 72 | 5.9 |
| **Income domain of the Index of Multiple Deprivation** | | | **1 - Least deprived** | | **1,322** | **18.1** | 199 | 19.6 | 206 | 17.4 | 232 | 17.8 | 224 | 18.1 | 237 | 17.8 | 224 | 18.2 |
|  |  |  | **2** | | **1,463** | **20.0** | 201 | 19.8 | 252 | 21.2 | 259 | 19.8 | 242 | 19.6 | 267 | 20.1 | 242 | 19.7 |
|  |  |  | **3** | | **1,544** | **21.1** | 188 | 18.5 | 251 | 21.2 | 275 | 21.1 | 278 | 22.5 | 284 | 21.3 | 268 | 21.8 |
|  |  |  | **4** | | **1,531** | **21.0** | 242 | 23.8 | 237 | 20.0 | 273 | 20.9 | 251 | 20.3 | 270 | 20.3 | 258 | 21.0 |
|  |  |  | **5 - Most deprived** | | **1,443** | **19.8** | 186 | 18.3 | 240 | 20.2 | 267 | 20.4 | 241 | 19.5 | 273 | 20.5 | 236 | 19.2 |
| **Charlson comorbidity index excluding CVD^1^** | | | **0** | | **3,512** | **48.1** | 493 | 48.5 | 554 | 46.7 | 610 | 46.7 | 606 | 49.0 | 649 | 48.8 | 600 | 48.9 |
|  |  |  | **1** | | **608** | **8.3** | 90 | 8.9 | 98 | 8.3 | 124 | 9.5 | 105 | 8.5 | 109 | 8.2 | 82 | 6.7 |
|  |  |  | **2** | | **1,729** | **23.7** | 261 | 25.7 | 280 | 23.6 | 324 | 24.8 | 265 | 21.4 | 296 | 22.2 | 303 | 24.7 |
|  |  |  | **3** | | **736** | **10.1** | 95 | 9.4 | 128 | 10.8 | 126 | 9.6 | 135 | 10.9 | 141 | 10.6 | 111 | 9.0 |
|  |  |  | **≥4** | | **641** | **8.8** | 73 | 7.2 | 116 | 9.8 | 113 | 8.7 | 115 | 9.3 | 114 | 8.6 | 110 | 9.0 |
|  |  |  | **Missing^2^** | | **77** | **1.1** | 4 | 0.4 | 10 | 0.8 | 9 | 0.7 | 10 | 0.8 | 22 | 1.7 | 22 | 1.8 |
| **TNM stage** | | | **I** | | **1,006** | **13.8** | 140 | 13.8 | 178 | 15.0 | 185 | 14.2 | 157 | 12.7 | 177 | 13.3 | 169 | 13.8 |
|  |  |  | **II** | | **2,380** | **32.6** | 354 | 34.8 | 386 | 32.5 | 427 | 32.7 | 436 | 35.3 | 427 | 32.1 | 350 | 28.5 |
|  |  |  | **III** | | **1,571** | **21.5** | 205 | 20.2 | 246 | 20.7 | 297 | 22.7 | 271 | 21.9 | 276 | 20.7 | 276 | 22.5 |
|  |  |  | **IV** | | **2,346** | **32.1** | 317 | 31.2 | 376 | 31.7 | 397 | 30.4 | 372 | 30.1 | 451 | 33.9 | 433 | 35.3 |
| **Grade of differentiation** | | **Well differentiated** | | | **6,051** | **82.9** | 921 | 90.6 | 968 | 81.6 | 1,063 | 81.4 | 1,006 | 81.4 | 1,094 | 82.2 | 999 | 81.4 |
|  |  | **Moderately differentiated** | | | **145** | **2.0** | 53 | 5.2 | 27 | 2.3 | 22 | 1.7 | 27 | 2.2 | 12 | 0.9 | 4 | 0.3 |
|  |  | **Poorly differentiated** | | | **58** | **0.8** | 16 | 1.6 | 16 | 1.3 | 14 | 1.1 | 7 | 0.6 | 1 | 0.1 | 4 | 0.3 |
|  |  | **Undifferentiated / anaplastic** | | | **10** | **0.1** | 2 | 0.2 | 2 | 0.2 | 2 | 0.2 | 2 | 0.2 | 1 | 0.1 | 1 | 0.1 |
|  |  | **Not appropriate or cannot be assessed** | | | **14** | **0.2** | 3 | 0.3 | 1 | 0.1 | 1 | 0.1 | 3 | 0.2 | 3 | 0.2 | 3 | 0.2 |
|  |  | **Missing** | | | **1,025** | **14.0** | 21 | 2.1 | 172 | 14.5 | 204 | 15.6 | 191 | 15.5 | 220 | 16.5 | 217 | 17.7 |
| **Histology** | **Classic** | | | | **3,739** | **51.2** | 572 | 56.3 | 647 | 54.6 | 685 | 52.5 | 622 | 50.3 | 638 | 47.9 | 575 | 46.8 |
|  | **Nodular lymphocyte predominant** | | | | **859** | **11.8** | 107 | 10.5 | 124 | 10.5 | 152 | 11.6 | 156 | 12.6 | 146 | 11.0 | 174 | 14.2 |
|  | **Not otherwise specified** | | | | **2,705** | **37.0** | 337 | 33.2 | 415 | 35.0 | 469 | 35.9 | 458 | 37.1 | 547 | 41.1 | 479 | 39.0 |
| **Treatment modality^3^** | | | | **Radiotherapy** | **1,809** | **24.8** | 255 | 25.1 | 301 | 25.4 | 354 | 27.1 | 306 | 24.8 | 299 | 22.5 | 294 | 23.9 |
|  |  |  |  | **Chemotherapy** | **6,212** | **85.1** | 869 | 85.5 | 1,013 | 85.4 | 1,096 | 83.9 | 1,048 | 84.8 | 1,143 | 85.9 | 1,043 | 84.9 |

^1^Hospital Episode Statistics assessed 5 years before cancer diagnosis.

^2^Missing if not linked to Hospital Episode Statistics.

^3^All treatments identified using National Cancer Registration and Analysis Service treatment standard operating procedure (between 1 month before and 12 months after cancer diagnosis).

*Abbreviations: CVD: cardiovascular disease.*

**Supplementary Table 14 – Prevalence of hospitalisation with cardiovascular disease before cancer diagnosis in the overall and individual tumour cohorts.^1^**

| **Variable** | **Full cohort**  **N = 634,240** | **Breast**  **N = 226,516** | **Colon**  **N = 91,210** | **Rectal**  **N = 39,688** | **Prostate**  **N = 175,639** | **NSCLC**  **N = 70,458** | **DLBCL**  **N = 23,426** | **Hodgkin lymphoma**  **N = 7,303** |
| --- | --- | --- | --- | --- | --- | --- | --- | --- |
| **Total with prior CVD, n (%; 95% CI)** | **102,834 (16.2; 16.1, 16.3)** | **17,452 (7.7;  7.6, 7.8)** | **20,161 (22.1; 21.8, 22.4)** | **6,699 (16.9; 16.5, 17.2)** | **27,123 (15.4; 15.3, 15.6)** | **25,458 (36.1; 35.8, 36.5)** | **5,091 (21.7; 21.2, 22.3)** | **850 (11.6; 10.9, 12.4)** |
| **Age at cancer diagnosis (years), n (%; 95% CI)** | | | | | | | | |
| 25-34 | **76 (1.0;  0.8, 1.2)** | 28 (0.7;  0.4, 1.0) | 8 (0.9;  0.3, 1.5) | 3 (0.8;  0.0, 1.7) | 0 (0.0;  0.0, 0.0) | 6 (3.9;  0.8, 6.9) | 16 (2.5;  1.3, 3.6) | 15 (0.9;  0.4, 1.3) |
| 35-44 | **269 (1.2;  1.0, 1.3)** | 148 (0.9;  0.7, 1.0) | 34 (1.7;  1.1, 2.2) | 22 (2.2;  1.3, 3.1) | 6 (1.6;  0.3, 2.9) | 14 (3.2;  1.5, 4.8) | 25 (2.5;  1.5, 3.5) | 20 (1.7;  1.0, 2.5) |
| 45-54 | **2,111 (2.9;  2.7, 3.0)** | 857 (1.7;  1.6, 1.8) | 289 (5.1;  4.5, 5.6) | 143 (4.1;  3.4, 4.7) | 322 (3.8;  3.4, 4.2) | 282 (10.6;  9.4, 11.8) | 155 (7.0;  5.9, 8.1) | 63 (5.6;  4.3, 6.9) |
| 55-64 | **10,240 (7.8;  7.6, 7.9)** | 1,851 (3.6;  3.4, 3.7) | 1,521 (9.9;  9.4, 10.4) | 764 (8.6;  8.0, 9.2) | 3,245 (8.1;  7.9, 8.4) | 2,198 (21.3; 20.5, 22.1) | 526 (13.2;  12.2, 14.3) | 135 (12.5;  10.6, 14.5) |
| 65-74 | **32,867 (15.8; 15.7, 16)** | 4,263 (7.6;  7.4, 7.8) | 5,177 (19.0; 18.5, 19.4) | 1,985 (15.7; 15.1, 16.4) | 11,692 (14.8; 14.5, 15.0) | 8,007 (33.0; 32.4, 33.6) | 1,496 (21.0; 20.0, 21.9) | 247 (21.3;  19.0, 23.7) |
| 75-84 | **40,123 (27.7; 27.5, 28.0)** | 5,939 (18.0; 17.6, 18.4) | 8,709 (30.5; 29.9, 31.0) | 2,662 (26.6; 25.8, 27.5) | 9,948 (23.7;  23.3, 24.1) | 10,557 (44.2; 43.6, 44.8) | 2,020 (31.7; 30.6, 32.9) | 288 (32.6;  29.5, 35.7) |
| ≥85 | **17,148 (37.6; 37.2, 38.1)** | 4,366 (30.5; 29.8, 31.3) | 4,423 (39.1; 38.2, 40.0) | 1,120 (33.9; 32.3, 35.5) | 1,910 (33.6; 32.4, 34.8) | 4,394 (50.5; 49.4, 51.5) | 853 (40.8;  38.7, 42.9) | 82 (40.0;  33.3, 46.7) |
| **Sex, n (%; 95% CI)** | | | | | | | | |
| Male | **63,317 (20.9; 20.8, 21.0)** | - | 12,479 (25.8; 25.4, 26.2) | 4,902 (19.3; 18.8, 19.8) | - | 15,008 (41.4; 40.9, 41.9) | 3,240 (25.0; 24.2, 25.7) | 565 (13.1; 12.1, 14.1) |
| Female | **39,517 (11.9; 11.8, 12.0)** | - | 7,682 (18.0; 17.6, 18.3) | 1,797 (12.6; 12.1, 13.1) | - | 10,450 (30.5; 30.0, 31.0) | 1,851 (17.7; 17.0, 18.5) | 285 (9.6;  8.5, 10.6) |
| **Race, n (%; 95% CI)** | | | | | | | | |
| White | **96,223 (17.0; 16.9, 17.1)** | 16,183 (8.1;  8.0, 8.3) | 19,060 (22.9; 22.6, 23.2) | 6,295 (17.4; 17.0, 17.8) | 24,993 (16.3; 16.1, 16.5) | 24,251 (36.6; 36.2, 36.9) | 4,689 (22.4; 21.8, 23.0) | 752 (12.6;  11.8, 13.5) |
| Mixed | **264 (9.8;  8.7, 10.9)** | 58 (4.9; 3.7, 6.1) | 44 (16.1;  11.7, 20.4) | 19 (14.5;  8.5, 20.5) | 92 (12.1;  9.8, 14.4) | 38 (23.3;  16.8, 29.8) | 12 (12.1;  5.7, 18.6) | 1 (1.2;  0.0, 3.6) |
| Asian | **2,585 (15.3; 14.7, 15.8)** | 580 (7.2;  6.6, 7.8) | 387 (20.6;  18.7, 22.4) | 189 (17.7; 15.4, 20) | 725 (21.9;  20.5, 23.3) | 413 (34.9;  32.2, 37.6) | 233 (24.5;  21.7, 27.2) | 58 (11.9;  9.1, 14.8) |
| Black | **1,343 (9.9;  9.4, 10.4)** | 245 (5.4;  4.8, 6.1) | 205 (15.3;  13.4, 17.2) | 54 (12.9;  9.7, 16.1) | 613 (10.1;  9.3, 10.8) | 171 (27.4;  23.9, 30.9) | 39 (11.1;  7.8, 14.4) | 16 (6.9;  3.7, 10.2) |
| Other | **743 (10.4;  9.7, 11.1)** | 138 (4.4;  3.7, 5.1) | 132 (14.5;  12.2, 16.8) | 38 (9.8;  6.8, 12.8) | 215 (12.5;  10.9, 14.0) | 154 (29.7;  25.8, 33.7) | 54 (17.4;  13.2, 21.6) | 12 (7.7;  3.5, 11.9) |
| **Income domain of the Index of Multiple Deprivation^2^, n (%; 95% CI)** | | | | | | | | |
| 1 - Least | **18,714 (13.3; 13.1, 13.5)** | 3,153 (6.1;  5.9, 6.3) | 3,990 (19.7; 19.1, 20.2) | 1,306 (14.9; 14.1, 15.6) | 5,903 (13.5; 13.2, 13.8) | 3,227 (32.6; 31.7, 33.5) | 986 (19.6;  18.5, 20.7) | 149 (11.3;  9.6, 13.0) |
| 2 | **21,433 (14.8; 14.6, 15.0)** | 3,577 (6.8;  6.6, 7.1) | 4,386 (20.6; 20.0, 21.1) | 1,367 (15.1; 14.4, 15.9) | 6,392 (14.8; 14.5, 15.2) | 4,403 (35.3; 34.5, 36.2) | 1,147 (21.6; 20.5, 22.7) | 161 (11.0;  9.4, 12.6) |
| 3 | **21,089 (16.0; 15.8, 16.2)** | 3,714 (7.8;  7.6, 8.1) | 4,202 (22.2; 21.6, 22.8) | 1,403 (16.8; 16.0, 17.6) | 5,729 (15.5; 15.2, 15.9) | 4,808 (35.3; 34.5, 36.1) | 1,066 (21.8; 20.7, 23.0) | 167 (10.8;  9.3, 12.4) |
| 4 | **20,321 (17.8; 17.6, 18.0)** | 3,534 (8.7;  8.4, 9.0) | 3,877 (23.7; 23.0, 24.3) | 1,331 (18.6; 17.7, 19.5) | 4,758 (16.5; 16.0, 16.9) | 5,631 (36.9; 36.1, 37.7) | 995 (22.8;  21.5, 24.0) | 195 (12.7; 11.1, 14.4) |
| 5 - Most | **21,277 (20.7; 20.5, 21.0)** | 3,474 (10.1;  9.8, 10.4) | 3,706 (25.9; 25.2, 26.6) | 1,292 (20.4; 19.4, 21.4) | 4,341 (18.9; 18.4, 19.4) | 7,389 (38.4; 37.7, 39.1) | 897 (23.4;  22.0, 24.7) | 178 (12.3;  10.6, 14.0) |
| **Charlson comorbidity index^3^, n (%; 95% CI)** | | | | | | | | |
| 0 | **48,708 (16.5; 16.3, 16.6)** | 8,280 (7.8;  7.6, 8.0) | 9,703 (22.4; 22.0, 22.8) | 3,213 (17.0; 16.5, 17.5) | 12,760 (16.0; 15.8, 16.3) | 11,971 (36.0; 35.5, 36.5) | 2,391 (21.6; 20.9, 22.4) | 390 (11.1;  10.1, 12.1) |
| 1 | **8,768 (16.3; 16.0, 16.7)** | 1,485 (7.7;  7.3, 8.1) | 1,707 (22.3; 21.4, 23.3) | 582 (17.3; 16.0, 18.6) | 2,269 (15.3; 14.7, 15.9) | 2,244 (37.3; 36.1, 38.5) | 403 (21.9;  20.0, 23.8) | 78 (12.8;  10.2, 15.5) |
| 2 | **25,259 (16.2; 16.0, 16.4)** | 4,312 (7.8;  7.6, 8.0) | 4,774 (21.2; 20.7, 21.8) | 1,644 (16.9; 16.2, 17.7) | 6,758 (15.6; 15.2, 15.9) | 6,282 (36.5; 35.8, 37.2) | 1,279 (22.0; 21.0, 23.1) | 210 (12.1;  10.6, 13.7) |
| 3 | **10,671 (16.3; 16.0, 16.6)** | 1,825 (7.8;  7.5, 8.2) | 2,138 (23.0; 22.2, 23.9) | 682 (16.7; 15.6, 17.9) | 2,802 (15.3; 14.8, 15.9) | 2,605 (35.8; 34.7, 36.9) | 533 (21.3;  19.7, 22.9) | 86 (11.7;  9.4, 14.0) |
| ≥4 | **9,275 (16.4; 16.1, 16.7)** | 1,542 (7.6;  7.3, 8.0) | 1,821 (22.2; 21.3, 23.1) | 573 (16.2; 15.0, 17.4) | 2,501 (16.1; 15.5, 16.7) | 2,270 (36.1; 34.9, 37.3) | 484 (22.8;  21.0, 24.5) | 84 (13.1;  10.5, 15.7) |
| **Screen-detected, n (%; 95% CI)** | | | | | | | | |
| Yes | **-** | 9,854 (9.9;  9.8, 10.1) | - | - | - | - | - | - |
| No | **-** | 3,311 (4.4;  4.2, 4.5) | - | - | - | - | - | - |
| **TNM stage, n (%; 95% CI)** | | | | | | | | |
|  | **6,1923 (16.3; 16.2, 16.5)** | 7,367 (7.0;  6.9, 7.2) | 4,460 (23.2; 22.6, 23.8) | 2,416 (19.6; 18.9, 20.3) | 12,714 (16.0; 15.7, 16.3) | 12,926 (38.1; 37.6, 38.7) | 924 (20.6;  19.4, 21.8) | 104 (10.3;  8.5, 12.2) |
| II | **40,911 (16.0; 15.9, 16.2)** | 8,381 (8.5;  8.3, 8.6) | 8,291 (22.5; 22.1, 22.9) | 1,724 (18.4; 17.6, 19.2) | 6,198 (13.9; 13.6, 14.3) | 5,344 (34.9; 34.1, 35.6) | 750 (18.9;  17.7, 20.1) | 186 (7.8;  6.7, 8.9) |
| III | **30,874 (14.6; 14.5, 14.8)** | 1,704 (7.5;  7.2, 7.9) | 7,410 (21.1; 20.6, 21.5) | 2,559 (14.2; 13.7, 14.8) | 8,211 (15.9; 15.6, 16.2) | 7,188 (33.8; 33.2, 34.5) | 937 (23.0;  21.8, 24.3) | 228 (14.5;  12.8, 16.3) |
| IV | **28,237 (18.3; 18.1, 18.5)** | - | - | - | - | - | 2,480 (22.7; 21.9, 23.5) | 332 (14.2;  12.7, 15.6) |
| **Laterality, n (%; 95% CI)** | | | | | | | | |
| Left | **19,402 (13.4; 13.3, 13.6)** | 8,973 (7.8;  7.6, 7.9) | - | - | - | 10,429 (35.9; 35.4, 36.5) | - | - |
| Right | **22,817 (15.3; 15.1, 15.5)** | 8,189 (7.5;  7.4, 7.7) | - | - | - | 14,628 (36.1; 35.7, 36.6) | - | - |
| Bilateral | **322 (13.8;  12.4, 15.2)** | 274 (12.3;  11.0, 13.7) | *-* | *-* | *-* | 48 (39.3;  30.7, 48.0) | *-* | *-* |
| **Treatment modality, n (%; 95% CI)** | | | | | | | | |
| Surgery | **42,748 (11.3; 11.2, 11.4)** | 10,975 (5.4;  5.3, 5.5) | 18,006 (21.4; 21.1, 21.7) | 3,570 (13.1; 12.7, 13.5) | 2,846 (7.6;  7.3, 7.8) | 7,351 (26.4; 25.9, 26.9) | - | - |
| Radiotherapy | **29,291 (11.1; 11.0, 11.2)** | 7,347 (4.8;  4.7, 4.9) | 474 (18.2;  16.7, 19.7) | 2,776 (16.2; 15.6, 16.7) | 9,829 (15.8; 15.5, 16.1) | 7,541 (37.3; 36.7, 38.0) | 1,178 (17.9; 17.0, 18.9) | 146 (8.1;  6.8, 9.3) |
| Chemotherapy | **15,255 (9.0;  8.9, 9.2)** | 2,215 (2.8;  2.7, 2.9) | 3,069 (11.3; 10.9, 11.6) | 1,480 (9.4; 9.0, 9.9) | 1,247 (17.7; 16.8, 18.6) | 2,891 (21.1; 20.4, 21.8) | 3,734 (18.8; 18.3, 19.3) | 619 (10.0;  9.2, 10.7) |

^1^Numbers refer to tumour diagnoses (and not to patients).

^2^ Income domain of the Index of Multiple Deprivation derived in 2015 used for patients diagnosed with cancer in 2013 and income domain of the Index of Multiple Deprivation derived in 2019 used for patients diagnosed with cancer after 2013.

^3^ 5 years before diagnosis and excluding cardiovascular disease.

*Abbreviations: NSCLC: non-small cell lung cancer; DLBCL: diffuse large B-cell lymphoma.*

**Supplementary Table 15 – Associations between patient characteristics and cardiovascular disease hospitalisation before cancer diagnosis (unadjusted odds ratios).^1^**

| **Variable** | **Full cohort**  **N = 634,240** | **Breast**  **N = 226,516** | **Colon**  **N = 91,210** | **Rectal**  **N = 39,688** | **Prostate**  **N = 175,639** | **NSCLC**  **N = 70,458** | **DLBCL**  **N = 23,426** | **Hodgkin lymphoma**  **N = 7,303** |
| --- | --- | --- | --- | --- | --- | --- | --- | --- |
| **Total with prior CVD** | **102,834** | **17,452** | **20,161** | **6,699** | **27,123** | **25,458** | **5,091** | **850** |
| **Age at cancer diagnosis (years), OR (95% CI)** | | | | | | | | |
| 25-34 | **0.05 (0.04, 0.07)** | 0.08 (0.06, 0.12) | 0.04 (0.02, 0.08) | 0.04 (0.01, 0.14) | N/A | 0.08 (0.04, 0.19) | 0.09 (0.06, 0.16) | 0.03 (0.02, 0.06) |
| 35-44 | **0.06 (0.06, 0.07)** | 0.10 (0.09, 0.12) | 0.07 (0.05, 0.10) | 0.12 (0.08, 0.18) | 0.10 (0.04, 0.22) | 0.07 (0.04, 0.11) | 0.10 (0.07, 0.15) | 0.06 (0.04, 0.10) |
| 45-54 | **0.16 (0.15, 0.16)** | 0.21 (0.20, 0.23) | 0.23 (0.20, 0.26) | 0.23 (0.19, 0.27) | 0.23 (0.20, 0.25) | 0.24 (0.21, 0.27) | 0.28 (0.24, 0.34) | 0.22 (0.16, 0.29) |
| 55-64 | **0.45 (0.44, 0.46)** | 0.45 (0.42, 0.47) | 0.47 (0.44, 0.50) | 0.51 (0.46, 0.55) | 0.51 (0.49, 0.53) | 0.55 (0.52, 0.58) | 0.58 (0.52, 0.64) | 0.53 (0.42, 0.67) |
| 65-74 | **1 (Reference)** | 1 (Reference) | 1 (Reference) | 1 (Reference) | 1 (Reference) | 1 (Reference) | 1 (Reference) | 1 (Reference) |
| 75-84 | **2.04 (2.01, 2.07)** | 2.66 (2.55, 2.77) | 1.87 (1.80, 1.95) | 1.95 (1.82, 2.08) | 1.79 (1.74, 1.85) | 1.61 (1.55, 1.67) | 1.75 (1.62, 1.90) | 1.79 (1.46, 2.18) |
| ≥85 | **3.20 (3.13, 3.27)** | 5.32 (5.07, 5.58) | 2.74 (2.61, 2.88) | 2.75 (2.52, 3.00) | 2.92 (2.75, 3.09) | 2.07 (1.97, 2.18) | 2.60 (2.35, 2.89) | 2.46 (1.80, 3.36) |
| **Sex, OR (95% CI)** | | | | | | | | |
| Male | **1 (Reference)** | - | 1 (Reference) | 1 (Reference) | - | 1 (Reference) | 1 (Reference) | 1 (Reference) |
| Female | **0.51 (0.51, 0.52)** | - | 0.63 (0.61, 0.65) | 0.60 (0.57, 0.64) | - | 0.62 (0.60, 0.64) | 0.65 (0.61, 0.69) | 0.70 (0.60, 0.82) |
| **Race, OR (95% CI)** | | | | | | | | |
| White | **1 (Reference)** | 1 (Reference) | 1 (Reference) | 1 (Reference) | 1 (Reference) | 1 (Reference) | 1 (Reference) | 1 (Reference) |
| Mixed | **0.53 (0.47, 0.60)** | 0.58 (0.45, 0.76) | 0.64 (0.47, 0.89) | 0.80 (0.49, 1.31) | 0.70 (0.57, 0.88) | 0.53 (0.37, 0.76) | 0.48 (0.26, 0.87) | 0.09 (0.01, 0.62) |
| Asian | **0.88 (0.84, 0.92)** | 0.88 (0.8, 0.96) | 0.87 (0.78, 0.98) | 1.02 (0.87, 1.20) | 1.44 (1.32, 1.57) | 0.93 (0.82, 1.05) | 1.12 (0.96, 1.31) | 0.94 (0.71, 1.25) |
| Black | **0.53 (0.50, 0.57)** | 0.65 (0.57, 0.74) | 0.61 (0.52, 0.71) | 0.70 (0.53, 0.94) | 0.57 (0.53, 0.62) | 0.65 (0.55, 0.78) | 0.43 (0.31, 0.60) | 0.52 (0.31, 0.86) |
| Other | **0.57 (0.53, 0.61)** | 0.52 (0.44, 0.62) | 0.57 (0.47, 0.69) | 0.51 (0.37, 0.72) | 0.73 (0.63, 0.84) | 0.73 (0.61, 0.89) | 0.73 (0.54, 0.98) | 0.58 (0.32, 1.05) |
| **Income domain of the Index of Multiple Deprivation^2^, OR (95% CI)** | | | | | | | | |
| 1 - Least | **1 (Reference)** | 1 (Reference) | 1 (Reference) | 1 (Reference) | 1 (Reference) | 1 (Reference) | 1 (Reference) | 1 (Reference) |
| 2 | **1.13 (1.11, 1.16)** | 1.13 (1.08, 1.19) | 1.05 (1.01, 1.11) | 1.02 (0.94, 1.11) | 1.12 (1.08, 1.16) | 1.13 (1.07, 1.19) | 1.13 (1.02, 1.24) | 0.97 (0.77, 1.23) |
| 3 | **1.25 (1.22, 1.27)** | 1.31 (1.25, 1.38) | 1.16 (1.11, 1.22) | 1.15 (1.06, 1.25) | 1.18 (1.13, 1.23) | 1.13 (1.07, 1.19) | 1.14 (1.04, 1.26) | 0.95 (0.76, 1.21) |
| 4 | **1.41 (1.38, 1.44)** | 1.47 (1.40, 1.55) | 1.26 (1.20, 1.33) | 1.30 (1.20, 1.42) | 1.27 (1.21, 1.32) | 1.21 (1.14, 1.27) | 1.21 (1.09, 1.33) | 1.15 (0.92, 1.44) |
| 5 - Most | **1.71 (1.67, 1.75)** | 1.73 (1.65, 1.82) | 1.43 (1.36, 1.50) | 1.46 (1.34, 1.59) | 1.49 (1.43, 1.56) | 1.29 (1.22, 1.36) | 1.25 (1.13, 1.38) | 1.11 (0.88, 1.40) |
| **Charlson comorbidity index^3^, OR (95% CI)** | | | | | | | | |
| 0 | **1 (Reference)** | 1 (Reference) | 1 (Reference) | 1 (Reference) | 1 (Reference) | 1 (Reference) | 1 (Reference) | 1 (Reference) |
| 1 | **0.99 (0.97, 1.02)** | 0.98 (0.93, 1.04) | 1.00 (0.94, 1.06) | 1.02 (0.93, 1.13) | 0.94 (0.90, 0.99) | 1.06 (1.00, 1.12) | 1.02 (0.90, 1.14) | 1.18 (0.91, 1.53) |
| 2 | **0.98 (0.97, 1.00)** | 1.00 (0.96, 1.04) | 0.94 (0.90, 0.97) | 1.00 (0.93, 1.06) | 0.97 (0.94, 1.00) | 1.02 (0.98, 1.06) | 1.02 (0.95, 1.11) | 1.11 (0.93, 1.32) |
| 3 | **0.99 (0.97, 1.01)** | 1.00 (0.95, 1.06) | 1.04 (0.98, 1.09) | 0.98 (0.90, 1.07) | 0.95 (0.91, 0.99) | 0.99 (0.94, 1.05) | 0.98 (0.88, 1.09) | 1.06 (0.83, 1.36) |
| ≥4 | **1.00 (0.97, 1.02)** | 0.98 (0.92, 1.03) | 0.99 (0.94, 1.05) | 0.95 (0.86, 1.04) | 1.00 (0.96, 1.05) | 1.01 (0.95, 1.06) | 1.07 (0.96, 1.19) | 1.21 (0.94, 1.55) |
| **Screen-detected, OR (95% CI)** | | | | | | | | |
| Yes | **-** | 0.41 (0.40, 0.43) | - | - | - | - | - | - |
| No | **-** | 1 (Reference) | - | - | - | - | - | - |
| **TNM stage, OR (95% CI)** | | | | | | | | |
| I | **1 (Reference)** | 1 (Reference) | 1 (Reference) | 1 (Reference) | 1 (Reference) | 1 (Reference) | 1 (Reference) | 1 (Reference) |
| II | **0.90 (0.88, 0.91)** | 1.22 (1.19, 1.27) | 0.96 (0.92, 1.00) | 0.93 (0.87, 0.99) | 0.85 (0.82, 0.88) | 0.87 (0.83, 0.90) | 0.90 (0.80, 1.00) | 0.74 (0.57, 0.95) |
| III | **1.17 (1.15, 1.19)** | 1.08 (1.02, 1.14) | 0.88 (0.85, 0.92) | 0.68 (0.64, 0.73) | 0.99 (0.96, 1.02) | 0.83 (0.80, 0.86) | 1.15 (1.04, 1.28) | 1.47 (1.15, 1.88) |
| IV | **1.41 (1.35, 1.47)** | - | - | - | - | - | 1.13 (1.04, 1.23) | 1.43 (1.13, 1.81) |
| **Laterality, OR (95% CI)** | | | | | | | | |
| Left | **1 (Reference)** | 1 (Reference) | - | - | - | 1 (Reference) | - | - |
| Right | **1.16 (1.14, 1.19)** | 0.96 (0.93, 0.99) | - | - | - | 1.01 (0.98, 1.04) | - | - |
| Bilateral | **1.03 (0.91, 1.16)** | 1.67 (1.47, 1.90) | *-* | *-* | *-* | 1.16 (0.80, 1.67) | *-* | *-* |
| **Treatment modality (“No treatment” reference for each treatment type)^4^, OR (95% CI)** | | | | | | | | |
| Surgery | **0.41 (0.41, 0.42)** | 0.16 (0.16, 0.17) | 0.61 (0.58, 0.64) | 0.45 (0.42, 0.47) | 0.38 (0.37, 0.40) | 0.49 (0.47, 0.50) | - | - |
| Radiotherapy | **0.50 (0.50, 0.51)** | 0.31 (0.30, 0.32) | 0.78 (0.71, 0.86) | 0.91 (0.87, 0.96) | 1.05 (1.02, 1.08) | 1.08 (1.04, 1.11) | 0.72 (0.67, 0.78) | 0.60 (0.50, 0.72) |
| Chemotherapy | **0.43 (0.42, 0.44)** | 0.25 (0.24, 0.26) | 0.35 (0.33, 0.36) | 0.37 (0.35, 0.40) | 1.19 (1.12, 1.26) | 0.41 (0.39, 0.42) | 0.38 (0.35, 0.41) | 0.41 (0.35, 0.49) |

^1^Numbers refer to tumour diagnoses (and not to patients).

^2^ Income domain of the Index of Multiple Deprivation derived in 2015 used for patients diagnosed with cancer in 2013 and income domain of the Index of Multiple Deprivation derived in 2019 used for patients diagnosed with cancer after 2013.

^3^ 5 years before diagnosis and excluding cardiovascular disease.

^4^ The three treatment modalities are not mutually exclusive, and reference includes either “No surgery” or “No chemotherapy” or “No radiotherapy”.

*Abbreviations: NSCLC: non-small cell lung cancer; DLBCL: diffuse large B-cell lymphoma.*

**Supplementary Table 16 – Unadjusted odds of hospitalisation with cardiovascular disease (CVD) before cancer diagnosis in the individual tumour cohorts using logistic regression analysis with interaction between each covariate and the income domain of Index of Multiple Deprivation (N = 634,240).**

| **Income domain of the Index of Multiple Deprivation** | **1 - least** | **2** | **3** | **4** | **5 – most** |
| --- | --- | --- | --- | --- | --- |
| **Breast cancer, N (226,516)** | 51,814 | 52,228 | 47,406 | 40,605 | 34,463 |
| **Prior CVD, n (%) 17,452 (7.7)** | 3,153 (6.1) | 3,577 (6.8) | 3,714 (7.8) | 3,534 (8.7) | 3,474 (10.1) |
| **TNM stage, OR (95% CI)** | | | | | |
| I | 1 (Reference) | 1.12 (1.03, 1.20) | 1.32 (1.23, 1.42) | 1.54 (1.42, 1.66) | 1.84 (1.69, 1.98) |
| II | 1.26 (1.17, 1.36) | 1.46 (1.35, 1.57) | 1.60 (1.48, 1.71) | 1.76 (1.63, 1.90) | 2.09 (1.93, 2.24) |
| III | 1.07 (0.93, 1.21) | 1.18 (1.03, 1.33) | 1.56 (1.38, 1.74) | 1.58 (1.39, 1.77) | 1.72 (1.51, 1.93) |
| **Treatment modality (Reference: “No” treatment^*^ and income domain of the Index of Multiple Deprivation “1”), OR (95% CI)** | | | | | |
| Surgery | 0.16 (0.15, 0.18) | 0.18 (0.17, 0.20) | 0.21 (0.19, 0.22) | 0.23 (0.21, 0.25) | 0.28 (0.26, 0.30) |
| Radiotherapy | 0.33 (0.31, 0.36) | 0.36 (0.33, 0.38) | 0.42 (0.39, 0.45) | 0.47 (0.43, 0.50) | 0.56 (0.52, 0.60) |
| Chemotherapy | 0.25 (0.22, 0.27) | 0.28 (0.25, 0.31) | 0.33 (0.30, 0.37) | 0.39 (0.35, 0.43) | 0.44 (0.40, 0.48) |
|  |  |  |  |  |  |
| **Colon cancer, N (91,210)** | 20,257 | 21,337 | 18,932 | 16,392 | 14,292 |
| **Prior CVD, n (%)20,161 (22.1)** | 3,990 (19.7) | 4,386 (20.6) | 4,202 (22.2) | 3,877 (23.7) | 3,706 (25.9) |
| **TNM stage, OR (95% CI)** | | | | | |
| I | 1 (Reference) | 1.13 (1.01, 1.24) | 1.21 (1.08, 1.33) | 1.39 (1.24, 1.54) | 1.50 (1.34, 1.67) |
| II | 1.03 (0.93, 1.12) | 1.05 (0.95, 1.14) | 1.16 (1.06, 1.27) | 1.28 (1.16, 1.40) | 1.43 (1.29, 1.56) |
| III | 0.92 (0.84, 1.01) | 0.98 (0.89, 1.07) | 1.09 (0.98, 1.19) | 1.13 (1.02, 1.23) | 1.33 (1.20, 1.46) |
| **Treatment modality (Reference: “No” treatment^*^ and income domain of Index of Multiple Deprivation “1”), OR (95% CI)** | | | | | |
| Surgery | 0.67 (0.59, 0.75) | 0.70 (0.62, 0.79) | 0.76 (0.67, 0.85) | 0.84 (0.74, 0.94) | 0.94 (0.83, 1.06) |
| Chemotherapy | 0.37 (0.34, 0.41) | 0.39 (0.35, 0.42) | 0.40 (0.36, 0.43) | 0.42 (0.38, 0.46) | 0.49 (0.44, 0.53) |
|  |  |  |  |  |  |
| **Rectal cancer, N (39,688)** | 8,776 | 9,039 | 8,361 | 7,171 | 6,341 |
| **Prior CVD, n (%) 6,699 (16.9)** | 1,306 (14.9) | 1,367 (15.1) | 1,403 (16.8) | 1,331 (18.6) | 1,292 (20.4) |
| **TNM stage, OR (95% CI)** | | | | | |
| I | 1 (Reference) | 0.98 (0.84, 1.11) | 1.15 (0.99, 1.30) | 1.32 (1.13, 1.50) | 1.48 (1.26, 1.69) |
| II | 0.91 (0.77, 1.05) | 0.91 (0.77, 1.04) | 1.05 (0.89, 1.20) | 1.20 (1.02, 1.38) | 1.38 (1.16, 1.59) |
| III | 0.65 (0.56, 0.74) | 0.71 (0.62, 0.80) | 0.77 (0.67, 0.87) | 0.86 (0.75, 0.98) | 0.98 (0.85, 1.11) |
| **Treatment modality (Reference: “No” treatment^*^ and income domain of the Index of Multiple Deprivation “1”), OR (95% CI)** | | | | | |
| Surgery | 0.49 (0.43, 0.55) | 0.48 (0.42, 0.54) | 0.51 (0.45, 0.57) | 0.60 (0.53, 0.68) | 0.66 (0.58, 0.75) |
| Radiotherapy | 0.91 (0.80, 1.03) | 0.98 (0.86, 1.09) | 1.08 (0.95, 1.21) | 1.10 (0.97, 1.23) | 1.29 (1.14, 1.44) |
| Chemotherapy | 0.40 (0.34, 0.45) | 0.43 (0.37, 0.48) | 0.41 (0.35, 0.47) | 0.47 (0.40, 0.54) | 0.54 (0.46, 0.62) |
|  |  |  |  |  |  |
| **Prostate cancer, N (175,639)** | 43,793 | 43,060 | 36,888 | 28,899 | 22,999 |
| **Prior CVD, n (%) 27,123 (15.4)** | 5,903 (13.5) | 6,392 (14.8) | 5,729 (15.5) | 4,758 (16.5) | 4,341 (18.9) |
| **TNM stage, OR (95% CI)** | | | | | |
| I | 1 (Reference) | 1.16 (1.09, 1.22) | 1.22 (1.15, 1.29) | 1.27 (1.19, 1.35) | 1.53 (1.44, 1.63) |
| II | 0.86 (0.80, 0.92) | 0.94 (0.88, 1.01) | 1.01 (0.94, 1.08) | 1.20 (1.11, 1.28) | 1.26 (1.16, 1.36) |
| III | 1.05 (0.98, 1.11) | 1.13 (1.06, 1.21) | 1.18 (1.10, 1.26) | 1.22 (1.13, 1.30) | 1.52 (1.41, 1.63) |
| **Treatment modality (Reference: “No” treatment^*^ and income domain of the Index of Multiple Deprivation “1”), OR (95% CI)** | | | | | |
| Surgery | 0.40 (0.37, 0.44) | 0.44 (0.40, 0.48) | 0.43 (0.39, 0.47) | 0.47 (0.43, 0.52) | 0.56 (0.51, 0.62) |
| Radiotherapy | 1.14 (1.07, 1.20) | 1.21 (1.14, 1.27) | 1.24 (1.17, 1.32) | 1.39 (1.30, 1.47) | 1.53 (1.43, 1.64) |
| Chemotherapy | 1.21 (1.04, 1.38) | 1.26 (1.09, 1.43) | 1.39 (1.20, 1.58) | 1.44 (1.23, 1.65) | 1.74 (1.50, 1.98) |
|  |  |  |  |  |  |
| **NSCLC, N (70,458)** | 9,891 | 12,467 | 13,612 | 15,262 | 19,226 |
| **Prior CVD, n (%) 25,458 (36.1)** | 3,227 (32.6) | 4,403 (35.3) | 4,808 (35.3) | 5,631 (36.9) | 7,389 (38.4) |
| **TNM stage, OR (95% CI)** | | | | | |
| I | 1 (Reference) | 1.16 (1.07, 1.25) | 1.17 (1.08, 1.26) | 1.24 (1.14, 1.33) | 1.37 (1.28, 1.47) |
| II | 0.99 (0.88, 1.10) | 1.04 (0.94, 1.14) | 0.99 (0.89, 1.08) | 1.06 (0.96, 1.16) | 1.13 (1.03, 1.22) |
| III | 0.84 (0.75, 0.92) | 0.95 (0.87, 1.04) | 0.99 (0.90, 1.08) | 1.07 (0.98, 1.16) | 1.08 (0.99, 1.16) |
| **Treatment modality (Reference: “No” treatment^*^ and income domain of the Index of Multiple Deprivation “1”), OR (95% CI)** | | | | | |
| Surgery | 0.46 (0.42, 0.50) | 0.52 (0.48, 0.57) | 0.52 (0.48, 0.56) | 0.53 (0.49, 0.58) | 0.62 (0.57, 0.66) |
| Radiotherapy | 1.19 (1.08, 1.30) | 1.22 (1.11, 1.32) | 1.28 (1.17, 1.38) | 1.33 (1.22, 1.43) | 1.35 (1.26, 1.45) |
| Chemotherapy | 0.40 (0.35, 0.44) | 0.49 (0.44, 0.54) | 0.47 (0.42, 0.52) | 0.50 (0.45, 0.54) | 0.48 (0.44, 0.53) |
|  |  |  |  |  |  |
| **DLBCL cancer, N (23,426)** | 5,020 | 5,317 | 4,880 | 4,371 | 3,838 |
| **Prior CVD, n (%) 5,091 (21.7)** | 986 (19.6) | 1,147 (21.6) | 1,066 (21.8) | 995 (22.8) | 897 (23.4) |
| **TNM stage, OR (95% CI)** | | | | | |
| I | 1 (Reference) | 1.03 (0.80, 1.25) | 1.10 (0.86, 1.35) | 1.22 (0.94, 1.50) | 1.08 (0.82, 1.35) |
| II | 0.80 (0.61, 0.99) | 0.95 (0.73, 1.17) | 1.00 (0.76, 1.23) | 1.04 (0.79, 1.29) | 1.10 (0.83, 1.37) |
| III | 1.08 (0.84, 1.33) | 1.36 (1.06, 1.65) | 1.24 (0.96, 1.51) | 1.27 (0.97, 1.56) | 1.29 (0.98, 1.60) |
| IV | 1.08 (0.88, 1.28) | 1.19 (0.97, 1.41) | 1.22 (0.99, 1.44) | 1.27 (1.03, 1.51) | 1.39 (1.13, 1.65) |
| **Treatment modality (Reference: “No” treatment^*^ and income domain of the Index of Multiple Deprivation “1”), OR (95% CI)** | | | | | |
| Radiotherapy | 0.81 (0.68, 0.94) | 0.85 (0.72, 0.97) | 0.82 (0.69, 0.95) | 0.89 (0.74, 1.04) | 0.87 (0.72, 1.03) |
| Chemotherapy | 0.34 (0.28, 0.40) | 0.39 (0.32, 0.45) | 0.40 (0.33, 0.47) | 0.41 (0.34, 0.48) | 0.44 (0.36, 0.51) |
|  |  |  |  |  |  |
| **Hodgkin Lymphoma, N (7,303)** | 1,322 | 1,463 | 1,544 | 1,531 | 1,443 |
| **Prior CVD, n (%) 850 (11.6)** | 149 (11.3) | 161 (11.0) | 167 (10.8) | 195 (12.7) | 178 (12.3) |
| **TNM stage, OR (95% CI)** | | | | | |
| I | 1 (Reference) | 1.01 (0.33, 1.69) | 0.92 (0.30, 1.55) | 1.21 (0.42, 1.99) | 1.24 (0.44, 2.05) |
| II | 0.72 (0.28, 1.16) | 0.71 (0.29, 1.14) | 0.74 (0.31, 1.18) | 0.81 (0.34, 1.29) | 0.99 (0.41, 1.56) |
| III | 1.66 (0.69, 2.63) | 1.49 (0.60, 2.38) | 1.22 (0.50, 1.94) | 1.82 (0.80, 2.85) | 1.76 (0.75, 2.77) |
| IV | 1.47 (0.65, 2.30) | 1.53 (0.69, 2.37) | 1.63 (0.73, 2.52) | 1.69 (0.77, 2.62) | 1.38 (0.62, 2.14) |
| **Treatment modality (Reference: “No” treatment^*^ and income domain of the Index of Multiple Deprivation “1”), OR (95% CI)** | | | | | |
| Radiotherapy | 0.76 (0.44, 1.08) | 0.61 (0.36, 0.87) | 0.42 (0.21, 0.62) | 0.66 (0.39, 0.93) | 0.85 (0.50, 1.19) |
| Chemotherapy | 0.46 (0.28, 0.65) | 0.42 (0.25, 0.59) | 0.45 (0.28, 0.63) | 0.49 (0.30, 0.68) | 0.53 (0.32, 0.73) |

*Abbreviations: OR: Odds ratio; CI: Confidence interval; CVD: Cardiovascular disease; NSCLC: Non-small cell lung cancer; DLBCL: Diffuse large B-cell lymphoma.*

^*^The three treatment modalities are not mutually exclusive, and reference includes either “No surgery” or “No chemotherapy” or “No radiotherapy”.

**Supplementary Figure 1 – Venn diagrams showing the overlap between tumours with hospitalised CVD before cancer diagnosis categories identified using ICD-10 diagnosis code list in HES and a record found in a NICOR dataset.^1^**

**N = 102,836**

84,424 (82.1%)

230 (0.2%)


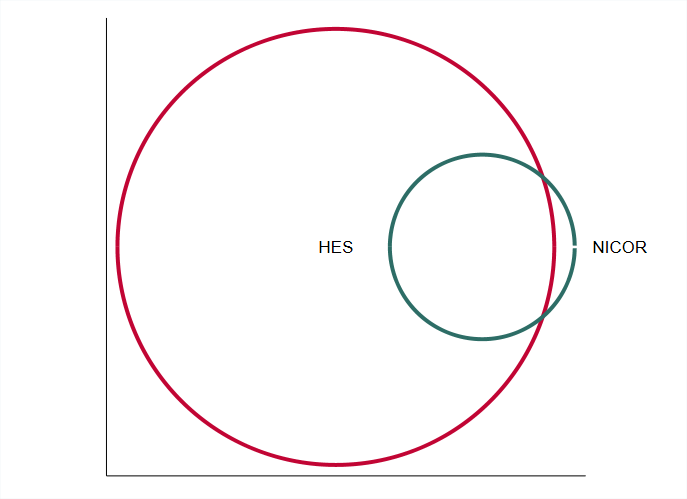


**HES**

18,182

(17.7%)

**NICOR**


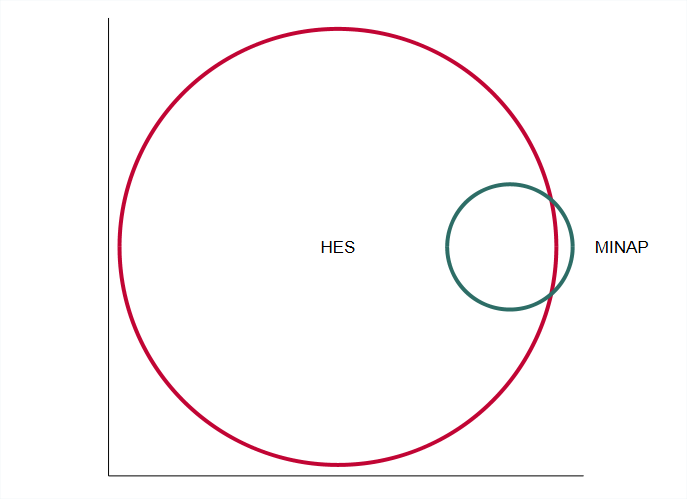

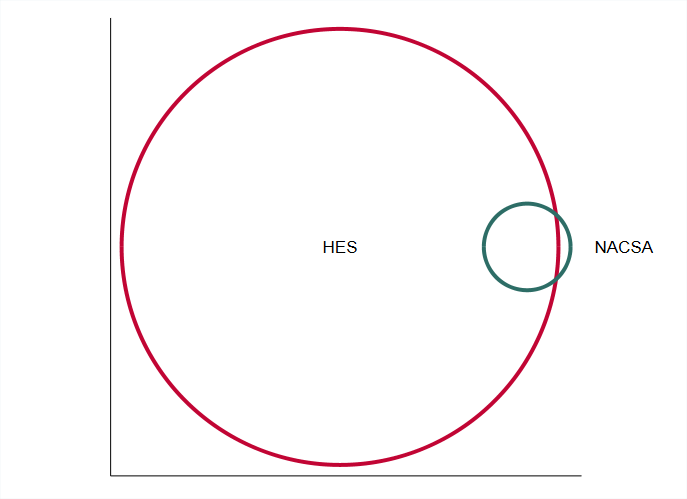

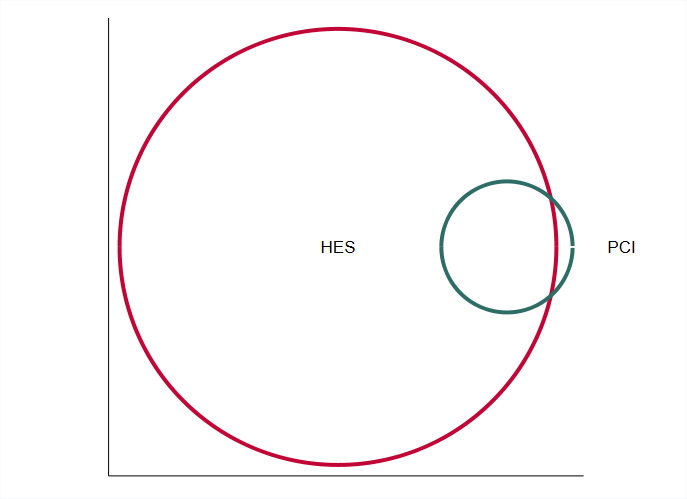

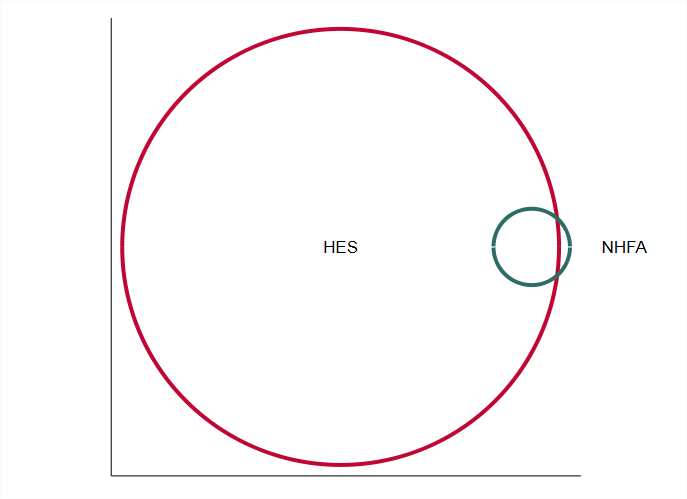


**NAPCI**

99,498 (96.9%)

**MINAP**

4,020 (4.0%)

38 (0.0%)

3,108 (3.0%)

30 (0.0%)

54 (0.1%)

9,250 (9.0%)

**N = 102,636**

**N = 102,660**

**N = 102.644**

93,356 (90.9%)

98,586 (96.0%)

**N = 102,726**

8,359

(8.1%)

120 (0.1%)

94,247 (91.7%)

**HES**

**NHFA**

**HES**

**NACSA**

**HES**

**HES**

*Abbreviations: CVD: cardiovascular disease; HES: Hospital Episode Statistics; MI: myocardial infarction; NICOR: National Institute for Cardiovascular Outcomes Research; MINAP: Myocardial Ischaemia National Audit Project; NACSA: National Adult Cardiac Surgery Audit; NAPCI: National Audit of Percutaneous Coronary Intervention; NHFA: National Heart Failure Audit*

^1^ Each Venn diagram includes patients in either HES or that dataset, so the totals are different across Venn diagram

**References**

1. Henson KE, Elliss-Brookes L, Coupland VH, et al. Data Resource Profile: National Cancer Registration Dataset in England. *Int J Epidemiol*. 2019;49(1):16-16h. doi:10.1093/ije/dyz076

2. Bright CJ, Lawton S, Benson S, et al. Data Resource Profile: The Systemic Anti-Cancer Therapy (SACT) dataset. *Int J Epidemiol*. Feb 1 2020;49(1):15-15l. doi:10.1093/ije/dyz137

3. National Radiotherapy Dataset (RTDS): <http://www.ncin.org.uk/collecting_and_using_data/rtds>. Accessed 05/11/2021, <http://www.ncin.org.uk/collecting_and_using_data/rtds>

4. Herbert A, Wijlaars L, Zylbersztejn A, Cromwell D, Hardelid P. Data Resource Profile: Hospital Episode Statistics Admitted Patient Care (HES APC). *Int J Epidemiol*. 2017;46(4):1093-1093i. doi:10.1093/ije/dyx015

5. National Institute for Cardiovascular Outcomes Research (NICOR): <https://www.nicor.org.uk/>. Accessed 17/02/2021, <https://www.nicor.org.uk/>

6. Wilkinson C, Weston C, Timmis A, Quinn T, Keys A, Gale CP. The Myocardial Ischaemia National Audit Project (MINAP). *Eur Heart J Qual Care Clin Outcomes*. Jan 1 2020;6(1):19-22. doi:10.1093/ehjqcco/qcz052

7. Adult Cardiac Surgery (Surgery Audit). National Institute for Cardiovascular Outcomes Research. Accessed 17/12/2021, 2021. <https://www.nicor.org.uk/adult-cardiac-surgery-surgery-audit/>

8. Adult Percutaneous Coronary Interventions (Angioplasty audit). National Institute for Cardiovascular Outcomes Research. Accessed 17/12/2021, 2021. <https://www.nicor.org.uk/adult-percutaneous-coronary-interventions-angioplasty-audit/>

9. Heart Failure (Heart Failure audit). National Institute for Cardiovascular Outcomes Research. Accessed 17/12/2021, 2021. <https://www.nicor.org.uk/heart-failure-heart-failure-audit/>
